# Deterministic Field‐Free Switching of Perpendicular Magnetization by Bulk Mirror Symmetry Broken Bi_1−_ *_x_* Sb*_x_* (012) with High Spin Hall Conductivity

**DOI:** 10.1002/adma.74710

**Published:** 2026-08-25

**Authors:** Seungwon Rho, Youngmin Lee, Jaeseok Huh, Jaehan Park, Hyeong‐Jun Son, Dowoo Pyeon, Mann‐Ho Cho

**Affiliations:** ^1^ Department of Physics Yonsei University Seoul Republic of Korea; ^2^ Department of Electrical and Computer Engineering National University of Singapore Singapore Singapore; ^3^ Graduate Program of Semiconductor Science and Engineering Yonsei University Seoul Republic of Korea; ^4^ Korea Research Institute of Standards and Science Daejeon Republic of Korea; ^5^ Department of System Semiconductor Engineering Yonsei University Seoul Republic of Korea

**Keywords:** bismuth–antimony alloys, bulk mirror symmetry, field‐free switching, spin Hall angle, spin Hall conductivity, spin–orbit torque

## Abstract

Out‐of‐plane spin–orbit torque generation in mirror‐symmetry‐broken materials is a promising approach for current‐induced field‐free magnetization switching. However, the key challenge lies in identifying materials with high in‐plane spin Hall conductivity that simultaneously generate large out‐of‐plane spin–orbit torque for low‐power spintronic applications. Previously studied low‐symmetry materials, in which mirror‐symmetry breaking is confined to the surface and the spin Hall conductivity is low, have not resolved this challenge. Here, we demonstrate deterministic field‐free magnetization switching enabled by the bulk‐mirror‐symmetry‐broken topological material Bi_1−_ *_x_* Sb*_x_* (012) with high in‐plane spin Hall conductivity. The power consumption is two orders of magnitude lower than that of heavy‐metal‐based devices. Spin‐torque ferromagnetic resonance measurements confirm the bulk origin of the out‐of‐plane spin–orbit torque in Bi_1−_ *_x_* Sb*_x_* (012), yielding an out‐of‐plane spin Hall conductivity of $∼\;1.12\times 10^{5}\frac{\hbar }{2e}\Omega^{-1}\;m^{-1}$ (for *x* = 0.2), which is 5.6 times larger than that of TaIrTe_4_. Our results establish bulk mirror‐symmetry engineering as an effective strategy for designing energy‐efficient field‐free spin–orbit torque devices.

## Graphical abstract

Crystal orientation and domain engineering enable bulk mirror symmetry breaking in Bi_1−_ *_x_* Sb*_x_*, resulting in ultralow current‐induced switching of perpendicular magnetization without an external field. By exploiting single‐domain Bi_1−_ *_x_* Sb*_x_* (012), the authors demonstrate an out‐of‐plane spin Hall conductivity of $∼\;1.12\times 10^{5}\frac{\hbar }{2e}\Omega^{-1}\;m^{-1}$, significantly exceeding that of previously studied low‐symmetry materials. This finding establishes a new platform for energy‐efficient field‐free spin–orbit torque switching.

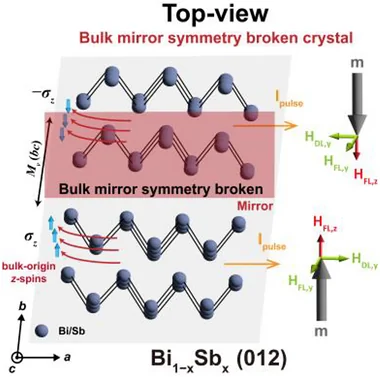

## 1 Introduction

Crystal symmetry and spin–orbit coupling (SOC) together dictate the generation and orientation of spin currents in solids [1, 2], defining the symmetry‐allowed components of the spin Hall effect and spin–orbit torque (SOT) [3, 4, 5, 6]. In systems possessing vertical mirror planes and two‐fold rotational (*C*_2_) symmetry, only in‐plane spins (***σ***_***y***_) are symmetry‐allowed, and thus the resulting SOTs cannot deterministically switch perpendicularly magnetized ferromagnets without an external magnetic field [7, 8, 9]. Therefore, realizing field‐free switching requires access to symmetry‐forbidden spin components, particularly out‐of‐plane spins (***σ***_***z***_), which emerge when specific crystal symmetries are broken [10, 11, 12, 13, 14, 15, 16].

Low‐symmetry materials such as WTe_2_ and TaIrTe_4_ have recently enabled ***σ***_***z***_‐induced SOTs by exploiting broken surface mirror symmetry [4, 5, 6, 17, 18, 19, 20, 21, 22]. However, their in‐plane spin Hall conductivity (σ_SH,*y*_) remains intrinsically small—the highest reported σ_SH,*y*_ value (TaIrTe_4_) [6] is only $∼\;0.55\times 10^{5}\frac{\hbar }{2e}\Omega^{-1}\;m^{-1}$, far below those of topological materials (TMs) [23, 24, 25, 26] and conventional heavy metals (HMs) [27, 28]. Moreover, the out‐of‐plane spin Hall conductivity (σ_SH,*z*_) is limited—the highest reported σ_SH,*z*_ value (TaIrTe_4_) [6] is only $∼\;0.21\times 10^{5}\frac{\hbar }{2e}\Omega^{-1}\;m^{-1}$. This limitation arises because the generation of ***σ***_***z***_ originates solely from surface carriers, leading to relatively high critical switching current densities (*J*_C_s) despite field‐free operation.

A promising strategy is to employ intrinsically bulk‐mirror‐symmetry‐broken materials with high spin Hall conductivity. When mirror symmetry is lifted throughout the bulk lattice rather than only at the surface, the spin–orbit–coupled band structure can generate ***σ***_***z***_ directly from bulk carriers, enabling large σ_SH,*z*_. Yet, no experimentally accessible bulk‐mirror‐symmetry‐broken spin sources have been demonstrated to provide deterministic field‐free SOT switching with energy efficiency suitable for practical devices.

Here, we demonstrate that epitaxial Bi_1−_ *_x_* Sb*_x_* (012) satisfies this requirement as a bulk‐mirror‐symmetry‐broken platform with simultaneously large σ_SH,*y*_ and σ_SH,*z*_. A critical challenge is that Bi_1−_ *_x_* Sb*_x_* (012) films typically form multiple in‐plane rotational variants on standard substrates, which suppresses symmetry‐driven ***σ***_***z***_ and prevents field‐free switching. In this work, we overcome this limitation by employing ferromagnetic Cr_1.6_Te_2_ with perpendicular magnetic anisotropy (PMA) as a buffer layer to engineer the in‐plane domain structure, yielding predominantly single‐domain Bi_1−_ *_x_* Sb*_x_* (012) for 0.1 ≤ *x* ≤ 0.3 and resulting in deterministic field‐free switching with an ultra‐low *J*_C_ of 8.15 × 10^5^ A/cm^2^ (for *x* = 0.2 at 150 K). Remarkably, field‐free switching persists even in anisotropic three‐fold domain regimes (0.4 ≤ *x* ≤ 0.8), supported by domain‐fraction‐dependent SOT analysis. Spin‐torque ferromagnetic resonance (ST‐FMR) at room temperature (RT) confirms the bulk origin of ***σ***_***z***_, revealing a σ_SH,*z*_ of $∼\;1.12\;\times 10^{5}\frac{\hbar }{2e}\Omega^{-1}m^{-1}$ (for *x* = 0.2)—one order of magnitude larger than that of typical low‐symmetry systems—while σ_SH,*y*_ exceeds $4.0\;\times 10^{5}\frac{\hbar }{2e}\Omega^{-1}\;m^{-1}$, far exceeding those of surface‐mirror‐symmetry‐broken systems, HMs, and TMs. These findings establish Bi_1−_ *_x_* Sb*_x_* (012) as the first bulk‐mirror‐symmetry‐broken material that simultaneously exhibits large in‐plane and out‐of‐plane spin Hall conductivities with deterministic field‐free switching. Moreover, our results present a scalable bulk symmetry‐engineering strategy that connects crystal symmetry principles to energy‐efficient spintronic technologies.

## 2 Bulk‐Mirror‐Symmetry‐Broken Bi_1−_ *_x_* Sb*_x_* (012)

The topological material Bi_1−_ *_x_* Sb*_x_* possesses distinct crystal symmetry depending on the surface orientation [26, 29, 30, 31]. As illustrated in Figure 1a, Bi_1−_ *_x_* Sb*_x_* (001) displays a hexagonal atomic configuration with three vertical mirror planes, while the (012) orientation shows a pseudocubic‐like configuration with a single vertical mirror plane along the *b*‐axis. The atomic lattice is additionally tilted out‐of‐plane about the *b*‐axis, and the central atom in the (012) unit cell exhibits unequal interatomic distances along the *b*‐axis [29], which breaks mirror symmetry along the *a*‐axis throughout the bulk lattice and thereby prohibits two‐fold rotational symmetry in Bi_1−_ *_x_* Sb*_x_* (012). From this crystallographic mirror‐symmetry breaking, two twin domains can be defined, denoted as α‐ and β‐domains depending on the tilt configuration (Figure 1a).

**FIGURE 1:**
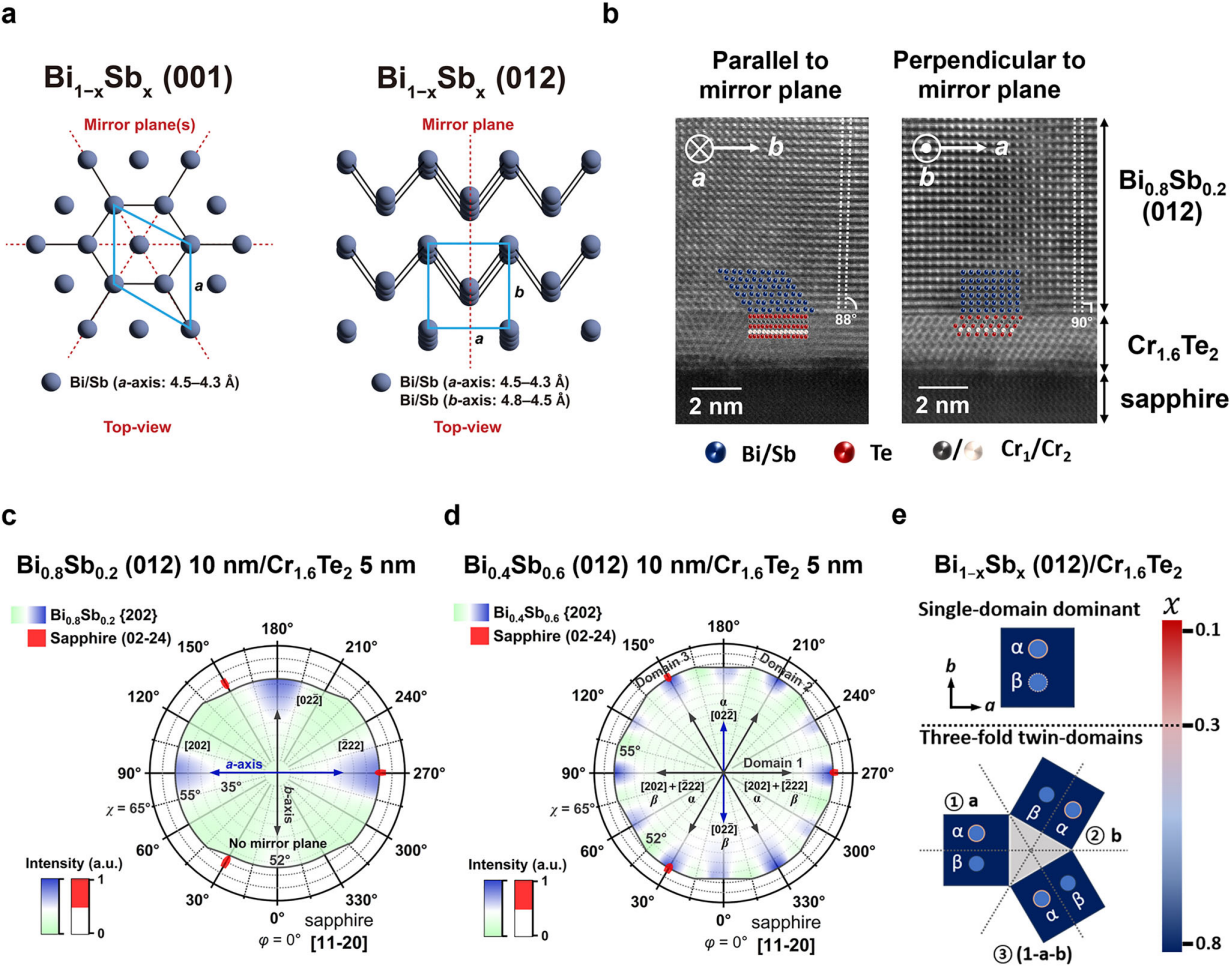
Sample characterizations. (a) Schematic illustration of the atomic structure of Bi_1−_ *_x_* Sb*_x_* along the (001) and (012) orientations (α‐domain) from a top‐view. The light‐blue solid line indicates the unit cell corresponding to each orientation. (b) Representative cross‐sectional HAADF‐STEM images of Bi_0.8_Sb_0.2_ (012)/Cr_1.6_Te_2_ along the *a*‐axis and *b*‐axis, respectively. XRD *φ*‐scan pole figures of (c) Bi_0.8_Sb_0.2_ (012) 10 nm/Cr_1.6_Te_2_ 5 nm, and (d) Bi_0.4_Sb_0.6_ (012) 10 nm/Cr_1.6_Te_2_ 5 nm. Bi_1−_ *_x_* Sb*_x_* {202} peaks and sapphire (02$\bar{2}$4) peaks were used for the pole figures, respectively. *φ* = 0° corresponds to sapphire [11$\bar{2}$0]. (e) Schematic illustration of the growth behavior and in‐plane domains of Bi_1−_ *_x_* Sb*_x_* (012) on Cr_1.6_Te_2_ buffer layer depending on Sb concentration. a, b, (1 − a − b) indicate the fractional populations of each domain, respectively.

Therefore, this crystallographic symmetry predicts the generation of ***σ***_***z***_ when the current is applied along the *a*‐axis of Bi_1−_ *_x_* Sb*_x_* (012). However, no experimentally accessible bulk‐mirror‐symmetry‐broken system has been realized to date, because Bi_1−_ *_x_* Sb*_x_* (012) grown on conventional substrates or RT–PMA ferromagnets (FMs) inevitably forms multiple rotational variants and α/β twin domains. These variants restore macroscopic mirror symmetry, thereby suppressing ***σ***_***z***_ generation and consequently preventing deterministic field‐free switching [32, 33]. To overcome this limitation, we employ a Cr_1.6_Te_2_ (001) buffer layer—a quasi‐2D PMA FM with a Curie temperature (*T*_C_) of 195 K [34]—which enables predominantly single‐domain Bi_1−_ *_x_* Sb*_x_* (012) films (0.1 ≤ *x* ≤ 0.3). Bi_1−_ *_x_* Sb*_x_* (012)/Cr_1.6_Te_2_ (Bi_1−_ *_x_* Sb*_x_*/Cr_1.6_Te_2_) heterostructures are grown by molecular beam epitaxy (MBE) on sapphire substrates, with growth mechanisms and characterization detailed in Supplementary Note S1 (Figures S1–S8 and Table S1).

Figure 1b displays representative cross‐sectional high‐angle annular dark‐field scanning transmission electron microscopy (HAADF‐STEM) images of Bi_0.8_Sb_0.2_/Cr_1.6_Te_2_ acquired along the sapphire [$\bar{1}$100] (left) and [11$\bar{2}$0] (right) zone axes. The pseudocubic stacking and atomically sharp interfaces are clearly resolved, and the expected lattice tilt (∼2°) is visible only in the left panel, confirming the assignment of the *a*‐ and *b*‐axes. The atomic structures of Cr_1.6_Te_2_ along sapphire [$\bar{1}$100] and [11$\bar{2}$0] are consistent with prior structural reports [34, 35].

The assignment of each axis of Bi_1−_ *_x_* Sb*_x_* (012) was independently confirmed by X‐ray diffraction (XRD) azimuthal angle (*φ*)‐scan measurements of the Bi_1−_ *_x_* Sb*_x_* {202} peaks [36, 37]. For a single‐domain Bi_1−_ *_x_* Sb*_x_* (012) film, exactly three Bi_1−_ *_x_* Sb*_x_* {202} peaks per 360° are expected at characteristic tilt angles *χ* ≈ 52° ((02$\bar{2}$)) and *χ* ≈ 55° ((202) and ($\bar{2}$22)) due to the absence of an in‐plane mirror counterpart of (02$\bar{2}$) arising from broken mirror symmetry along the *a*‐axis [36, 37]. As shown in the XRD pole figure (Figure 1c), Bi_0.8_Sb_0.2_/Cr_1.6_Te_2_ indeed presents three *φ* peaks without 120°‐separated replicas, evidencing the single‐domain (specifically α‐domain) nature of the films. Accordingly, the *a*‐axis (*b*‐axis) aligns with sapphire [$\bar{1}$100] ([11$\bar{2}$0]), which is consistent with the STEM data in Figure 1b. This single‐domain character persists across the 0.1 ≤ *x* ≤ 0.3 range, ensuring that the intrinsic bulk‐mirror‐symmetry breaking of Bi_1−_ *_x_* Sb*_x_* (012) directly manifests at the device scale (Figure S4). The detailed axis‐determination procedure is provided in Supplementary Note S1.3.

In contrast, Bi_1−_ *_x_* Sb*_x_*/Cr_1.6_Te_2_ with 0.4 ≤ *x* ≤ 0.8 exhibits twelve anisotropic *φ* peaks (Figure 1d; Figure S4): six weaker peaks at *χ* ≈ 52° and six stronger peaks at *χ* ≈ 55°, demonstrating their rotational variants separated by 120° with unequal domain populations and mixed α/β‐domain character [36, 37]. Figure 1e summarizes the domain evolution across composition *x*, identifying two distinct symmetry regimes: (i) single‐domain‐dominant bulk‐mirror‐symmetry‐broken phase for 0.1 ≤ *x* ≤ 0.3, and (ii) anisotropic three‐fold twin domain regime for 0.4 ≤ *x* ≤ 0.8.

## 3 Bulk‐Origin ***σ***_***z***_ Generation in Bi_1−_ *_x_* Sb*_x_* (012)

To probe current‐induced ***σ***_***z***_ generation in mirror‐symmetry‐broken Bi_1−_ *_x_* Sb*_x_* (012), ST‐FMR measurements were performed at RT on CoFeB (CFB)/Bi_1−_ *_x_* Sb*_x_*/Cr_1.6_Te_2_ heterostructures. As shown in Figure 2a, the devices were patterned into a coplanar‐waveguide (CPW) geometry, with the radio‐frequency current (*I*_RF_) applied along the *x*‐axis and the external in‐plane magnetic field (*H*_ext_) applied at 45° relative to *I*_RF_. The spin current generated by *I*_RF_ in Bi_1−_ *_x_* Sb*_x_* exerts oscillating SOTs on the magnetization *M* of CFB, resulting in its precession. Then, the anisotropic magnetoresistance of CFB modulates the device resistance at the same frequency as *I*_RF_, producing a mixing voltage *V_mix_* (DC component). The *V_mix_* signals consist of symmetric (*V*_S_) and antisymmetric (*V*_A_) components, which can be extracted by $V_{mix}=V_{S}+V_{A}=\tilde{V_{S}}\;F_{S}+\tilde{V_{A}}F_{A}$, where $\tilde{V_{S}}$ ($\tilde{V_{A}}$) is the amplitude of *V*_S_ (*V*_A_), and F_S_ (F_A_) is the symmetric (antisymmetric) Lorentzian function, respectively [38]. $\tilde{V_{S}}$ ($\tilde{V_{A}}$) is proportional to the *I*_RF_‐induced in‐plane torque (*τ*_∥_) and out‐of‐plane torque (*τ*_⊥_). The total SOT (***τ***_SOT_) can be represented as Equation (1) [4, 6, 17], where *τ*_DL,*y*_ (*τ*_DL,*z*_) is ***σ***_***y***_‐induced (***σ***_***z***_‐induced) damping‐like torque, *τ*_FL,*y*_ (*τ*_FL,*z*_) is ***σ***_***y***_‐induced (***σ***_***z***_‐induced) field‐like torque, and ***m*** is the magnetization direction of CFB. Here, the in‐plane torque is given by *τ*_∥_ = *τ*_DL,*y*_  + *τ*_FL,*z*_, and the out‐of‐plane torque by *τ*_⊥_ = *τ*_FL,*y*_  + *τ*_DL,*z*_ + *τ*_Oe_, where *τ*_Oe_ is the Oersted‐field‐induced torque:

(1) $$\begin{array}{ccc} \tau_{\mathrm{SOT}}= & & \tau_{\mathrm{DL},y}\cdot m\times \sigma_{y}\times m-\tau_{\mathrm{FL},y}\cdot m\times \sigma_{y}+\\ & & \tau_{\mathrm{DL},z}\cdot m\times \sigma_{z}\times m-\tau_{\mathrm{FL},z}\cdot m\times \sigma_{z} \end{array}$$

**FIGURE 2:**
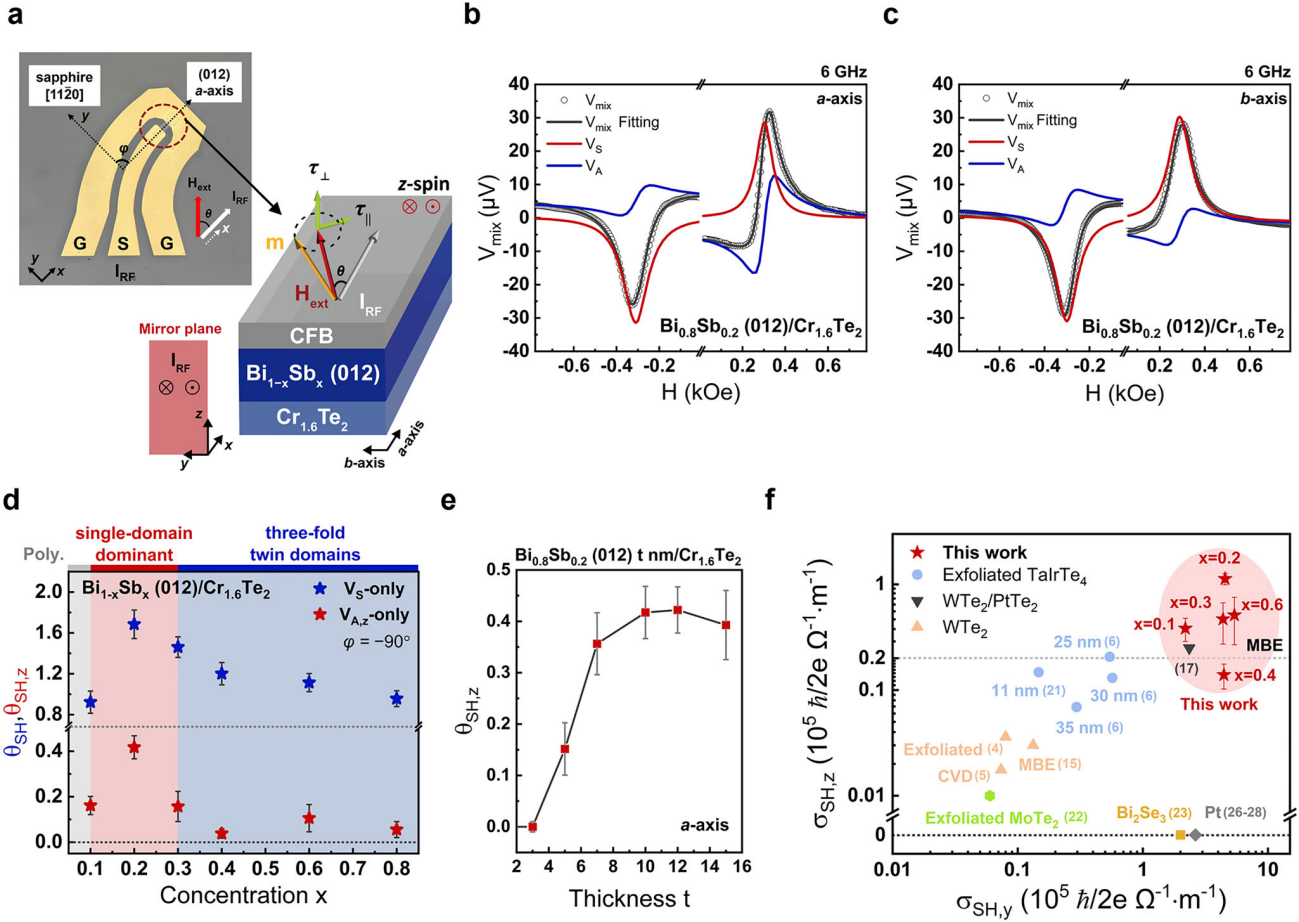
ST‐FMR measurement data. (a) (Left panel) Optical image of the ground–signal–ground (GSG) type CPW device used in this study, illustrating the device geometry. *φ* denotes the relative angle of the CPW channel with respect to the sapphire [11$\bar{2}$0]. *H*_ext_ is applied at an angle *θ* relative to *I*_RF_. (Right panel) Schematic illustration of an enlarged CPW channel. Green arrows represent the torques, where *τ*_∥_ is the in‐plane torque, and *τ*_⊥_ is the out‐of‐plane torque. The magnetization *M* (yellow arrow) precesses around *H*_ext_. Representative *V_mix_* signals measured for (b) *φ* = −90° (*a*‐axis), and (c) *φ* = 0° (*b*‐axis) in a CFB 5 nm/Bi_0.8_Sb_0.2_ (012) 10 nm/Cr_1.6_Te_2_ 5 nm heterostructure at 6 GHz and 20 dBm *I*_RF_. Solid lines are the fitting results corresponding to *V_mix_*, *V*_S_, and *V*_A_. (d) *θ*_SH_ and *θ*_SH,*z*_ of Bi_1−_ *_x_* Sb*_x_* (012)/Cr_1.6_Te_2_ (*φ* = −90°) as a function of *x*, extracted using the *V*_S_‐only and *V*_A,z_‐only methods. The *V_mix_* data measured at 6 GHz were used. (e) *θ*_SH,*z*_ of Bi_0.8_Sb_0.2_ (012) *t* nm/Cr_1.6_Te_2_ 5 nm with *t*  = 3, 5, 7, 10, 12, and 15. The error bars represent the uncertainty associated with extracting *θ*_SH,*z*_ values. (f) Summary of σ_SH,*y*_ and σ_SH,*z*_ values from previous reports. The red stars in the red‐shaded area represent the σ_SH_ values obtained in this work. Numbers in parentheses denote reference numbers. The error bars represent the standard deviation derived from multiple devices.

In conventional SOT systems, where mirror symmetry and *C*_2_ symmetry are preserved, τ_DL,*z*_ is forbidden. Consequently, only ***σ***_***y***_‐induced SOTs are generated (***τ***_SOT_ = *τ*_DL,*y*_· ***m*** × ***σ***_***y***_× ***m*** −*τ*_FL,*y*_· ***m*** × ***σ***_***y***_), which leads to *V_mix_* (***m***) = −*V_mix_* (−***m***). Specific current‐induced SOT is generated when the sign of the torque changes with the sign of the current under symmetry operations. In the ***σ***_***z***_‐allowed system (Equation 1), the amplitude and shape of *V_mix_* (***m***) differ from those of −*V_mix_* (−***m***), indicating that the system lacks mirror and *C*_2_ symmetry [4, 5, 6, 17, 22]. As shown in Figure 2b,c, the representative *V_mix_* signals of CFB/Bi_0.8_Sb_0.2_/Cr_1.6_Te_2_ satisfy $V_{mix}\left(m\right)\neq -V_{mix}\left(-m\right)$, with a clear difference in $\tilde{V_{A}}\left(m\right)$ and $\tilde{V_{A}}\left(-m\right)$ only when *I*_RF_ is applied along the *a*‐axis, whereas the shape and amplitude of *V_mix_* do not change with opposite ***m*** along the *b*‐axis. This indicates that the ***σ***_***z***_ generation is intrinsically tied to the crystal symmetry, as expected from Figure 1. Symmetry analysis of ***τ***_SOT_ is detailed in Supplementary Note S2.

Following the established analysis method (Supplementary Note S3.1) [4, 6, 17], in‐plane and out‐of‐plane effective spin Hall angles (*θ*_SH_ and *θ*_SH,*z*_, respectively) are extracted for Bi_1−_ *_x_* Sb*_x_*/Cr_1.6_Te_2_ (0.1 ≤ *x* ≤ 0.3, *a*‐axis), yielding *θ*_SH_ = 1.68 and *θ*_SH,*z*_ = 0.417 for Bi_0.8_Sb_0.2_/Cr_1.6_Te_2_ (Figure 2d). Unexpectedly, even three‐fold twin domain samples (0.4 ≤ x ≤ 0.8) show finite *θ*_SH,*z*_, indicating that ***σ***_***z***_ persists despite multiple domain variants, consistent with the domain‐imbalance‐induced out‐of‐plane SOT revealed later (Figure 4). Complete *V_mix_* data for Bi_1−_ *_x_* Sb*_x_*/Cr_1.6_Te_2_ are provided in Figures S9–S15.

For the single‐domain Bi_1−_ *_x_* Sb*_x_*/Cr_1.6_Te_2_, the extracted *θ*_SH,*z*_ exceeds that of typical low‐symmetry materials, suggesting that ***σ***_***z***_ originates from bulk carriers rather than surface carriers. To confirm the bulk origin of ***σ***_***z***_, we examined the thickness dependence of *θ*_SH,*z*_ in single‐domain Bi_0.8_Sb_0.2_ (*t* nm)/Cr_1.6_Te_2_, which rapidly increases for *t* > 3 nm and saturates around t∼ 10 nm (Figure 2e), demonstrating that the magnitude of the ***σ***_***z***_‐polarized spin current increases with thickness—the opposite trend of surface‐mirror‐symmetry‐broken systems, where ***σ***_***z***_ decreases with increasing thickness [6, 19]. This confirms that ***σ***_***z***_ originates from bulk carriers, enabled only when mirror symmetry is broken throughout the bulk lattice.

The spin Hall conductivity (σ_SH_ = *θ*_SH_σ_C_) of ***σ***_***y***_ (σ_SH,*y*_) and ***σ***_***z***_ (σ_SH,*z*_) in Bi_1−_ *_x_* Sb*_x_*/Cr_1.6_Te_2_ was estimated using measured charge conductivity (σ_C_) values (Figure S16 and Table S2). For example, the best values are $\sigma_{\mathrm{SH},y}=4.55\times 10^{5}\frac{\hbar }{2e}\Omega^{-1}\;m^{-1}$ and $\sigma_{\mathrm{SH},z}=1.12\times 10^{5}\frac{\hbar }{2e}\Omega^{-1}\;m^{-1}$ for Bi_0.8_Sb_0.2_/Cr_1.6_Te_2_. Figure 2f compares σ_SH,*y*_ and σ_SH,*z*_ of Bi_1−_ *_x_* Sb*_x_*/Cr_1.6_Te_2_ with conventional spin Hall materials and other low‐symmetry (surface‐mirror‐symmetry‐broken) materials. The σ_SH,*y*_ of Bi_1−_ *_x_* Sb*_x_*/Cr_1.6_Te_2_ (red‐shaded area) is 1–2 orders of magnitude larger than that of low‐symmetry materials [4, 5, 6, 17, 21, 22] and 1.8 times larger than that of Pt [26, 27, 28] (which hosts only σ_SH,*y*_). The σ_SH,*z*_ of Bi_0.8_Sb_0.2_/Cr_1.6_Te_2_ (bulk‐mirror‐symmetry‐broken) is approximately five times larger than that of TaIrTe_4_ [6] and WTe_2_/PtTe_2_ (spin‐swapping‐induced ***σ***_***z***_) [17], which exhibit σ_SH,*z*_ values of $0.21\times 10^{5}\frac{\hbar }{2e}\Omega^{-1}\;m^{-1}$ and $0.25\times 10^{5}\frac{\hbar }{2e}\Omega^{-1}\;m^{-1}$, respectively. Thus, a central challenge for efficient field‐free switching in typical low‐symmetry materials—simultaneously achieving large σ_SH,*z*_ and σ_SH,*y*_—is resolved in bulk‐mirror‐symmetry‐broken Bi_0.8_Sb_0.2_/Cr_1.6_Te_2_.

## 4 Deterministic Field‐Free Switching Enabled by Bulk‐Origin ***σ***_***z***_ in Bi_1−_ *_x_* Sb*_x_* (012)/Cr_1.6_Te_2_

We performed perpendicular magnetization switching measurements by applying current in single‐domain Bi_0.8_Sb_0.2_ (012) 10 nm/Cr_1.6_Te_2_ 5 nm (Bi_0.8_Sb_0.2_/Cr_1.6_Te_2_) heterostructures. As shown in Figure 3a, since Bi_0.8_Sb_0.2_ (012) has a single vertical mirror plane along the *b*‐axis (*M_v_* (*bc*)) without *C*_2_ symmetry, the generation of ***σ***_***z***_ is allowed for currents applied along the *a*‐axis. As shown in Figure 3b, the generated ***σ***_***z***_ as well as ***σ***_***y***_ exert SOTs on the magnetization of adjacent Cr_1.6_Te_2_; ***τ***_SOT_ follows the same symmetry as Equation (1), with ***m*** replaced by that of Cr_1.6_Te_2_. Hall bar devices were fabricated by aligning the device geometry with the crystallographic axes of Bi_0.8_Sb_0.2_ (Figure 3c). To verify PMA, we applied a 0.1 mA DC current (*I*_DC_) along the *a*‐axis and measured the Hall voltage *V_y_* while sweeping an out‐of‐plane magnetic field (*B*‐field). The Hall resistance (*R_xy_*) was calculated as *R_xy_* = *V_y_*/*I_DC_*. A clear anomalous Hall effect (AHE) loop at 150 K (Figure 3d) confirms PMA in Bi_0.8_Sb_0.2_/Cr_1.6_Te_2_ with an AHE resistance of 1.06 Ω, supported by out‐of‐plane magnetization versus *B*‐field measurements (inset of Figure 3d). Because the magnetization of Bi_0.8_Sb_0.2_/Cr_1.6_Te_2_ rapidly decreases above ∼150 K (Figure S18), the AHE loop becomes difficult to detect above ∼180 K; therefore, current‐induced magnetization switching measurements were performed at 150 K for enhanced signal visibility.

**FIGURE 3:**
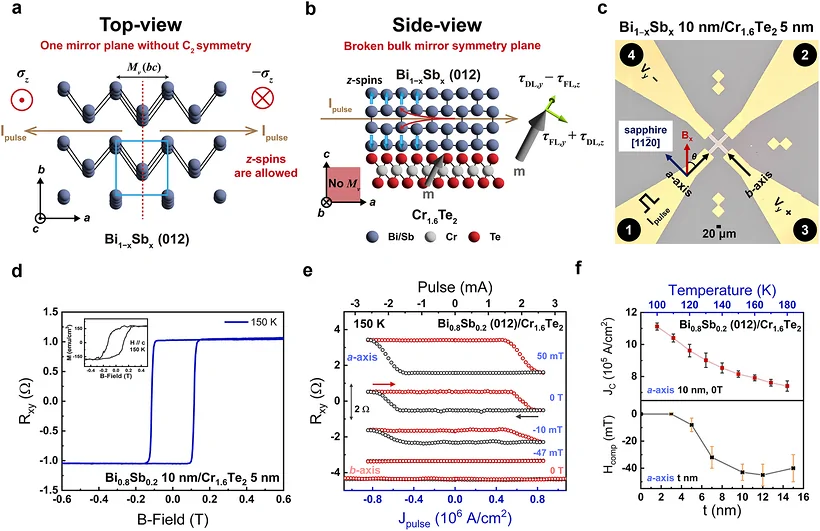
Field‐free SOT switching of bulk‐mirror‐symmetry‐broken single‐domain Bi_0.8_Sb_0.2_ (012) 10 nm/Cr_1.6_Te_2_ 5 nm. Schematic illustrations of (a) ***σ***_*z*_ generation, and (b) SOT generation for a current applied along the *a*‐axis with the broken bulk mirror plane in Bi_0.8_Sb_0.2_ (012). The gray arrow and green arrow indicate the magnetization of Cr_1.6_Te_2_ and the SOTs, respectively. (c) Optical image of the Hall bar devices with four electrodes used in this study. Each electrode is numbered from 1 to 4. *θ* is the in‐plane relative angle between the ***B***_***x***_ (red arrow) and the *a*‐axis. The blue arrow indicates the sapphire [11$\bar{2}$0]. (d) AHE loop of Bi_0.8_Sb_0.2_ (012) 10 nm/Cr_1.6_Te_2_ 5 nm at 150 K. *R_xy_* denotes the Hall resistance. Inset: Magnetization versus out‐of‐plane *B*‐field curve of Bi_0.8_Sb_0.2_ (012) 10 nm/Cr_1.6_Te_2_ 5 nm at 150 K. (e) Current‐induced magnetization switching under different in‐plane *B*‐fields and without *B*‐field in Bi_0.8_Sb_0.2_ (012) 10 nm/Cr_1.6_Te_2_ 5 nm along the *a*‐ and *b*‐axes. The loops are manually shifted vertically for clarity. (f) (Top panel) Field‐free *J*_C_ as a function of temperature for Bi_0.8_Sb_0.2_ (012) 10 nm/Cr_1.6_Te_2_ 5 nm (*a*‐axis). (Bottom panel) *H*_comp_ as a function of *t* in Bi_0.8_Sb_0.2_ (012) *t* nm/Cr_1.6_Te_2_ 5 nm (*t*  = 3, 5, 7, 10, 12, and 15) with current along the *a*‐axis. Error bars represent the standard deviation from multiple measurements.

We demonstrated SOT‐induced magnetization switching using 100 µs current pulses (*I*_pulse_) swept from −3 to 3 mA at 150 K (Figure 3e). Deterministic switching of the PMA Cr_1.6_Te_2_ layer is achieved without an external field, with a remarkably low critical switching current density *J*_C_ of 8.15 × 10^5^ A/cm^2^ along the *a*‐axis, which is one of the lowest values reported for field‐free SOT devices [6, 20, 21]. Details of the *J*_C_ calculation using the parallel‐resistor model are provided in Supplementary Note S4.1. The switching polarity under zero *B*‐field indicates that the −*z* direction of ***σ***_***z***_ is injected into the Cr_1.6_Te_2_ bottom layer, acting as an effective symmetry‐breaking field [6].

In conventional SOT systems, where only ***σ***_***y***_‐induced SOT occurs, an in‐plane *B*‐field (***B***_***x***_) is required for deterministic switching; ***B***_***x***_ breaks the switching symmetry, which acts as an effective field (∝ ***B***_***x***_ × ***σ***_***y***_). For example, in the bottom FM SOT structure, the +*y* direction of ***σ***_***y***_ with ***B***_***x***_ opposite to the current direction induces an effective field along the −*z* direction; the magnetization switches from up to down. Therefore, the switching polarity is reversed depending on the ***B***_***x***_ direction. We also measured magnetization switching of Bi_0.8_Sb_0.2_/Cr_1.6_Te_2_ under ***B***_***x***_ applied along the *a*‐axis. However, the switching polarity does not change under opposite polarity of ***B***_***x***_ (Figure 3e), which is not expected for conventional SOT devices [7, 8]. Moreover, the AHE loop disappears when 47 mT of ***B***_***x***_ is applied; we denote this field as the compensation field (*H*_comp_). This occurs because the effective field generated by ***σ***_***z***_ is canceled by that of ***B***_***x***_ [6, 20]. Furthermore, *H*_comp_ is proportional to the magnitude of the generated ***σ***_***z***_. Therefore, the switching polarity is dominated by the ***σ***_***z***_ direction when | ***B***_***x***_| < *H*_comp_. Supplementary Note S4.2 provides a switching polarity change under opposite ***B***_***x***_ in the *b*‐axis, where ***σ***_***z***_ is absent.

To further verify the presence of ***σ***_***z***_‐induced SOT in the actual switching structure, we performed second‐harmonic Hall voltage (SH‐HV) measurements on Bi_0.8_Sb_0.2_/Cr_1.6_Te_2_ at 150 K (Figure S17). This additional measurement was conducted because the ST‐FMR measurements were performed at RT using a CFB/Bi_1−_ *_x_* Sb*_x_* interface, whereas the current‐induced switching was demonstrated at the Bi_0.8_Sb_0.2_/Cr_1.6_Te_2_ interface at 150 K. The SH‐HV results show a finite *θ*_SH,z_ of 0.273 for the *a*‐axis, while it is negligible for the *b*‐axis. Details of *θ*_SH,z_ in Bi_0.8_Sb_0.2_/Cr_1.6_Te_2_ at 150 K are provided in Supplementary Note S3.2.

To further confirm that ***σ***_***z***_ is generated by bulk carriers in Bi_0.8_Sb_0.2_/Cr_1.6_Te_2_, we extracted *H*_comp_ as a function of Bi_0.8_Sb_0.2_ thickness (*t*) for current applied along the *a*‐axis. If bulk carriers with ***σ***_***z***_ are generated, *H*_comp_ should increase with thickness until the spin diffusion length of ***σ***_***z***_ is reached and then saturate. As shown in Figure 3f (bottom panel), *H*_comp_ increases sharply from *t*  = 3 nm to *t*  = 10 nm and then saturates—a bulk‐dominance signature of ***σ***_***z***_, in excellent agreement with the thickness dependence of *θ*_SH,*z*_ in Figure 2e. The suppressed generation of ***σ***_***z***_ for *t* < 3 nm is attributed to interfacial strain at the Bi_0.8_Sb_0.2_/Cr_1.6_Te_2_ interface (Supplementary Note S1.2).

We also observed persistent field‐free switching over 100–180 K, as shown in Figure 3f (top panel); *J*_C_ increases as temperature decreases. We also successfully grew Bi_0.8_Sb_0.2_ (012) on an RT PMA FM Co/Pt superlattice ([Co/Pt]), where no in‐plane ordering is formed and thus no macroscopic ***σ***_***z***_ is expected, and investigated magnetization switching at RT (Figure S23). As expected, only magnetization switching under ***B***_***x***_ was observed in Bi_0.8_Sb_0.2_ (012) 10 nm/[Co/Pt] 4 nm with a *J*_C_ of 2.47 × 10^6^ A/cm^2^, validating that ***σ***_***z***_ is absent and deterministic field‐free switching is not obtained when Bi_1−_ *_x_* Sb*_x_* (012) lacks in‐plane ordering. Overall, field‐free SOT switching results in Figure 3 and Figures S19–S22 for other single‐domain Bi_1−_ *_x_* Sb*_x_*/Cr_1.6_Te_2_ demonstrate that bulk‐mirror‐symmetry breaking in Bi_1−_ *_x_* Sb*_x_* (012) enables intrinsically low‐power field‐free switching that has not been achievable in conventional SOT systems.

## 5 Symmetry‐Governed Field‐Free Switching by Anisotropic Three‐Fold Domain Bi_1−_ *_x_* Sb*_x_* (012)

Having established the bulk‐origin ***σ***_***z***_‐induced field‐free switching in single‐domain films (Figure 3), we next investigated whether symmetry‐governed field‐free switching can also be realized in Bi_1−_ *_x_* Sb*_x_*/Cr_1.6_Te_2_ films (0.4 ≤ *x* ≤ 0.8), where Bi_1−_ *_x_* Sb*_x_* (012) results in anisotropic three‐fold twin domains (#1–#3), each consisting of α‐ and β‐domains with broken mirror symmetry. As shown in Figure 4a, when a current is applied at an angle *θ* relative to the *a*‐axis of the #1 α‐domain, the projection of the applied current onto the *a*‐axes of all domains (*I*_*a*, *multi*_) can be represented as *I*_*a*, *multi*_ = a(α_1_ − β_1_)cosθ − b(α_2_ − β_2_)cos(60° − θ) − (1 − a − b)(α_3_ − β_3_)sin(θ − 30°), where α_*i*_ and β_*i*_ denote the fractional populations of the α/β domains within domains #1–#3, respectively; $\alpha_{i}-\beta_{i}\neq 0$, as confirmed by the anisotropic *φ* peaks in the XRD pole figure (Figure 1d). Taking α_*i*_ − β_*i*_ = 1 for convenience, *I*_*a*, *multi*_ is nonzero except at a = b = 1/3, where exact cancellation among all domains makes *I*_*a*, *multi*_ = 0. A nonzero *I*_*a*, *multi*_ indicates that ***σ***_***z***_ can be generated.

**FIGURE 4:**
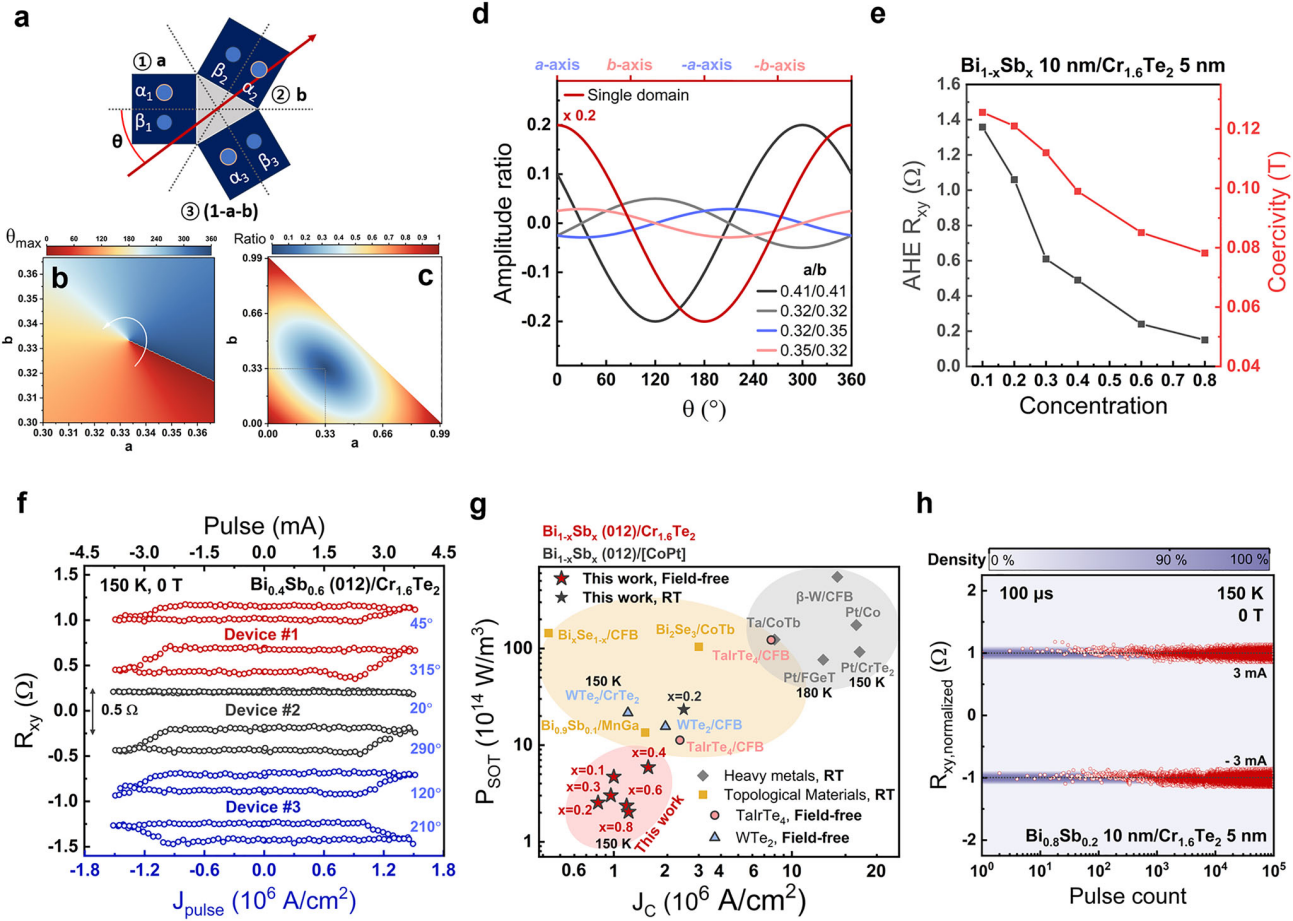
Field‐free SOT switching of bulk‐mirror‐symmetry‐broken Bi_1−_ *_x_* Sb*_x_* (012) with anisotropic three‐fold twin domains. (a) Schematic illustration of the current application in three‐fold twin domains (#1–#3) of Bi_1−_ *_x_* Sb*_x_* (012)/Cr_1.6_Te_2_. The domains occupy fractional populations a, b, and (1 − a − b), respectively. *θ* is the in‐plane angle between the applied current and the *a*‐axis of domain #1. (b) Calculated *θ*_*max*_ as a function of domain fractions that maximize *I*_*a*, *multi*_. (c) *I*_*a*, *multi*_/*I*_*a*, *single*_ ratio at *θ*_*max*_ for three‐fold twin domains. (d) *θ* dependence of *I*_*a*, *multi*_ for different domain‐fraction sets. The single‐domain case is shown as the red solid line. (e) AHE resistance and coercivity of Bi_1−_ *_x_* Sb*_x_* (012) 10 nm/Cr_1.6_Te_2_ 5 nm at 150 K. (f) Field‐free SOT switching in multiple Bi_0.4_Sb_0.6_ (012) 10 nm/Cr_1.6_Te_2_ 5 nm devices with current applied at specific angles relative to sapphire [11$\bar{2}$0]. The loops are manually shifted vertically for clarity. (g) Summary of *J*_C_ and *P*_SOT_ from previous studies. The red stars with gray solid borders in the red‐shaded area (gray star) represent *J*_C_ and *P*_SOT_ of our work for Bi_1−_ *_x_* Sb*_x_* (012) 10 nm/Cr_1.6_Te_2_ 5 nm at 150 K (Bi_0.8_Sb_0.2_ (012) 10 nm/[Co/Pt] 4 nm at RT). (h) Field‐free SOT switching endurance of Bi_0.8_Sb_0.2_ (012) 10 nm/Cr_1.6_Te_2_ 5 nm at 150 K under ± 3 mA, 100 µs *I*_pulse_. *R*_*xy*, normalized_ denotes *R_xy_* normalized by the SOT‐induced saturated *R_xy_*. The color map indicates the statistical density of the *R*_*xy*, normalized_ values, showing their distribution over repeated switching cycles.

The calculated angle *θ*_*max*_ that maximizes *I*_*a*, *multi*_ for different domain fractions (Figure 4b) shows that *θ*_*max*_ always exists. For a single‐domain case, the *a*‐axis projection is *I*_*a*, *single*_ = cos*θ*, with *θ*_*max*_ = 0°. Using these, we evaluated the normalized ***σ***_***z***_‐induced SOT‐driving current *I*_*a*, *multi*_/*I*_*a*, *single*_ at *θ*_*max*_ versus domain fractions (Figure 4c). Remarkably, even a modest 10% deviation from the equal‐domain fraction produces $\frac{I_{a,multi}}{I_{a,single}}\approx 0.1$, a sufficient ***σ***_***z***_ generation comparable to that of surface carriers responsible for ***σ***_***z***_ in low‐symmetry materials such as WTe_2_ and TaIrTe_4_ [4, 5, 6, 18, 19, 20, 21]. In thin films grown by the physical vapor deposition (PVD) method, a ∼10% deviation in the local domain fraction over an arbitrary area is plausible. Importantly, because the *θ* dependence of *I*_*a*, *multi*_ has the same 2π periodicity as *I*_*a*, *single*_, applying current at *θ*_*max*_ ± 90° yields *I*_*a*, *multi*_ = 0 (***σ***_***z***_‐disabled), demonstrating single‐domain‐like functionality (Figure 4d). Collectively, even in the presence of anisotropic three‐fold twin domains, bulk‐mirror‐symmetry‐broken Bi_1−_ *_x_* Sb*_x_*/Cr_1.6_Te_2_ can operate as an effective field‐free SOT source by leveraging a nonvanishing portion of bulk carriers with ***σ***_***z***_. Details of *I*_*a*, *multi*_ and its *θ* dependence are provided in Supplementary Note S5.1 (Figure S24).

To further confirm these calculations, we investigated current‐induced magnetization switching characteristics in Bi_1−_ *_x_* Sb*_x_*/Cr_1.6_Te_2_ (0.4 ≤ *x* ≤ 0.8). A clear AHE loop of Bi_1−_ *_x_* Sb*_x_*/Cr_1.6_Te_2_ (0.4 ≤ *x* ≤ 0.8) with coercivity at 150 K confirms PMA (Figures S25–S28), and the AHE resistance and coercivity for all compositions are presented in Figure 4e. The AHE resistance decreases with increasing *x* due to the reduced resistivity of Bi_1−_ *_x_* Sb*_x_* at higher *x* values (Supplementary Note S1.6). The increase in coercivity as the Bi concentration increases is due to an interfacial proximity effect induced by the strong SOC of Bi [26, 39]. However, the effective anisotropy field (*H*_*k*, eff_) and saturation magnetization (*M*_S_) of Bi_1−_ *_x_* Sb*_x_*/Cr_1.6_Te_2_ did not change significantly as a function of Sb concentration (Figure S18). Importantly, deterministic field‐free switching was consistently observed across all Bi_1−_ *_x_* Sb*_x_*/Cr_1.6_Te_2_ devices with 0.4 ≤ *x* ≤ 0.8 (Figures S25–S28) using Hall bar devices with channels oriented at various *θ* relative to the sapphire [11$\bar{2}$0]. Moreover, all Bi_1−_ *_x_* Sb*_x_*/Cr_1.6_Te_2_ (0.4 ≤ *x* ≤ 0.8) devices show *H*_comp_ in the magnetization switching measurements under ***B***_***x***_ (Figures S25–S28). For three representative Bi_0.4_Sb_0.6_/Cr_1.6_Te_2_ devices (Figure 4f), with current applied along arbitrary directions, field‐free switching with different switching polarities was observed, in excellent agreement with the calculation in Figure 4d. For example, when current is applied along two orthogonal *θ* directions, ***σ***_***z***_ can be generated with identical or opposite polarity, or only at one of the two angles (corresponding to *θ*_*max*_ and *θ*_*max*_ ± 90°). For example, Bi_0.4_Sb_0.6_/Cr_1.6_Te_2_ Device #2 shows field‐free switching only at *θ* = 290°, with the switching polarity inverted relative to the single‐domain case (current along the *a*‐axis), whereas no switching occurs at *θ* = 20°. This agrees with the condition where *θ*_*max*_ + 180° aligns with 290°.

We also estimated thermal effects induced by pulsed current during switching measurements, as described in Supplementary Note S6 (Figure S29 and Table S3), indicating that thermal effects do not play a dominant role in field‐free magnetization switching. Taken together, these distinctly angle‐dependent behaviors conclusively demonstrate that even anisotropic multifold‐domain Bi_1−_ *_x_* Sb*_x_* (012) films generate a finite bulk ***σ***_***z***_‐induced SOT, enabling deterministic field‐free magnetization switching. Furthermore, if the ***σ***_***z***_ generation were not bulk‐originated in Bi_1−_ *_x_* Sb*_x_*/Cr_1.6_Te_2_, field‐free switching would not occur for 0.4 ≤ *x* ≤ 0.8 due to the small ratio of $\frac{I_{a,multi}}{I_{a,single}}$.

We further benchmarked the *J*_C_ and the power density of SOT switching (*P*_SOT_) in Bi_1−_ *_x_* Sb*_x_*/Cr_1.6_Te_2_ and [Co/Pt]_4_ against other SOT systems in Figure 4g and Table S4. *P*_SOT_ is calculated as *P*_SOT_ = *ρ*_C_(*J*_C_)^2^, where *ρ*_C_ is the resistivity of the SOT source (Supplementary Note S1.6). The field‐free *J*_C_ and *P*_SOT_ of Bi_1−_ *_x_* Sb*_x_*/Cr_1.6_Te_2_ (red‐shaded area) lie one order lower than those of field‐free SOT systems such as WTe_2_ [20, 21] and TaIrTe_4_ [6, 21], and two orders lower than those of conventional HM‐based SOT systems requiring an assist field ***B***_***x***_ [40, 41, 42]. Compared to Bi_1−_ *_x_* Sb*_x_* (012) systems with no in‐plane ordering and no ***σ***_***z***_ (yellow‐shaded area), such as Bi_0.9_Sb_0.1_/MnGa [32] and Bi_0.8_Sb_0.2_/[Co/Pt] (gray star, this work), single‐domain Bi_1−_ *_x_* Sb*_x_*/Cr_1.6_Te_2_ shows one order smaller *J*_C_ and *P*_SOT_, which is attributed to strong bulk‐originated ***σ***_***z***_ generation. We also added a comparison using *J*_C_/μ_0_ *M*_S_ *H*_*k*, eff_ as a figure of merit to Supplementary Note S7 (Table S5) to provide a more direct comparison of the SOT switching performance across different Bi_1−_ *_x_* Sb*_x_* compositions. Since $\frac{1}{2}\mu_{0}M_{S}H_{k,\mathrm{eff}}$ represents the effective anisotropy energy density scale and the thickness of Cr_1.6_Te_2_ was fixed for all samples, this normalization reduces the influence of magnetic hardness on the variation in *J*_C_ [43]. Consequently, further studies to realize single‐domain Bi_1−_ *_x_* Sb*_x_* (012) with high σ_C_ (*x* ≥ 0.4) are essential to reduce *P*_SOT_ in field‐free SOT systems.

Finally, endurance testing using repetitive ± 3 mA *I*_pulse_ applied along the *a*‐axis of Bi_0.8_Sb_0.2_ (012)/Cr_1.6_Te_2_ demonstrates robust and fatigue‐free field‐free switching for over 10^5^ cycles (Figure 4h), highlighting outstanding operational stability and the potential for practical applications of this bulk‐mirror‐symmetry‐broken SOT platform. This comprehensive analysis reveals the robust scalability of bulk‐symmetry‐breaking‐driven SOTs across a broad range of compositions, underscoring the effectiveness of domain‐engineering strategies for practical device implementation. The composition‐controlled evolution of ***σ***_***z***_ generation and the resulting deterministic field‐free switching firmly establish Bi_1−_ *_x_* Sb*_x_* (012) as a tunable bulk spin source for energy‐efficient SOT technologies.

## 6 Conclusion

We demonstrated that intrinsic bulk‐mirror‐symmetry breaking in epitaxial Bi_1−_ *_x_* Sb*_x_* (012) with high in‐plane spin Hall conductivity generates a large ***σ***_***z***_ component, enabling highly efficient deterministic field‐free switching. By employing a symmetry‐guided Cr_1.6_Te_2_ buffer layer, we achieved robust macroscopic bulk‐mirror‐symmetry breaking across a wide composition range of Bi_1−_ *_x_* Sb*_x_*, confirming that functional ***σ***_***z***_‐driven torques can be generated even in multi‐domain structures. Spin‐torque ferromagnetic resonance and thickness‐dependent analyses further reveal the bulk origin of ***σ***_***z***_ in Bi_1−_ *_x_* Sb*_x_* (012), yielding a giant out‐of‐plane spin Hall conductivity of $∼1.12\times 10^{5}\frac{\hbar }{2e}\Omega^{-1}\;m^{-1}$ (for *x* = 0.2) and enabling deterministic field‐free switching at an exceptionally low critical switching current density of 8.15 × 10^5^ A/cm^2^. These results establish Bi_1−_ *_x_* Sb*_x_* (012) as a single‐material platform capable of simultaneously providing strong ***σ***_***z***_‐induced torque and high in‐plane spin Hall conductivity. More broadly, our symmetry‐design framework expands the materials‐design space for energy‐efficient SOT technologies and offers a scalable route toward practical field‐free spintronic applications.

## 7 Methods

### 7.1 Sample Characterization

Bi_1−_ *_x_* Sb*_x_* (012) and Cr_1.6_Te_2_ (001) were grown on c‐plane sapphire (0001) 750 µm substrate using MBE system equipped with Knudsen cells at a base pressure below 3 × 10^−9^ Torr. Prior to deposition, the sapphire substrates were sequentially annealed at 300°C for 30 min and 600°C for 1 h to remove surface impurities. For Cr_1.6_Te_2_ (001) growth, the sapphire substrate was maintained at 360°C, and Knudsen effusion cell temperatures for Cr and Te were controlled during evaporation. The evaporation flux ratio of Cr to Te was adjusted to approximately 1:6.03. Single‐phase Bi_1−_ *_x_* Sb*_x_* (012) was grown above the Cr_1.6_Te_2_ (001) buffer layer. Even an ultrathin (∼2 nm) Cr_1.6_Te_2_ buffer layer enabled the growth of single‐phase Bi_1−_ *_x_* Sb*_x_* (012). For magnetization switching measurements, a 2 nm SiO_2_ capping layer was deposited on Bi_1−_ *_x_* Sb*_x_* (012)/Cr_1.6_Te_2_ via the sputtering method to prevent oxidation of the Bi_1−_ *_x_* Sb*_x_* (012) layer. For ST‐FMR measurements, a 2 nm SiO_2_ capping layer was deposited on CFB/Bi_1−_ *_x_* Sb*_x_* (012)/Cr_1.6_Te_2_ heterostructures using the sputtering method to prevent oxidation of the CFB layer. A CFB 5 nm and a SiO_2_ 2 nm capping layer were deposited using the sputtering method in a vacuum chamber with a base pressure of 4 × 10^−9^ Torr, and all steps were performed in situ without air exposure.

XRD measurements were conducted to examine the crystallographic orientation and crystallinity of the films. In *θ* − 2*θ* XRD scans (Supplementary Note S1), Bi_1−_ *_x_* Sb*_x_* (012)/Cr_1.6_Te_2_ used in this study exhibits only the (012) family peaks. The Sb concentration of Bi_1−_ *_x_* Sb*_x_* was verified using in situ X‐ray photoelectron spectroscopy. The stoichiometry of Cr_1.6_Te_2_ was analyzed by a field‐emission scanning electron microscope operated at an accelerating voltage of 15 kV.

### 7.2 Transmission Electron Microscopy

Samples for cross‐sectional HAADF‐STEM were fabricated using a focused ion beam (FIB) system (HITACHI Ethos NX5000). FEI Double Cs‐corrected Titan Themis transmission electron microscope instrument equipped with Super‐X EDX detector with an X‐FEG module was operated at 300 kV to obtain HAADF‐STEM images.

### 7.3 XRD *φ*‐Scan Measurement

XRD *φ*‐scan was performed to investigate in‐plane ordering of Bi_1−_ *_x_* Sb*_x_* (012)/Cr_1.6_Te_2_. *φ* = 0° was defined by aligning to sapphire [11$\bar{2}$0] according to the *φ*‐scan of the sapphire (02$\bar{2}$4) peaks. As shown in the pole figures in Figure 1c,d, Bi_1−_ *_x_* Sb*_x_* {202} (sapphire (02$\bar{2}$4)) peaks were measured under the condition: X‐ray incident angle *θ* = 24.75° (*θ* = 26.3°) with detector angle 2*θ* = 49.5° (2*θ* = 52.6°) at characteristic polar tilting angle *χ* ≈ 52° and *χ* ≈ 55° (*χ* ≈ 57.6°) [36, 37].

### 7.4 Device Fabrication

The conventional photolithography method, followed by an etching process using an inductively coupled plasma reactive ion etching system (ICP‐RIE), was used to fabricate a CPW device and a standard Hall bar device. For the CPW devices, the transport channel with a width and length of 20 µm was patterned. For the Hall bar devices used in magnetization switching measurements, the channel was patterned into a 20 µm in width and 120 µm in length. 100 nm Au electrodes were deposited using the electron‐beam evaporation method in a vacuum chamber (< 10^−6 ^Torr).

### 7.5 ST‐FMR Measurement

For the ST‐FMR measurements, an *I*_RF_ with frequency (*f*  = *ω*/2π) in the range 4–10 GHz and a nominal power of 20 dBm was applied using a commercial signal generator (SG). The *H_ext_* was swept in‐plane at an angle *θ* = 45° relative to the *I*_RF_ direction to trigger ferromagnetic resonance. In ST‐FMR measurements, SOT‐driven magnetization precession modulates the device resistance via anisotropic magnetoresistance (AMR). With *I*_RF_(*t*) = *I*cos(*ω* *t*) and a time‐varying device resistance *R*(*t*) (where *I* is the amplitude of *I*_RF_), the mixing voltage is produced as *V* (*t*) = *I*_RF_(*t*)·*R*(*t*) = *I*cos(*ω* *t*)·*R*(*t*); the DC component detected using a bias‐tee and a lock‐in amplifier is the *V_mix_*. *V_mix_* can be decomposed into *V*_S_ and *V*_A_ by fitting the *V_mix_* data with F_S_(*H_ext_*) and F_A_(*H_ext_*), respectively, where F_S_(*H_ext_*) = (Δ*H*)^2^ /[(Δ*H*)^2^ + (*H_ext_* − *H*_0_)^2^], and F_A_(*H_ext_*)  = (Δ*H*) (*H_ext_* − *H*_0_)/[(Δ*H*)^2^ + (*H_ext_* − *H*_0_)^2^]. Δ*H* is the linewidth of *V_mix_* in the field domain, and *H*_0_ is the resonance field.

### 7.6 Current‐Induced Magnetization Switching Measurement

The Hall voltage *V_y_* was obtained by measuring the voltage difference, (*V*_*y* +_ − *V*_*y* −_) = *V_y_*, between the two Hall bar electrodes. For instance, when current is applied along the *a*‐axis (*b*‐axis) of single‐domain Bi_1−_ *_x_* Sb*_x_* (012)/Cr_1.6_Te_2_, *V_y_* is the voltage difference between electrodes #3 (#2) and #4 (#1). A physical property measurement system (PPMS) was used to perform magnetization switching measurements. To measure magnetization switching with current applied along the *a*‐axis (*b*‐axis) of single‐domain Bi_1−_ *_x_* Sb*_x_* (012)/Cr_1.6_Te_2_ with 0.1 ≤ *x* ≤ 0.3, ***B***_***x***_ was applied at *θ* = 0° or 180° (*θ* = 90° or 270°), according to the device geometry shown in Figure 3c. Prior to each nominal zero‐field measurement, the degauss mode of the magnet was applied. *I*_pulse_ with a pulse width of 100 µs was applied by a Keithley 6221 current source. After each main *I*_pulse_, *V_y_* was then measured with a Keithley 2182A instrument using a 0.1 mA, 1 ms current pulse.

## Author Contributions

S.R. conceived the study with the help of M.‐H.C. S.R. characterized all samples and fabricated the CPW and Hall bar devices with the help of H.‐J.S., J.H., and D.P. S.R. and Y.L. carried out the ST‐FMR measurements. S.R. carried out current‐induced magnetization switching measurements. S.R. analyzed all data used in this study with the help of M.‐H.C. The manuscript was written by S.R. and revised by M.‐H.C. All authors discussed the data and provided comments on the manuscript. M.‐H.C. supervised all experiments.

## Conflicts of Interest

The authors declare no conflicts of interest.

## Supporting information




**Supporting File**: adma74710‐sup‐0001‐SuppMat.docx.

## Data Availability

The data that support the findings of this study are available from the corresponding author upon reasonable request.
