# Supplementary material for: Deterministic Field‐Free Switching of Perpendicular Magnetization by Bulk Mirror Symmetry Broken Bi1− x Sb x (012) with High Spin Hall Conductivity
Source: Adv Mater. 2026 Aug 25;38(59):e74710. doi: 10.1002/adma.74710 (PMC13689889; doi:10.1002/adma.74710)
Supplement: Supplementary file 1 [file ADMA-38-e74710-s001.docx]

Supporting Information for

**Deterministic field-free switching of perpendicular magnetization by bulk mirror symmetry broken Bi_1−_*_x_*Sb*_x_* (012) with high spin Hall conductivity**

*Seungwon Rho^1,2^***, Youngmin Lee^3,4^, Jaeseok Huh^1^, Jaehan Park^1^, Hyeong-Jun Son^1^, Dowoo Pyeon^1^, and Mann-Ho Cho^1,5^**

^1^Department of Physics, Yonsei University, Seoul 03722, Republic of Korea

^2^Department of Electrical and Computer Engineering, National University of Singapore, Singapore 117582, Singapore

^3^Graduate Program of Semiconductor Science and Engineering, Yonsei University, Seoul 03722, Republic of Korea

^4^Korea Research Institute of Standards and Science, Daejeon 34113, Republic of Korea

^5^Department of System Semiconductor Engineering, Yonsei University, Seoul 03722, Republic of Korea

*Corresponding authors. Email: [mh.cho@yonsei.ac.kr](mailto:mh.cho@yonsei.ac.kr) (M.-H.C.); [swrho@yonsei.ac.kr](mailto:swrho@yonsei.ac.kr) (S.R.)

**TABLE OF CONTENTS**

**Supplementary Note S1. Sample characterization**	2

1.1	Growth of Bi_1−_*_x_*Sb*_x_* (012)/Cr_1.6_Te_2_	3

1.2 Characterization of Bi_1−_*_x_*Sb*_x_* (012)/Cr_1.6_Te_2_	4

1.3 Growth behavior of Bi_1−_*_x_*Sb*_x_* (012)/Cr_1.6_Te_2_	7

1.4 Characterization of Cr_1.6_Te_2_	13

1.5 Surface morphology of Bi_1−_*_x_*Sb*_x_* (012)/Cr_1.6_Te_2_	15

1.6 Electrical properties of Bi_1−_*_x_*Sb*_x_* (012)/Cr_1.6_Te_2_	16

1.7 Magnetic properties of CFB/Bi_1−_*_x_*Sb*_x_* (012)/Cr_1.6_Te_2_	19

**Supplementary Note S2. Symmetry analysis of SOT**	20

**Supplementary Note S3. ST-FMR and SH-HV measurements**	22

3.1 Spin Hall angle extraction	22

3.2 ST-FMR and SH-HV measurements data for Bi_1−_*_x_*Sb*_x_*/Cr_1.6_Te_2_	28

**Supplementary Note S4. Current-induced magnetization switching in single-domain Bi_1−_*_x_*Sb*_x_* (012)/Cr_1.6_Te_2_**	42

4.1 Determination of *J*_C_ using the parallel-resistor model	44

4.2 Current-induced magnetization switching in Bi_1−_*_x_*Sb*_x_* (012) with 0.1 $\text{≤}$ *x* $\text{≤}$ 0.3	44

4.3 Current-induced magnetization switching in Bi_1−_*_x_*Sb*_x_* (012)/[Co/Pt]	52

**Supplementary Note S5. Current-induced magnetization switching in anisotropic three-fold twin domain Bi_1−_*_x_*Sb*_x_* (012)/Cr_1.6_Te_2_**	53

5.1 Validation of the non-vanishing $\boldsymbol{\sigma}_{\boldsymbol{z}}$ in anisotropic three-fold twin domain	53

5.2 Current-induced magnetization switching in Bi_1−_*_x_*Sb*_x_* (012) with 0.4 $\text{≤}$ *x* $\text{≤}$ 0.8	56

**Supplementary Note S6. Estimation of thermal effects induced by pulsed current in magnetization switching**	62

**Supplementary Note S7. Summary of the current-induced magnetization switching in Bi_1−_*_x_*Sb*_x_* (012)/Cr_1.6_Te_2_**	68

**Supplementary References**	70

**Supplementary Note S1. Sample characterization**

**1.1	Growth of** **Bi_1−_*_x_*Sb*_x_* (012)/Cr_1.6_Te_2_**

For Bi_1−_*_x_*Sb*_x_* (012) growth, a textured growth mode typically appears even when a cubic-symmetry substrate is used, making it nearly impossible to obtain single-phase films under conventional conditions.^[1-3]^ Building on our previous work, in which we successfully grew Bi_1−_*_x_*Sb*_x_* (012) (0 < $x<$ 0.9) using Cr_2_Te_3_ buffer layer on sapphire substrate by molecular beam epitaxy (MBE),^[4]^ we further developed an approach that enables either single-domain (0.1 $\leq x\leq$ 0.3) and anisotropic three-fold twin domain (0.4 $\leq x\leq$ 0.8) Bi_1−_*_x_*Sb*_x_* (012) by employing an ultrathin Cr_1.6_Te_2_ buffer layer. We co-evaporated Bi and Sb using individual Knudsen cells to grow the Bi_1−_*_x_*Sb*_x_* layer, and deposited Cr and Te simultaneously using a high-temperature Knudsen cell for Cr and a Knudsen cell for Te to grow the Cr_1.6_Te_2_ layer. The atomic flux of each element was precisely tuned by adjusting the cell temperatures, allowing us to achieve the desired composition for each film. The crystallinity, composition, surface morphology, and electrical resistivity of the layers were systematically characterized using X-ray diffraction (XRD), X-ray photoelectron spectroscopy (XPS), atomic force microscopy (AFM), and a physical property measurement system (PPMS), as described in detail in the Supporting Information.

For the growth of single-phase Bi_1−_*_x_*Sb*_x_* (012) (out-of-plane), a Cr_1.6_Te_2_ buffer layer was first deposited by MBE, followed by the growth of Bi_1−_*_x_*Sb*_x_* layer on top of the Cr_1.6_Te_2_ buffer layer. Notably, when the Bi_1−_*_x_*Sb*_x_* layer was deposited at room temperature (RT) on a 5 nm Cr_1.6_Te_2_ layer without heating or post-annealing, it consistently crystallized in the (012) out-of-plane orientation, mirroring the behavior observed in a previous study using a Cr_2_Te_3_ buffer layer.^[4]^ In contrast, applying post-annealing led to a textured growth mode that produced mixed (001) and (012) orientations, thereby motivating the use of an as-grown process in which the Bi_1−_*_x_*Sb*_x_* layer was deposited at RT on the Cr_1.6_Te_2_ buffer layer without any post-annealing steps. XRD analysis further confirmed that even with a Cr_1.6_Te_2_ buffer layer as thin as 2 nm, the Bi_1−_*_x_*Sb*_x_* layer maintained a single-phase (012) out-of-plane orientation, indicating that Cr_1.6_Te_2_ efficiently screens the crystal potential of the sapphire substrate and serves as a robust template for Bi_1−_*_x_*Sb*_x_* (012) growth.

**1.2	Characterization of Bi_1−_*_x_*Sb*_x_* (012)/Cr_1.6_Te_2_**

**Figure S1.** a) XRD spectra of Bi_1−_*_x_*Sb*_x_* (012) 10 nm/Cr_1.6_Te_2_ 5 nm as a function of Sb concentration $x$. b) XRD spectra of Bi_0.8_Sb_0.2_ (012) $d$ nm/Cr_1.6_Te_2_ 5 nm with $d=$ 3, 4, 5, 6, 7, 8, 10, 12, 15, and 20.

To confirm whether single-phase Bi_1−_*_x_*Sb*_x_* (012) (out-of-plane) growth was achieved, as described in Supplementary Note S1.1, we performed XRD measurements. The XRD spectra for Bi_1−_*_x_*Sb*_x_* (012) 10 nm/Cr_1.6_Te_2_ 5 nm (Bi_1−_*_x_*Sb*_x_* (012)/Cr_1.6_Te_2_) at different Sb concentrations, as shown in Figure S1a, reveal that only the (012) family peak accompanied by clear Laue oscillations appears in Bi_1−_*_x_*Sb*_x_* (012)/Cr_1.6_Te_2_, thereby demonstrating that the Bi_1−_*_x_*Sb*_x_* layers grown on Cr_1.6_Te_2_ layer exhibit excellent crystallinity and a well-defined single-phase orientation. As the Sb concentration increases, all Bi_1−_*_x_*Sb*_x_* diffraction peaks in Figure S1 gradually shift toward higher angles, a trend that can be attributed to the progressive substitution of smaller Sb atoms for Bi, thereby reducing the lattice constant. These results confirm the successful realization of high-crystallinity, single-phase Bi_1−_*_x_*Sb*_x_* (012) out-of-plane films.

As shown in Figure S1b, the XRD spectra of Bi_0.8_Sb_0.2_ (012) $d$ nm/Cr_1.6_Te_2_ 5 nm as a function of thickness $t$ indicate that the (012) peak position remains consistent for thicknesses above ~ 7 nm. At smaller thicknesses, however, the Bi_0.8_Sb_0.2_ peak shifts toward higher angles, indicating the presence of compressive strain. When the film thickness exceeded approximately 7 nm, this shift disappeared, suggesting that strain relaxation occurs above a critical thickness of ~7 nm. Consequently, the 10 nm of Bi_1−_*_x_*Sb*_x_* films used in this study lie within the strain-free regime.

**Figure S2. XPS data and lattice parameter analysis of Bi_1−_*_x_*Sb*_x_* 10 nm.** a) XPS spectra of Bi_1−_*_x_*Sb*_x_* (012) 10 nm/Cr_1.6_Te_2_ 5 nm. b) *a*-axis lattice parameter of Bi_1−_*_x_*Sb*_x_* (012) 10 nm/Cr_1.6_Te_2_ 5 nm.

To achieve the targeted Bi_1−_*_x_*Sb*_x_* composition, the evaporation temperatures of Bi and Sb sources were precisely adjusted, with the Sb concentration in the deposited Bi_1−_*_x_*Sb*_x_* films being verified using in-situ XPS. Figure S2a presents the XPS spectra of Bi_1−_*_x_*Sb*_x_* (012) 10 nm/Cr_1.6_Te_2_ 5 nm/sapphire, along with their corresponding target compositions. The compositions estimated from XPS deviated from the intended values by less than 1%, indicating the high accuracy of the composition control.

To further confirm the increase in Sb concentration in Bi_1−_*_x_*Sb*_x_*, the *a*-axis lattice parameter of Bi_1−_*_x_*Sb*_x_* (012) was extracted from the XRD data shown in Figure S1a using Bragg’s law ($d=\frac{\lambda}{2sin\theta}$), where $d$ is the interplanar spacing of the crystal plane, $\lambda$ is the wavelength of the incident X-ray, and $\theta$ is the Bragg angle corresponding to the diffraction peak. Specifically, the *a*-axis lattice parameter of Bi_1−_*_x_*Sb*_x_* (012) was determined from the (012) peak of the Bi_1−_*_x_*Sb*_x_*/Cr_1.6_Te_2_. As shown in Figure S2b, the *a*-axis lattice parameters decrease linearly with increasing Sb concentration, confirming the substitution of Bi atoms with Sb atoms. The obtained *a*-axis lattice constants are consistent with those shown in Figure 1a, which were referenced from previous studies.

**1.3	Growth behavior of Bi_1−_*_x_*Sb*_x_* (012)/Cr_1.6_Te_2_**

**Figure S3. Growth behavior and in-plane domains of Bi_1−_*_x_*Sb*_x_* (012)/Cr_1.6_Te_2_ 5 nm.** Schematic illustration of a) Single-domain-dominant Bi_1−_*_x_*Sb*_x_* (012), b) Single-domain Bi_1−_*_x_*Sb*_x_* (012), and c) Anisotropic three-fold twin domain Bi_1−_*_x_*Sb*_x_* (012). The XRD $\varphi$-scan peaks of Bi_1−_*_x_*Sb*_x_* {202} for each in-plane orientation are indicated by the red arrows, and the corresponding intensities are denoted as $I$.

The growth mechanism of Bi_1−_*_x_*Sb*_x_* (012) on the transition-metal chalcogenide buffer layer Cr_1+x_Te_2_ is rather complex and corresponds to van der Waals epitaxy, which cannot be explained solely by crystal potential matching.^[4,5]^ Our XRD results suggest that as-grown Bi_1−_*_x_*Sb*_x_* on Cr_1.6_Te_2_ stabilizes in the (012) out-of-plane direction, which has the minimum-interfacial-energy configuration under the influence of the interface between Bi_1−_*_x_*Sb*_x_* and Cr_1.6_Te_2_. However, in-plane ordering is essential for accurately applying current along the mirror-symmetry-broken axis of Bi_1−_*_x_*Sb*_x_* (012)^[6]^ to generate out-of-plane spin ($\boldsymbol{\sigma}_{\boldsymbol{z}}$), as in other low-symmetry materials where $\boldsymbol{\sigma}_{\boldsymbol{z}}$ arises when current is applied along the broken mirror-symmetry direction. To date, no Bi_1−_*_x_*Sb*_x_* (012) film exhibiting in-plane ordering has been reported; thus, field-free magnetization switching has never been observed.^[7,8]^. In such cases, only $\boldsymbol{\sigma}_{\boldsymbol{y}}$ is generated while $\boldsymbol{\sigma}_{\boldsymbol{z}}$ is canceled out, leading to spin–orbit torque (SOT) behavior identical to that of conventional ferromagnet (FM)/heavy metal (HM) systems.

As shown in Figure 1a, Bi_1−_*_x_*Sb*_x_* (012) exhibits a pseudo-cubic surface with a single mirror plane and without two-fold rotational symmetry.^[6,9]^ Moreover, the atomic lattice is tilted out-of-plane around the *b*-axis, and the central atom in the (012) unit cell exhibits inequivalent interatomic distances along this axis (top direction: $\approx$3.1–3.0 Å, bottom direction: $\approx$3.6–3.4 Å). This anisotropic atomic configuration enables the epitaxial growth of out-of-plane Bi_1−_*_x_*Sb*_x_* (012) and the selection of specific Bi_1−_*_x_*Sb*_x_* (012) domains depending on Sb concentration on the Cr_1.6_Te_2_ layer, which has a pseudo-hexagonal atomic arrangement.

***Single-domain case*** (Figure S3b): In the single-domain (α-domain) Bi_1−_*_x_*Sb*_x_* (012), the Bi_1−_*_x_*Sb*_x_* (02$\bar{\text{2}}$) plane lies on top of the *b*-axis direction. When performing $\varphi$-scan measurements of the single-domain Bi_1−_*_x_*Sb*_x_* (012) using Bi_1−_*_x_*Sb*_x_* {202} reflections, the (02$\bar{\text{2}}$) reflection appears as a single $\varphi$ peak at the characteristic tilt angle of $\chi\approx$ 52°, because the broken mirror symmetry removes the in-plane mirror counterpart of the (02$\bar{\text{2}}$) plane.^[10,11]^ Therefore, this azimuth can be aligned with the *b*-axis. In contrast, the other two {202} reflections at $\chi\approx$ 55°, corresponding to the (202) and ($\bar{\text{2}}$22) planes, form a pair of $\varphi$ peaks whose azimuthal angles are orthogonal to the (02$\bar{\text{2}}$) plane, defining the *a*-axis.^[10,11]^ Collectively, the domain type (α or β) of Bi_1−_*_x_*Sb*_x_* (012) can also be determined. Consequently, $\varphi$ peaks of Bi_1−_*_x_*Sb*_x_* {202} exhibit identical intensities if Bi_1−_*_x_*Sb*_x_* (012) remains single-domain: $I_{1}=I_{2}=I_{3}$.

***Single-domain-dominant case*** (Figure S3a): If Bi_1−_*_x_*Sb*_x_* (012) predominantly exhibits single-domain characteristics but includes mixed α- and β-twin domains, two $\varphi$ peaks corresponding to the (02$\bar{\text{2}}$) reflections of the α- and β-domains will appear at tilt angle $\chi\approx$ 52°, separated by 180° in azimuth. Similarly, two $\varphi$ peaks at $\chi\approx$ 55° will be observed, arising from the combined (202) and ($\bar{\text{2}}$22) reflections of the twin domains. In this case, the peak intensities satisfy $I_{1,4}<I_{2}=I_{3}$, but the orientation axes of Bi_1−_*_x_*Sb*_x_* (012) can still be determined.

***Three-fold twin-domain case*** (Figure S3c): If Bi_1−_*_x_*Sb*_x_* (012) grown on Cr_1.6_Te_2_ layer exhibits three-fold twin-domain configuration, $\varphi$ peaks separated by 120° should appear as counterparts of those in the single-domain case.^[10,11]^ Unlike the single-domain scenario, however, the magnitudes of $I_{1}-I_{12}$ vary depending on the fraction of each domain. For perfectly identical three-fold domains, the intensity of (02$\bar{\text{2}}$) peaks at $\chi\approx$ 52° will be exactly half that of the (202) and ($\bar{\text{2}}$22) reflection peaks at $\chi\approx$ 55°. This also applies when α- and β-domains exist in equal proportions. In contrast, if Bi_1−_*_x_*Sb*_x_* (012) exhibits anisotropic three-fold domains or unequal twin-domain ratios, the peak intensities at $\chi\approx$55° and $\chi\approx$52° will differ.^[10,11]^ When each of the three domains selectively adopts one of the twin-domain orientations, the intensities of the $\chi\approx$52° and $\chi\approx$55° peaks become comparable. In this situation, although the crystallographic axes of Bi_1−_*_x_*Sb*_x_* (012) cannot be uniquely determined, the in-plane ordering and domain-fraction distribution can still be analyzed.

**Figure S4. XRD** $\boldsymbol{\varphi}$**-scan data** **of Bi_1−_*_x_*Sb*_x_* (012)/Cr_1.6_Te_2_ 5 nm.** $\varphi$-scan spectra of Bi_1−_*_x_*Sb*_x_* {202} for a) Bi_0.9_Sb_0.1_ (012) 10 nm/Cr_1.6_Te_2_ 5 nm, b) Bi_0.7_Sb_0.3_ (012) 10 nm/Cr_1.6_Te_2_ 5 nm, c) Bi_0.6_Sb_0.4_ (012) 10 nm/Cr_1.6_Te_2_ 5 nm, and d) Bi_0.2_Sb_0.8_ (012) 10 nm/Cr_1.6_Te_2_ 5 nm.

To verify the in-plane ordering of Bi_1−_*_x_*Sb*_x_* (012) grown on the Cr_1.6_Te_2_ layer as a function of Sb concentration, $\varphi$-scan measurements were performed using the Bi_1−_*_x_*Sb*_x_* {202} reflections, as shown in Figure 1c,d, and Figure S4. The Bi_0.8_Sb_0.2_ (012)/Cr_1.6_Te_2_ film exhibits three $\varphi$ peaks with identical intensities, whereas Bi_1−_*_x_*Sb*_x_* (012)/Cr_1.6_Te_2_ with *x* = 0.1 and 0.2 showed anisotropic four $\varphi$ peaks. As shown in Figure S4a,b, the peak at $\varphi=$ 180° (α-domain (02$\bar{\text{2}}$) reflection) appeared with a stronger intensity than that at $\varphi=$ 0° (β-domain (02$\bar{\text{2}}$) reflection), indicating that Bi_1−_*_x_*Sb*_x_* (012) with *x* = 0.1 and 0.2 on the Cr_1.6_Te_2_ layer predominantly forms a single-domain structure mixed with α- and β-domains, with the α-domain being dominant. In the case of Bi_0.7_Sb_0.3_ (012)/Cr_1.6_Te_2_, the intensity of the β-domain (02$\bar{\text{2}}$) reflection peak is approximately half that of the α-domain (02$\bar{\text{2}}$) reflection peak. The coexistence of mixed α- and β-domains within a single-domain Bi_1−_*_x_*Sb*_x_* (012) structure can lead to a reduction in $\boldsymbol{\sigma}_{\boldsymbol{z}}$ generation, since $\boldsymbol{\sigma}_{\boldsymbol{z}}$ components generated in opposite directions within each domain cancel each other. The origin of such domain characteristics lies in the fact that pseudo-hexagonal Cr_1.6_Te_2_ exhibits an anisotropic crystal potential,^[12,13]^ while the lattice constants along each crystallographic axis of Bi_1−_*_x_*Sb*_x_* (012) are inequivalent.^[6]^ Consequently, the central atom within the Bi_1−_*_x_*Sb*_x_* (012) unit cell prefers a specific position relative to the Cr_1.6_Te_2_ surface. Moreover, as the Sb concentration increases in Bi_1−_*_x_*Sb*_x_* (012), the lattice constants decrease, and therefore the emergence of a particular domain configuration at a given lattice constant is physically reasonable. Accordingly, for Bi_1−_*_x_*Sb*_x_* (012)/Cr_1.6_Te_2_ with 0.1 $\text{≤}$ *x* $\text{≤}$ 0.3, we successfully achieved predominantly single-domain Bi_1−_*_x_*Sb*_x_* (012) films. The single-domain nature of Bi_1−_*_x_*Sb*_x_* (012) is preserved even when the Cr_1.6_Te_2_ buffer layer is reduced to 2 nm.

As shown in Figure S4c,d, for Bi_1−_*_x_*Sb*_x_*/Cr_1.6_Te_2_ with 0.4 $\text{≤}$ *x* $\text{≤}$ 0.8, twelve $\varphi$ peaks with anisotropic intensities were observed. Specifically, Bi_0.6_Sb_0.4_ (012)/Cr_1.6_Te_2_, the intensity of the (02$\bar{\text{2}}$) peaks at $\chi\approx$ 52° is nearly half that of the (202) and ($\bar{\text{2}}$22) reflection peaks at $\chi\approx$ 55°, indicating that Bi_0.6_Sb_0.4_ (012) on the Cr_1.6_Te_2_ layer consists of nearly identical three-fold domains with equal fractions of α- and β-domains.^[10,11]^ We believe that this structural characteristic explains the weak out-of-plane SOT, small *H*_comp_, and high *J*_C_ observed in spin-torque ferromagnetic resonance (ST-FMR) and current-induced magnetization switching measurements of Bi_0.6_Sb_0.4_ (012)/Cr_1.6_Te_2_. In contrast, for Bi_1−_*_x_*Sb*_x_*/Cr_1.6_Te_2_ with *x* = 0.6 and 0.8, the peak intensities at $\chi\approx$ 52° differ, with the α-domain (02$\bar{\text{2}}$) reflection being notably stronger. Additionally, the intensities of the peaks at $\chi\approx$ 55° are unequal, and the intensities of the peaks at $\chi\approx$ 52° and $\chi\approx$ 55° are almost identical, indicating that Bi_1−_*_x_*Sb*_x_*/Cr_1.6_Te_2_ with *x* = 0.6 and 0.8 exhibits anisotropic three-fold domains with a dominant α-domain component.^[10,11]^ This three-fold domain characteristic can be understood as follows: unlike the predominantly single-domain Bi_1−_*_x_*Sb*_x_* (012)/Cr_1.6_Te_2_ films with 0.1 $\text{≤}$ *x* $\text{≤}$ 0.3, where larger lattice constants favor a specific in-plane orientation, the Bi_1−_*_x_*Sb*_x_* (012) films with 0.4 $\text{≤}$ *x* $\text{≤}$ 0.8 exhibit smaller lattice constants, leading to a reduced difference in the anisotropic crystal potential of the pseudo-hexagonal Cr_1.6_Te_2_ surface. As a result, the energy difference among the three in-plane axes separated by 120° becomes negligible, enabling Bi_1−_*_x_*Sb*_x_* (012) to be stabilized in three-fold twin domains. In the single-domain regime, the in-plane ordering tends to align preferentially along a specific axis of Cr_1.6_Te_2_—along the sapphire [11$\bar{\text{2}}$0] direction—under the influence of the anisotropic potential of Cr_1.6_Te_2_.

**1.4	Characterization of Cr_1.6_Te_2_**

**Figure S5. Characterization of Cr_1.6_Te_2_.** a,b) Top-view EDS layered images of Cr_1.6_Te_2_/sapphire (1 cm $\times$ 1 cm) without and with oxygen, respectively. c) Tables showing the atomic concentrations extracted from the images in (a) and (b). d) AFM image of Cr_1.6_Te_2_ 5 nm/sapphire with a 1 μm scale bar. e) XRD spectrum of Cr_1.6_Te_2_ 5 nm/sapphire. f) Temperature-dependent resistivity of Cr_1.6_Te_2_ 5 nm/sapphire measured using the four-point probe method in a standard Hall bar device.

As shown in Figure S5a,b, the energy-dispersive spectroscopy (EDS) elemental mapping images of a 125 μm $\times$ 100 μm region on the 1 cm $\times$ 1 cm Cr_1.6_Te_2_ 5 nm/sapphire are displayed, excluding and including oxygen, respectively. From the EDS spectra in Figure S5a,b, the atomic concentrations of Cr and Te were evaluated using the K and L emission lines, as summarized in Figure S5c. The Cr:Te ratios were determined to be approximately 1.6:2 in both cases (44.40:55.60 and 0.2:0.25 in nominal values, respectively), confirming that the film composition corresponds to Cr_1.6_Te_2_.

AFM measurements were performed to examine the surface morphology of Cr_1.6_Te_2_ with a SiO_2_ capping layer, as shown in Figure S5d. The Cr_1.6_Te_2_ 5 nm film with a 2 nm SiO_2_ capping layer exhibits an atomically flat surface with a surface roughness of approximately 0.220 nm. This result indicates that the Cr_1.6_Te_2_ 5 nm serves as a suitable buffer layer for the growth of Bi_1−_*_x_*Sb*_x_* (012), providing a sufficiently flat surface. As shown in Figure S5e, XRD measurements were conducted to investigate the crystallinity of the Cr_1.6_Te_2_ 5 nm. The XRD spectrum displays only the (002) family of peaks with clear Laue oscillations, demonstrating the high crystallinity of the film. The resistivity versus temperature (*R*–*T*) curve of Cr_1.6_Te_2_ is shown in Figure S5f, revealing a ferromagnetic transition near 190 K. The resistivity of Cr_1.6_Te_2_ 5 nm at 150 K (300 K) is approximately 3.48$\times\text{10}^{-\text{6}} \Omega\cdot m$ (3.65$\times\text{10}^{-\text{6}} \Omega\cdot m$).

**1.5	Surface morphology of Bi_1−_*_x_*Sb*_x_* (012)/Cr_1.6_Te_2_**

**Figure S6. AFM images of Bi_1−_*_x_*Sb*_x_* (012) 10 nm/Cr_1.6_Te_2_ 5 nm.** a) *x* = 0.2, b) *x* = 0.4, c) *x* = 0.6, d) *x* = 0.8 with a 1 μm scale bar.

Because SOT generation is sensitive to interfacial quality, obtaining a smooth surface of the SOT source layer is crucial since a clean and uniform interface enhances spin transmission efficiency across the interface. To evaluate the surface flatness of the samples used in this study, AFM measurements were performed, and representative images are shown in Figure S6. As shown in Figure S6a–d, Bi_1−_*_x_*Sb*_x_* (012) 10 nm/Cr_1.6_Te_2_ 5 nm with $x=$ 0.2, 0.4, 0.6, and 0.8 exhibited flat surfaces with a surface roughness of 0.172 nm, 0.557 nm, 0.459 nm, and 0.343 nm, respectively. In addition to these representative samples, all Bi_1−_*_x_*Sb*_x_* (012) 10 nm/Cr_1.6_Te_2_ 5 nm heterostructures demonstrated surface roughness values below 10% of the nominal thickness, confirming the formation of clean interfaces. Consequently, we achieved flat and well-defined surfaces in Bi_1−_*_x_*Sb*_x_* (012) 10 nm/Cr_1.6_Te_2_ 5 nm, suitable for reliable ST-FMR and current-induced magnetization-switching measurements.

**1.6	Electrical properties of Bi_1−_*_x_*Sb*_x_* (012)/Cr_1.6_Te_2_**

**Figure S7. Transport properties of Bi_1−_*_x_*Sb*_x_*/Cr_1.6_Te_2_.** a) Schematic illustration of the standard Hall bar device and the four-point probe measurement method. b) Resistivity versus temperature (*R*–*T*) curves of Bi_0.8_Sb_0.2_ (012) 10 nm/Cr_1.6_Te_2_ 2 nm. c) Resistivity of Bi_1−_*_x_*Sb*_x_* (012)/Cr_1.6_Te_2_ and Bi_1−_*_x_*Sb*_x_* (012) as a function of *x*, measured at 150 K. For Bi_1−_*_x_*Sb*_x_* (012), the resistivity was calculated using the parallel resistor model. d) Conductivity of Bi_1−_*_x_*Sb*_x_* (012) 10 nm/Cr_1.6_Te_2_ 5 nm as a function of *x*, measured at 300 K.

We measured the resistivity and charge conductivity ($\sigma_{C}$) of Bi_1−_*_x_*Sb*_x_* (012) 10 nm/Cr_1.6_Te_2_ 5 nm and 2 nm as a function of Sb concentration using a standard Hall bar device (Figure S7a). As illustrated in Figure S7a, the longitudinal resistivity ($\rho_{xx}$) was measured using the four-point probe method, in which a direct current (I_DC_) of 10^−5^ A was applied between the source and the ground electrodes using a source meter (Keithley 2400). The voltage difference (V_xx_) between two Hall bar electrodes was then recorded to calculate $\rho_{xx}$. The Hall bar devices used in this study have transport channels with both a width and length of 20 μm. Accordingly, $\rho_{xx}$ was calculated using $\rho_{xx}=\frac{V_{xx}}{\text{10}^{\text{-}\text{5}}\text{ A}}\cdot t$, where $t$ is the thickness of the sample. The corresponding $\sigma_{C}$ was obtained from $\sigma_{C}=1/\rho_{xx}$. V_xx_ was measured using a nanovoltmeter (Keithley 2182A).

First, we measured the $\rho_{xx}$ of Bi_1−_*_x_*Sb*_x_* (012) 10 nm/Cr_1.6_Te_2_ 2 nm ($\rho_{\mathrm{stack}}$) and then extracted the resistivity of the Bi_1−_*_x_*Sb*_x_* (012) layer alone ($\rho_{\mathrm{BiSb}}$) using a parallel-resistor model. The extracted $\rho_{\mathrm{BiSb}}$ values were used in the main manuscript to evaluate the SOT switching power density (P_SOT_). To accurately determine $\rho_{\mathrm{BiSb}}$ itself, we used Bi_1−_*_x_*Sb*_x_* (012) 10 nm/Cr_1.6_Te_2_ 2 nm, in which the Bi_1−_*_x_*Sb*_x_* (012) layer exhibits the same domain structure as in the thicker-buffer-layer samples, while the Cr_1.6_Te_2_ layer is thinner. The parallel-resistor model is expressed in Equation (S1), where $\rho_{\mathrm{Buffer}}$ is the resistivity of the Cr_1.6_Te_2_ layer, and $t_{\mathrm{BiSb}}$ and $t_{\mathrm{buffer}}$ are the thicknesses of the Bi_1−_*_x_*Sb*_x_* (012) and Cr_1.6_Te_2_ layers, respectively.

$$\frac{1}{\rho_{\mathrm{stack}}}=\frac{t_{\mathrm{BiSb}}}{\rho_{\mathrm{BiSb}}(t_{\mathrm{BiSb}}+t_{\mathrm{buffer}})}+\frac{t_{\mathrm{Buffer}}}{\rho_{\mathrm{Buffer}}(t_{\mathrm{BiSb}}+t_{\mathrm{buffer}})}$$

**(S1)**

The *R*–*T* curve for a representative Bi_0.8_Sb_0.2_ (012) 10 nm/Cr_1.6_Te_2_ 2 nm sample is shown in Figure S7b. For Bi_0.8_Sb_0.2_ (012) 10 nm/Cr_1.6_Te_2_ 2 nm, the sample exhibits typical semiconducting transport behavior, with $\rho_{xx}$ approximately 308 $\mu\Omega\cdot cm$ at 300 K and 370 $\mu\Omega\cdot cm$ at 150 K. We extracted $\rho_{\mathrm{stack}}$ and $\rho_{\mathrm{BiSb}}$ at 150 K, because the current-induced magnetization switching measurements in the main manuscript were performed at 150 K, as shown in Figure S7c. We also extracted $\sigma_{C}$ for Bi_1−_*_x_*Sb*_x_* (012) 10 nm/Cr_1.6_Te_2_ 5 nm at 300 K, which was used as the SOT source layer in the ST-FMR measurements to estimate the spin Hall conductivity, as shown in Figure S7d. All channels of the Hall bar devices are aligned at $\varphi=-\text{90°}$ relative to the sapphire [11$\bar{\text{2}}$0] direction. The $\rho_{\mathrm{stack}}$ and $\rho_{\mathrm{BiSb}}$ values at 150 K and the $\sigma_{C}$ values of Bi_1−_*_x_*Sb*_x_* (012) 10 nm/Cr_1.6_Te_2_ 5 nm at 300 K are summarized in Table S1.

**Table S1. Summary of** $\boldsymbol{\rho}_{\boldsymbol{xx}}$ **and** $\boldsymbol{\sigma}_{\mathbf{C}}$ **for Bi_1−_*_x_*Sb*_x_* (012) 10 nm/Cr_1.6_Te_2_ 2 nm, Bi_1−_*_x_*Sb*_x_* (012), and Bi_1−_*_x_*Sb*_x_* (012) 10 nm/Cr_1.6_Te_2_ 5 nm.**

| Samples | $\rho_{xx}$  Bi_1−_*_x_*Sb*_x_*/Cr_1.6_Te_2_ 2 nm  (150 K) [$\mu\Omega\mathrm{cm}$] | $\rho_{xx}$ (parallel-resistor model) Bi_1−_*_x_*Sb*_x_* (012)  (150 K) [$\mu\Omega\mathrm{cm}$] | $\sigma_{C}$ Bi_1−_*_x_*Sb*_x_*/Cr_1.6_Te_2_ 5 nm  (300 K) [10^5^$\Omega^{-1}m^{-1}$] |
| --- | --- | --- | --- |
| $x$ = 0.1 | 421 | 469 | 2.41 |
| $x$ = 0.2 | 370 | 382 | 2.70 |
| $x$= 0.3 | 334 | 327 | 3.07 |
| $x$ = 0.4 | 270 | 243 | 3.83 |
| $x$ = 0.6 | 206 | 171 | 4.92 |
| $x$ = 0.8 | 174 | 139 | 5.81 |

**1.7	Magnetic properties of CFB/Bi_1−_*_x_*Sb*_x_* (012)/Cr_1.6_Te_2_**

**Figure S8.** VSM data of CFB 5 nm/Bi_1−_*_x_*Sb*_x_* (012) 10 nm/Cr_1.6_Te_2_ 5 nm, plotted as $M_{s}\cdot t$.

To examine the magnetic properties of the ferromagnetic material (FM) layer CoFeB (CFB) in CFB/Bi_1−_*_x_*Sb*_x_* (012)/Cr_1.6_Te_2_, we measured magnetization versus H-field (*M*–*H*) curves using a vibrating sample magnetometer (VSM). In these measurements, the quantity $M_{s}\cdot t$ corresponds directly to the raw experimental output because it is defined as the measured magnetic moment normalized by the detection area specified in the instrument specifications, where $M_{s}$ and $t$ denote the saturation magnetization and thickness of the FM layer, respectively. Therefore, the *M*–*H* curves were plotted with $M_{s}\cdot t$ on the vertical axis, as this parameter is essential for determining the spin Hall angle ($\theta_{\mathrm{SH}}$) in the ST-FMR measurements.^[14]^ As shown in Figure S8b, the *M*–*H* curves of CFB 5 nm/Bi_1−_*_x_*Sb*_x_* (012) 10 nm/Cr_1.6_Te_2_ 5 nm (CFB/Bi_1−_*_x_*Sb*_x_* (012)/Cr_1.6_Te_2_) exhibit large remanent magnetization and soft magnetic behavior with small coercive fields. In particular, the coercivity of CFB/Bi_1−_*_x_*Sb*_x_* (012)/Cr_1.6_Te_2_ gradually increases with Sb concentration, while the films still retain soft magnetic characteristics. The extracted $M_{s}\cdot t$ values for CFB/Bi_1−_*_x_*Sb*_x_* (012)/Cr_1.6_Te_2_ are approximately 6250$\times$10^−7^ emu/cm^2^.

**Supplementary Note S2. Symmetry analysis of SOT**

During the ST-FMR measurements, the in-plane spin ($\boldsymbol{\sigma}_{\boldsymbol{y}}$)- and out-of-plane spin ($\boldsymbol{\sigma}_{\boldsymbol{z}}$)-induced SOTs can be described as in Equation (1) of the main manuscript,^[15-18]^ where $\boldsymbol{\tau}_{\mathrm{SOT}}$ is the total SOT, $\tau_{DL,y}$ ($\tau_{DL,z}$) is in-plane spins ($\boldsymbol{\sigma}_{\boldsymbol{y}}$)-induced ($\boldsymbol{\sigma}_{\boldsymbol{z}}$-induced) damping like torque, $\tau_{FL,y}$ ($\tau_{FL,z}$) is $\boldsymbol{\sigma}_{\boldsymbol{y}}$-induced ($\boldsymbol{\sigma}_{\boldsymbol{z}}$-induced) field like torque, and $\boldsymbol{m}$ is the magnetization direction. Let $\boldsymbol{m}=(cos\theta, sin\theta,0$), where $\theta$ is the in-plane angle relative to the *x*-axis ($\theta<$ 90°). The spin polarization directions are defined as $\boldsymbol{\sigma}_{\boldsymbol{y}}=$ $(0,1,0)$ and $\boldsymbol{\sigma}_{\boldsymbol{z}}=(0,0,1)$

$$\boldsymbol{\tau}_{\mathrm{SOT}}=\tau_{DL,y}\cdot\boldsymbol{m}\times\boldsymbol{\sigma}_{\boldsymbol{y}}\times\boldsymbol{m}\boldsymbol{-}\tau_{FL,y}\cdot\boldsymbol{m}\times\boldsymbol{\sigma}_{\boldsymbol{y}}\boldsymbol{+}\tau_{DL,z}\cdot\boldsymbol{m}\times\boldsymbol{\sigma}_{\boldsymbol{z}}\times\boldsymbol{m-}\tau_{FL,z}\cdot\boldsymbol{m}\times\boldsymbol{\sigma}_{\boldsymbol{z}}$$

**(1)**

The generation of specific spin orientations in spin–orbit materials is determined by symmetry operations under linear response,^[19,20]^ as represented in Equation (S1), where $s_{i}$ is the spin orientation generated by an applied electric field $E_{j}$ along the *j*-axis, and $\chi_{ij}$ is the spin–charge conversion tensor.

$$s_{i}=\chi_{ij}E_{j}$$

**(S1)**

For ***conventional SOT systems***, where mirror symmetry and two-fold rotational symmetry (C_2_ symmetry) are preserved, only $\boldsymbol{\sigma}_{\boldsymbol{y}}$ is symmetry-allowed. Under a mirror operation with respect to the *xz*-plane, $\sigma_{v}(xz)$, the polar vector $(x,y,z)$ transforms as $\sigma_{v}(xz)(x,y,z)\to(x,-y,z)$. The corresponding rotational operation matrix can be expressed as $R=\text{diag(1,-1,1)}$ with $\det\left( R \right)=-\text{1}$. Then, $\sigma_{v}(xz)$ on the pseudovector $s_{i}$ is $\sigma_{v}(xz) s_{i}=\det\left( R \right)Rs_{i}$. Consequently, applying $\sigma_{v}(xz)$ to Equation (S1) with $s_{z}$ and $E_{x}$ ($s_{z}=\chi_{zx} E_{x})$ yields ${\sigma_{v}(xz)s}_{z}=\chi_{zx} \sigma_{v}(xz)E_{x}\to{-s}_{z}=\chi_{zx} E_{x}$, which gives $s_{z}={-s}_{z}$, leading to $\chi_{zx}=0$. Therefore, the generation of $\boldsymbol{\sigma}_{\boldsymbol{z}}$ is forbidden by symmetry.^[15]^ In contrast, for $\boldsymbol{\sigma}_{\boldsymbol{y}}$ generation, applying $\sigma_{v}(xz)$ to Equation (S1) with $s_{y}$ and $E_{x}$ ($s_{y}=\chi_{yx} E_{x})$ yields ${\sigma_{v}(xz)s}_{y}=\chi_{yx} \sigma_{v}(xz)E_{x}\to s_{y}=\chi_{yx} E_{x}$. Therefore, $\boldsymbol{\sigma}_{\boldsymbol{y}}$ generation is allowed because it remains invariant under $\sigma_{v}(xz)$.

For ***low-symmetry materials***, where mirror symmetry is broken at the *xz*-plane and C_2_ symmetry is absent, $\boldsymbol{\sigma}_{\boldsymbol{z}}$ generation becomes symmetry-allowed. In this case, the only remaining mirror operation is with respect to the *yz*-plane, $\sigma_{v}\left( yz \right)$, since no mirror plane exists along the *xz*-plane without C_2_ symmetry.^[15-18]^ Under a mirror operation with respect to the *yz*-plane, the polar vector $(x,y,z)$ transforms as $\sigma_{v}(yz)(x,y,z)\to(-x,y,z)$. The corresponding rotational operation matrix is $R=\text{diag(-1,1,1)}$ with $\det\left( R \right)=-\text{1}$. Then, $\sigma_{v}(yz)$ on the pseudovector $s_{i}$ is $\sigma_{v}(yz) s_{i}=\det\left( R \right)Rs_{i}$. Consequently, applying $\sigma_{v}(yz)$ to Equation (S1) with $s_{z}$ and $E_{x}$ ($s_{z}=\chi_{zx} E_{x})$ yields ${\sigma_{v}(yz)s}_{z}=\chi_{zx} \sigma_{v}\left( yz \right)E_{x}\to{-s}_{z}=-\chi_{zx} E_{x}$, indicating that $\boldsymbol{\sigma}_{\boldsymbol{z}}$ generation is allowed because it remains invariant under $\sigma_{v}(yz)$. Similarly, for $\boldsymbol{\sigma}_{\boldsymbol{y}}$ generation, applying $\sigma_{v}(yz)$ to Equation (S1) with $s_{y}$ and $E_{x}$ ($s_{y}=\chi_{yx} E_{x})$ yields ${\sigma_{v}(yz)s}_{y}=\chi_{yx} \sigma_{v}\left( yz \right)E_{x}\to{-s}_{y}=-\chi_{yx} E_{x}$. Therefore, $\boldsymbol{\sigma}_{\boldsymbol{y}}$ generation is also symmetry-allowed because it is invariant under $\sigma_{v}(yz)$. As a result, in low-symmetry materials, both $\boldsymbol{\sigma}_{\boldsymbol{y}}$ and $\boldsymbol{\sigma}_{\boldsymbol{z}}$ generation are allowed by symmetry.

To examine the generation of $\boldsymbol{\sigma}_{\boldsymbol{z}}$-induced SOTs, it is necessary to consider how specific current-induced SOT components transform under symmetry operations. A particular SOT component is symmetry-allowed only when the sign of the torque changes with the sign of the current under the corresponding symmetry operations. For instance, under the $\sigma_{v}(xz)$ in conventional SOT systems with current $I\parallel x$, the transformations are as follows: $I\boldsymbol{\to}I$, $\boldsymbol{m (}\theta\boldsymbol{)\to m (-}\theta\boldsymbol{)}$, $\boldsymbol{\sigma}_{\boldsymbol{y}}\boldsymbol{\to}\boldsymbol{\sigma}_{\boldsymbol{y}}$, and $\boldsymbol{\sigma}_{\boldsymbol{z}}\boldsymbol{\to}-\boldsymbol{\sigma}_{\boldsymbol{z}}$, because $\boldsymbol{m}$, $\boldsymbol{\sigma}_{\boldsymbol{y}}$, and $\boldsymbol{\sigma}_{\boldsymbol{z}}$ are pseudovectors.^[15]^ This operation results in a sign change of $\tau_{DL,z}$ ($\sigma_{v}\left( xz \right)\boldsymbol{m}\times\boldsymbol{\sigma}_{\boldsymbol{z}}\times\boldsymbol{m}\boldsymbol{\to-m}\times\boldsymbol{\sigma}_{\boldsymbol{z}}\times\boldsymbol{m}$) without reversing the sign of the current. Therefore, $\tau_{DL,z}$ is forbidden by symmetry, consistent with the linear response theory, explaining why $\boldsymbol{\sigma}_{\boldsymbol{z}}$-induced SOTs are not generated in conventional SOT systems. In contrast, $\boldsymbol{\sigma}_{\boldsymbol{z}}$-induced SOTs can arise in low-symmetry materials. For low-symmetry materials, only mirror operations with respect to the *yz*-plane can be applied to determine the sign change of SOTs, because no mirror plane exists along the *xz*-plane in the absence of C_2_ symmetry. Consequently, under $\sigma_{v}(yz)$ in low-symmetry materials with current $I\parallel x$, the transformations are as follows: $I\boldsymbol{\to-}I$, $\boldsymbol{m (}\theta\boldsymbol{)\to m (}\text{180}\text{°}\boldsymbol{-}\theta\boldsymbol{)}$, $\boldsymbol{\sigma}_{\boldsymbol{y}}\boldsymbol{\to}\boldsymbol{-\sigma}_{\boldsymbol{y}}$, and $\boldsymbol{\sigma}_{\boldsymbol{z}}\boldsymbol{\to}-\boldsymbol{\sigma}_{\boldsymbol{z}}$, because $\boldsymbol{m}$, $\boldsymbol{\sigma}_{\boldsymbol{y}}$, and $\boldsymbol{\sigma}_{\boldsymbol{z}}$ are pseudovectors. This operation produces a sign change in $\tau_{DL,z}$ ($\sigma_{v}\left( yz \right)\boldsymbol{m}\times\boldsymbol{\sigma}_{\boldsymbol{z}}\times\boldsymbol{m}\boldsymbol{\to-m}\times\boldsymbol{\sigma}_{\boldsymbol{z}}\times\boldsymbol{m}$) accompanied by a reversal of the current direction. Therefore, $\tau_{DL,z}$ is symmetry-allowed in low-symmetry materials, consistent with symmetry-based considerations.

**Supplementary Note S3. ST-FMR and SH-HV measurements**

**3.1	Spin Hall angle extraction**

***ST-FMR measurement:*** The $V_{mix}$ signals consist of a symmetric voltage component ($V_{S}$) and an antisymmetric voltage component ($V_{A}$), which can be expressed as $V_{mix}=V_{S}+V_{A}=\tilde{V_{S}}\text{F}_{\text{S}}+\tilde{V_{A}}\text{F}_{\text{A}}$, where $\tilde{V_{S}}$ ($\tilde{V_{A}}$) is the amplitude of $V_{S}$ ($V_{A}$), and $\text{F}_{\text{S}}$ ($\text{F}_{\text{A}}$) is symmetric (antisymmetric) Lorentzian functions, respectively. The $V_{mix}$ obtained from ST-FMR measurements, where both $\boldsymbol{\sigma}_{\boldsymbol{y}}$-and $\boldsymbol{\sigma}_{\boldsymbol{z}}$-induced SOTs are allowed, is described by Equation (S2),^[14,17,21,22]^ where $R_{d}$ is the device resistance, $\gamma$ is the gyromagnetic ratio, and $f$ is the frequency of the I_RF_. $F_{S}(H_{ext})=\left( \Delta H \right)^{2}/[\left( \Delta H \right)^{2}+\left( H_{ext}-H_{0} \right)^{2}]$, and $F_{A}(H_{ext})=\left( \Delta H \right)\left( H_{ext}-H_{0} \right)/[\left( \Delta H \right)^{2}+\left( H_{ext}-H_{0} \right)^{2}]$, where $\Delta H$ is the linewidth of $V_{mix}$ in the field domain and $H_{0}$ is the resonance field. $\tilde{D}$ is defined as $\left[ 1+\left( {\mu_{0}M_{eff}}/{H_{ext}} \right) \right]^{1/2}$, where $\mu_{0}$ and $M_{eff}$ are the vacuum permeability and effective magnetization, respectively. In this study, the external magnetic field ($H_{ext}$) was swept at an angle $\theta=\text{45°}$ relative to the I_RF_ direction.

$$V_{\mathrm{mix}}=-\frac{1}{4}\frac{dR_{d}}{d\theta}\frac{\gamma I_{RF}cos\theta}{\Delta H 2\pi\left( {df}/{dH_{ext}} \right)\left. \right|_{H_{ext}=H_{0}}}[(\tau_{DL,y}+\tau_{FL,z})F_{S}\left( H_{ext} \right)+(\tau_{\mathrm{Oe}}\boldsymbol{+}\tau_{FL,y}\boldsymbol{+}\tau_{DL,z}\boldsymbol{)}\tilde{D}F_{A}\left( H_{ext} \right)]$$

**(S2)**

Considering the symmetry of SOTs in ST-FMR measurements, each voltage component generated by a specific spin-induced SOT can be separated. For example, $\tau_{DL,y}$ and $\tau_{DL,z}$ do not change sign when $H_{ext}$ is reversed: $H_{ext}\left( \theta\right)\to H_{ext}\left( 180^{\circ}+\theta\right)=-H_{ext}\left( \theta\right)$, which leads to $\boldsymbol{m \to-m}$. In contrast, $\tau_{FL,y}\left( \boldsymbol{m} \right)=-\tau_{FL,y}\left( \boldsymbol{-m} \right)$, and $\tau_{FL,z}\left( \boldsymbol{m} \right)=-\tau_{FL,z}\left( \boldsymbol{-m} \right)$. Therefore, the $\tau_{DL,y}$-induced voltage components ($\text{V}_{DL,y}$), ($\tau_{FL,y}+\tau_{\mathrm{Oe}}$)-induced voltage components ($\text{V}_{FL+Oe,y}$), $\tau_{DL,z}$-induced voltage components ($\text{V}_{DL,z}$), and $\tau_{FL,z}$-induced voltage components ($\text{V}_{FL,z}$) can be extracted using Equations (S3)–(S6)^[18]^: $V_{S}\left( H_{ext} \right)=V_{DL,y}+V_{FL,z}$ ,$V_{S}\left( -H_{ext} \right)={-V}_{DL,y}+V_{FL,z}$, $V_{A}\left( H_{ext} \right)=V_{FL+Oe,y}+V_{DL,z}$, and $V_{A}\left( -H_{ext} \right)=V_{FL+Oe,y}-V_{DL,z}$, because reversing $H_{ext}\left( \theta\right)\to-H_{ext}\left( \theta\right)$ leads to $cos\theta\to-cos\theta$.

$$\text{V}_{DL,y}=\frac{V_{S}\left( H_{ext} \right)-V_{S}\left( {-H}_{ext} \right)}{2}$$

**(S3)**

$$\text{V}_{FL+Oe,y}=\frac{V_{A}\left( H_{ext} \right)\text{ }\text{+}\text{ }V_{A}\left( {-H}_{ext} \right)}{2}$$

**(S4)**

$$\text{V}_{DL,z}=\frac{V_{A}\left( H_{ext} \right)-V_{A}\left( {-H}_{ext} \right)}{2}$$

**(S5)**

$$\text{V}_{FL,z}=\frac{V_{S}\left( H_{ext} \right)+V_{S}\left( {-H}_{ext} \right)}{2}$$

**(S6)**

In topological materials such as Bi_1−_*_x_*Sb*_x_* used in this study, $\tau_{\mathrm{FL}}$ is non-negligible.^[4]^ Therefore, it is appropriate to employ a voltage-component-only approach, such as the $V_{S}$-only method,^[14]^ for extracting the $\boldsymbol{\sigma}_{\boldsymbol{y}}$-induced spin Hall angle ($\theta_{\mathrm{SH}}$) in SOT systems, where only $\boldsymbol{\sigma}_{\boldsymbol{y}}$ is symmetry-allowed. For systems in which $\boldsymbol{\sigma}_{\boldsymbol{z}}$ generation is also allowed, the V_S_-only method can be extended, as expressed in Equation (S7), where $\sigma_{SH,y}$ is the in-plane spin Hall conductivity, $E$ is the electric field across the coplanar waveguide (CPW) device, and $t_{FM}$ is the thickness of the FM layer.

| $\text{V}_{DL,y}=-\frac{1}{4}\frac{dR_{d}}{d\theta}\frac{\gamma I_{RF}cos\theta}{\Delta H 2\pi\left( {df}/{dH_{ext}} \right)\left. \right\vert_{H_{ext}=H_{0}}}\tau_{DL,y}F_{S}\left( H_{ext} \right)$ |  |
| --- | --- |
| $\tau_{DL,y}=\frac{-2\left[ \tilde{V_{S}(}H_{ext} \right)-{\tilde{V_{S}(}-H}_{ext})]\Delta H 2\pi\left( {df}/{dH_{ext}} \right)\left. \right\vert_{H_{ext}=H_{0}}}{{(dR_{d}}/{d\theta})\left. \right\vert_{\theta=45^{\circ}}\gamma I_{RF}cos\theta}$ | **(S7)** |
| $\sigma_{SH,y}=\frac{\hbar}{2e}\frac{J_{S}}{E}\boldsymbol{=}\frac{\tau_{DL,y}M_{s}t_{FM}}{E}$, $\theta_{\mathrm{SH}}=\frac{2e}{\hbar}\frac{\sigma_{SH,y}}{\sigma_{C}}$ |  |

The $\boldsymbol{\sigma}_{\boldsymbol{z}}$-induced spin Hall angle ($\theta_{SH,z}$) can be evaluated using the following equations, where $\sigma_{SH,z}$ is the out-of-plane spin Hall conductivity. In contrast to the $\theta_{\mathrm{SH}}$ extraction, the factor $\tilde{D}=\left[ 1+\left( {\mu_{0}M_{eff}}/{H_{ext}} \right) \right]^{1/2}$ must be included when determining $\theta_{SH,z}$. Hereafter, we refer to this analysis as the V_A,_*_z_*-only method.

| $V_{DL,z}=-\frac{1}{4}\frac{dR_{d}}{d\theta}\frac{\gamma I_{RF}cos\theta}{\Delta H 2\pi\left( {df}/{dH_{ext}} \right)\left. \right\vert_{H_{ext}=H_{0}}}\tau_{DL,z}\tilde{D}F_{A}\left( H_{ext} \right)$ |  |
| --- | --- |
| $\tau_{DL,z}=\frac{-2\left[ \tilde{V_{A}(}H_{ext} \right)-{\tilde{V_{A}(}-H}_{ext})]\Delta H 2\pi\left( {df}/{dH_{ext}} \right)\left. \right\vert_{H_{ext}=H_{0}}}{{(dR_{d}}/{d\theta})\left. \right\vert_{\theta=45^{\circ}}\gamma I_{RF}cos\theta\tilde{D}}$ | **(S8)** |
| $\sigma_{SH,z}=\frac{\hbar}{2e}\frac{J_{S}}{E}\boldsymbol{=}\frac{\tau_{DL,z}M_{s}t_{FM}}{E}$, $\theta_{SH,z}=\frac{2e}{\hbar}\frac{\sigma_{SH,z}}{\sigma_{C}}$ |  |

However, the $V_{S}$-only and $V_{A,z}$-only methods have the limitation of requiring additional measurements of several parameters, including $\Delta H$, $\tilde{D}$, $\gamma$, $\left( {df}/{dH} \right)\left. \right|_{H_{ext}=H_{0}}$, $\sigma_{C}$, $M_{s}t_{FM}$, the magnitude of *I*_RF_ $E$, and ${(dR_{d}}/{d\theta})\left. \right|_{\theta=45^{\circ}}$. All these parameters were determined for CFB/Bi_1−_*_x_*Sb*_x_* (012)/Cr_1.6_Te_2_ at 300 K. $\Delta H$ was obtained by fitting the $V_{mix}$ signals with Lorentzian functions. By fitting the $H_{0}$ of the $V_{mix}$ as a function of $f$using the Kittel formula$: f=\gamma/2\pi\sqrt{H_{0}(H_{0}+\mu_{0}M_{eff})}$ , we extracted the fitting parameters $\gamma$ and $M_{eff}$. The $V_{mix}$ signals were measured under 4–10 GHz I_RF_ to determine $\tilde{D}$, $\left( {df}/{dH} \right)\left. \right|_{H_{ext}=H_{0}}$, and $\gamma$ values for all samples used in this study. All samples exhibited almost comparable values of $\gamma\approx\text{1.5}\text{–}\text{2} \times\text{10}^{\text{11}}rad\cdot s^{-1}T^{-1}$ and ${\mu_{0}M}_{eff}\approx$ 1.31 T. The $\sigma_{C}$ and $M_{s}t_{FM}$ values were obtained from Figure S7 and S8, respectively. The magnitude of the *I*_RF_ was calculated using $\text{I}_{\text{RF}}=\sqrt{2P/Z_{CPW}}$, where $P$ is the power absorbed by the CPW device (measured using a vector network analyzer), and $Z_{CPW}$ is the impedance of the CPW at a given *I*_RF_ frequency. To ensure optimal ST-FMR measurements, the CPW devices were designed to have $Z_{CPW}=\text{50 Ω}$. As a result, the magnitude of *I*_RF_ applied to all CPW devices in this study was estimated to be approximately 50–55 mA. Accordingly, $E$ was calculated using the equation: $E=\text{I}_{\text{RF}}Z_{CPW}/L$, where $L$ is the channel length of the CPW device. The term ${(dR_{d}}/{d\theta})\left. \right|_{\theta=45^{\circ}}$ corresponds to the anisotropic magnetoresistance (AMR) of the CPW device. AMR is a phenomenon in which the resistance of a ferromagnet varies with the angle between the applied current direction and the magnetization, and it serves as the primary source of the mixing voltage observed in ST-FMR measurements. To quantify AMR, an electric current was applied along the CPW channel while sweeping the angle $\theta$ between the current and the $H_{ext}$, and the angular dependence of the resistance $R_{d}(\theta)$ was recorded. In the presence of AMR, $R_{d}(\theta)$ follows the relation: $R_{d}(\theta)\propto\cos^{2} \theta$.^[23]^ By differentiating this curve, ${(dR_{d}}/{d\theta})\left. \right|_{\theta=45^{\circ}}$of CFB/Bi_1−_*_x_*Sb*_x_* (012)/Cr_1.6_Te_2_ was obtained at 300 K. In this study, the $V_{mix}$ signals measured at 6 GHz I_RF_ were primarily used to extract all the above parameters, from which we estimated $\theta_{\mathrm{SH}}$ and $\theta_{SH,z}$ for CFB/Bi_1−_*_x_*Sb*_x_* (012)/Cr_1.6_Te_2_ at 300 K.

Since CFB is employed as the ferromagnetic detector layer, which exhibits an AMR approximately one order of magnitude smaller than that of permalloy, a relatively large *I*_RF_ was required to obtain clear ST-FMR signals. However, when the input power exceeded 23 dBm, thermal effects were found to influence the $V_{mix}$ response. To eliminate such thermal artifacts, even at the expense of a slightly reduced signal amplitude, the nominal input power was fixed at 20 dBm for all ST-FMR measurements.

***SH-HV* *measurements*:** Second-harmonic Hall voltage (SH-HV) measurements can also be used to quantify the current-induced SOT efficiency. In this method, an AC current, $I\left( t \right)=I_{0}\sin\omega t$, is applied along the Hall bar device, and the transverse Hall voltage is detected at both the fundamental frequency $\omega$ and the second-harmonic frequency $2\omega$ using a conventional lock-in amplifier. The first-harmonic Hall voltage reflects the static Hall resistance of the device, whereas the second-harmonic Hall voltage originates from the current-induced oscillation of the magnetization and thermoelectric contributions. Since the SOT effective field is proportional to the applied current, it oscillates at $\omega$, modulates the Hall resistance at $\omega$, and consequently generates a voltage component at $2\omega$ through the mixing between the AC current and the oscillating Hall resistance. The first-harmonic Hall resistance ($R_{\omega,xy}$) is composed of the anomalous Hall effect (AHE) and the planar Hall effect (PHE): $R_{\omega,xy}=R_{\mathrm{AHE}}m_{z}+R_{\mathrm{PHE}}sin2\varphi+R_{\mathrm{offset}}$, where $R_{\mathrm{AHE}}$ is the anomalous Hall resistance, $R_{\mathrm{PHE}}$ is the planar Hall resistance, $m_{z}$ is the out-of-plane component of the magnetization, and $\varphi$ is the in-plane magnetization angle. Under a sufficiently large in-plane rotating magnetic field, the magnetization follows the field direction, and $R_{\omega,xy}$ is dominated by the PHE term, $R_{\omega,xy}(\varphi)=R_{\mathrm{PHE}}sin2\varphi+R_{\mathrm{offset}}$. $R_{PHE}$ can be determined from the angular dependence of $R_{\omega,xy}$. The second-harmonic Hall resistance ($R_{2\omega,xy}$) contains contributions from the damping-like torque, field-like torque, Oersted field, and thermoelectric effects. For an in-plane rotating magnetic field, the angular dependence of $R_{2\omega,xy}$ can be written as shown in Equation (S9).

$$R_{2\omega,xy}\left( \varphi\right)=\left( R_{\mathrm{AHE}}\frac{B_{DL,y}}{B_{\mathrm{eff}}}+R_{2\omega,\nabla T} \right)cos\varphi+2R_{\mathrm{PHE}}\frac{B_{FL,y}+B_{\mathrm{Oe}}}{B_{\mathrm{ext}}}\left( 2\cos^{3} \varphi-cos\varphi\right)-2R_{\mathrm{PHE}}\frac{B_{DL,z}}{B_{\mathrm{ext}}}cos2\varphi+R_{\mathrm{AHE}}\frac{B_{FL,z}}{B_{\mathrm{eff}}}$$

**(S9)**

Here, $B_{DL,y}$ ($B_{FL,y}$) is the $\boldsymbol{\sigma}_{\boldsymbol{y}}$-induced damping-like (field-like) torque field, whereas $B_{DL,z}$ ($B_{FL,z}$) is the $\boldsymbol{\sigma}_{\boldsymbol{z}}$-induced damping-like (field-like) torque field. $B_{\mathrm{Oe}}$ is the Oersted field, $B_{\mathrm{ex}t}$ is the externally applied in-plane magnetic field, and $B_{\mathrm{eff}}$ is the effective field. $R_{2\omega,\nabla T}$ represents the field-independent thermoelectric contribution, such as the anomalous Nernst or spin Seebeck effects. To extract the SOT efficiency, the $R_{2\omega,xy}\left( \varphi\right)$ for multiple external magnetic fields should be fitted using $R_{2\omega,xy}\left( \varphi\right)=R_{\left[ \cos(\varphi) \right]}cos\varphi+R_{\left[ 2cos^{3} (\varphi)-cos(\varphi) \right]}\left( 2\cos^{3} \varphi-cos\varphi\right)+R_{\left[ \cos(2\varphi) \right]}cos2\varphi+\mathrm{offset}$. In this expression, $R_{\left[ \cos(\varphi) \right]}$, $R_{\left[ 2cos^{3} (\varphi)-cos(\varphi) \right]}$, and $R_{\left[ \cos(2\varphi) \right]}$ represent the amplitudes of the corresponding angular components in $R_{2\omega,xy}\left( \varphi\right)$. The field dependence of the fitted coefficients can then be analyzed. $R_{\left[ \cos(\varphi) \right]}$ follows $R_{\left[ \cos(\varphi) \right]}=R_{\mathrm{AHE}}B_{DL,y}\left( \frac{1}{B_{\mathrm{eff}}} \right)+R_{2\omega,\nabla T}$. Therefore, $B_{DL,y}$ can be obtained from the slope of $R_{\left[ \cos(\varphi) \right]}/R_{\mathrm{AHE}}$ as a function of $\frac{1}{B_{\mathrm{eff}}}$. $R_{\left[ 2cos^{3} (\varphi)-cos(\varphi) \right]}$follows $R_{\left[ 2cos^{3} (\varphi)-cos(\varphi) \right]}=2R_{\mathrm{PHE}}(B_{FL,y}+B_{\mathrm{Oe}})(\frac{1}{B_{\mathrm{ext}}})$, and thus $B_{FL,y}+B_{\mathrm{Oe}}$ can be obtained from the slope of $R_{\left[ 2cos^{3} (\varphi)-cos(\varphi) \right]}/2R_{\mathrm{PHE}}$ as a function of $\frac{1}{B_{\mathrm{ext}}}$. $R_{\left[ \cos(2\varphi) \right]}$ follows $R_{\left[ \cos(2\varphi) \right]}=-2R_{PHE}B_{DL,z}\left( \frac{1}{B_{\mathrm{ext}}} \right)$. Therefore, $B_{DL,z}$ can be obtained from the slope of ${-R}_{\left[ \cos(2\varphi) \right]}/2R_{\mathrm{PHE}}$ as a function of $\frac{1}{B_{\mathrm{ext}}}$. Finally, $\theta_{SH,y}$ and $\theta_{SH,z}$ can be calculated using Equation (S10), where $B_{DL,i}$ is the corresponding torque field, and $J_{\mathrm{BiSb}}$ is the current density flowing through the Bi_1−_*_x_*Sb*_x_* layer.

$$\theta_{SH,i}=\frac{2e}{\hbar}\frac{M_{s}t_{\mathrm{FM}}B_{DL,i}}{J_{\mathrm{BiSb}}}$$

**(S10)**

**3.2	ST-FMR and SH-HV measurement data for Bi_1−_*_x_*Sb*_x_*/Cr_1.6_Te_2_**

**Figure S9.** ST-FMR signal of a) CFB 5 nm/Bi_0.9_Sb_0.1_ (012) 10 nm/Cr_1.6_Te_2_ 5 nm, and b) CFB 5 nm/Bi_0.8_Sb_0.2_ (012) 10 nm/Cr_1.6_Te_2_ 5 nm as a function of I_RF_ frequency (4–10 GHz) of 20 dBm. Fitting details of $V_{mix}$ signal of CFB 5 nm/Bi_0.9_Sb_0.1_ (012) 10 nm/Cr_1.6_Te_2_ 5 nm at I_RF_ of 6 GHz, 20 dBm applied along the c) *a*-axis, and d) *b*-axis.

**Figure S10.** Angular dependence of the a,c) $V_{S}$ and b,d) $V_{A}$ measured along the *a*-axis and *b*-axis, respectively, for CFB 5 nm/Bi_0.9_Sb_0.1_ (012) 10 nm/Cr_1.6_Te_2_ 5 nm. All of signals were measured at RT using *I*_RF_ of 6 GHz, 20 dBm.

**Figure S11.** Angular dependence of the a,c) $V_{S}$ and b,d) $V_{A}$ measured along the *a*-axis and *b*-axis, respectively, for CFB 5 nm/Bi_0.8_Sb_0.2_ (012) 10 nm/Cr_1.6_Te_2_ 5 nm. All signals were measured at RT using *I*_RF_ of 6 GHz, 20 dBm.

**Figure S12.** a) Fitting details of $V_{mix}$ signal of CFB 5 nm/Bi_0.7_Sb_0.3_ (012) 10 nm/Cr_1.6_Te_2_ 5 nm at *I*_RF_ of 6 GHz, 20 dBm. Angular dependence of the b) $V_{S}$ and c) $V_{A}$ for CFB 5 nm/Bi_0.7_Sb_0.3_ (012) 10 nm/Cr_1.6_Te_2_ 5 nm. All signals were measured at RT using *I*_RF_ of 6 GHz, 20 dBm.

**Figure S13.** a) Fitting details of $V_{mix}$ signal of CFB 5 nm/Bi_0.6_Sb_0.4_ (012) 10 nm/Cr_1.6_Te_2_ 5 nm at *I*_RF_ of 6 GHz, 20 dBm. Angular dependence of the b) $V_{S}$ and c) $V_{A}$ for CFB 5 nm/Bi_0.6_Sb_0.4_ (012) 10 nm/Cr_1.6_Te_2_ 5 nm. All signals were measured at RT using *I*_RF_ of 6 GHz, 20 dBm.

**Figure S14.** a) Fitting details of $V_{mix}$ signal of CFB 5 nm/Bi_0.4_Sb_0.6_ (012) 10 nm/Cr_1.6_Te_2_ 5 nm at *I*_RF_ of 6 GHz, 20 dBm. Angular dependence of the b) $V_{S}$ and c) $V_{A}$ for CFB 5 nm/Bi_0.4_Sb_0.6_ (012) 10 nm/Cr_1.6_Te_2_ 5 nm. All signals were measured at RT using *I*_RF_ of 6 GHz, 20 dBm.

**Figure S15.** a) Fitting details of $V_{mix}$ signal of CFB 5 nm/Bi_0.2_Sb_0.8_ (012) 10 nm/Cr_1.6_Te_2_ 5 nm at *I*_RF_ of 6 GHz, 20 dBm. Angular dependence of the b) $V_{S}$ and c) $V_{A}$ for CFB 5 nm/Bi_0.2_Sb_0.8_ (012) 10 nm/Cr_1.6_Te_2_ 5 nm. All signals were measured at RT using *I*_RF_ of 6 GHz, 20 dBm.

To extract $\theta_{\mathrm{SH}}$ and $\theta_{\mathrm{SH},z}$ using the $V_{S}$-only and $V_{A,z}$-only method, detailed information contained in the $V_{S}$ and $V_{A}$ components—such as the exact values of $\tilde{V_{S}}$ and $\tilde{V_{A}}$, $H_{0}$, and $\Delta H$—as well as $\tilde{D}$, $\gamma$, and $\left( {df}/{dH} \right)\left. \right|_{H_{ext}=H_{0}}$ values are required. To obtain these parameters, the $V_{mix}$ signals of CFB/Bi_1−_*_x_*Sb*_x_* (012)/Cr_1.6_Te_2_ were measured under 4–10 GHz I_RF_. Representative data for CFB 5 nm/Bi_0.9_Sb_0.1_ (012) 10 nm/Cr_1.6_Te_2_ 5 nm (*a*-axis) and CFB 5 nm/Bi_0.8_Sb_0.2_ (012) 10 nm/Cr_1.6_Te_2_ 5 nm (*b*-axis) as a function of I_RF_ frequency (4–10 GHz) are shown in Figure S9a,b. As shown in Figure S9c,d, the Lorentzian function fitting details of the $V_{mix}$ signals for CFB/Bi_0.9_Sb_0.1_ (012)/Cr_1.6_Te_2_ measured at 6 GHz I_RF_ along the *a*- and *b*-axes are presented according to the device geometry illustrated in Figure 2a (main manuscript). The CFB/Bi_0.9_Sb_0.1_ (012)/Cr_1.6_Te_2_ shows $V_{mix}\left( \boldsymbol{m} \right)\neq-V_{mix}(-\boldsymbol{m})$, with a particularly pronounced asymmetry in $\tilde{V_{A}}\left( \boldsymbol{m} \right)$ and $\tilde{V_{A}}(-\boldsymbol{m})$ only when the *I*_RF_ is applied along the *a*-axis. As shown in Figure S12a, S13a, S14a, S15a, the Lorentzian function fitting details of the $V_{mix}$ signals for CFB/Bi_1−_*_x_*Sb*_x_* (012)/Cr_1.6_Te_2_ with *x* = 0.3, 0.4, 0.6, and 0.8 with I_RF_ applied at $\varphi=-\text{90°}$ are also presented using the same device geometry as in Figure 2a (main manuscript). The CFB/Bi_1−_*_x_*Sb*_x_* (012)/Cr_1.6_Te_2_ with *x* = 0.3, 0.4, 0.6, and 0.8 exhibit small deviations in $V_{mix}$, i.e., $V_{mix}\left( \boldsymbol{m} \right)\neq-V_{mix}(-\boldsymbol{m})$, showing only slight differences in $\tilde{V_{A}}\left( \boldsymbol{m} \right)$ and $\tilde{V_{A}}(-\boldsymbol{m})$.

In addition, magnetic-field angle $\theta$-dependent ST-FMR measurements were performed to further confirm the presence of $\boldsymbol{\sigma}_{\boldsymbol{z}}$-induced SOTs. For each $\theta$, the measured $V_{\mathrm{mix}}(H)$ signal was fitted using the sum of symmetric and antisymmetric Lorentzian functions to extract $V_{S}$ and $V_{A}$. The resulting $V_{S}(\theta)$ and $V_{A}(\theta)$ were then fitted using the angular response functions $V_{S}\left( \theta\right)=Csin2\theta cos\theta$ and $V_{A}\left( \theta\right)=\mathrm{As}in2\theta cos\theta+\mathrm{Bs}in2\theta$, where C, A, and B are fitting parameters related to $\tau_{DL,y}$, $\tau_{FL,y}$, and $\tau_{DL,z}$, respectively. We also extracted $\text{V}_{DL,z}$ and $\text{V}_{FL+Oe,y}$ from the fitting of $V_{A}\left( \theta\right)$, where the uncertainties represent the standard errors obtained from the least-squares fitting. For CFB 5 nm/Bi_1−_*_x_*Sb*_x_* (012) 10 nm/Cr_1.6_Te_2_ 5 nm with *x* = 0.1–0.2, $V_{S}(\theta)$ and $V_{A}(\theta)$ were both extracted from the *a*-axis and *b*-axis. As shown in Figure S10 and S11, $V_{S}(\theta)$ for both the *a*- and *b*-axes is well reproduced by the expected angular dependence dominated by $\tau_{DL,y}$, whereas $V_{A}(\theta)$ measured along the *a*-axis requires both $\mathrm{As}in2\theta cos\theta$ and $\mathrm{Bs}in2\theta$ terms, revealing an additional $\tau_{DL,z}$ contribution in $V_{A}(\theta)$. By contrast, for $V_{A}(\theta)$ measured along the *b*-axis, the angular dependence is much more symmetric, and the additional term is strongly suppressed. For CFB 5 nm/Bi_1−_*_x_*Sb*_x_* (012) 10 nm/Cr_1.6_Te_2_ 5 nm with *x* = 0.3–0.8, $V_{S}(\theta)$ still follows the same overall symmetry, but the additional $\mathrm{Bs}in2\theta$ contribution in $V_{A}(\theta)$ becomes smaller than in the *x* = 0.1–0.2 samples. Although the asymmetric angular dependence is small, which is consistent with the fixed-angle ST-FMR measurement results, there is still an additional $\tau_{DL,z}$ contribution in $V_{A}(\theta)$. This comparison shows that the anomalous angular asymmetry is strongest in the single-domain-dominant samples and is substantially averaged out in the multi-domain samples, consistent with partial cancellation of the $\boldsymbol{\sigma}_{\boldsymbol{z}}$-induced torque contribution among differently oriented in-plane domains.

**Figure S16.** a) $\boldsymbol{\sigma}_{\boldsymbol{y}}$- and $\boldsymbol{\sigma}_{\boldsymbol{z}}$-induced spin Hall conductivity ($\sigma_{SH,y}$ and $\sigma_{SH, z}$) of data of Bi_1−_*_x_*Sb*_x_* (012) 10 nm/Cr_1.6_Te_2_ 5 nm as a function of *x*. b) $\theta_{\mathrm{SH}}$ of Bi_0.8_Sb_0.2_ (012) $t$nm/Cr_1.6_Te_2_ 5 nm with $t=$ 3, 5, 7, 10, 12, and 15. c) $\theta_{\mathrm{SH},z}$ of Bi_0.8_Sb_0.2_ (012) $t$nm/Cr_1.6_Te_2_ 5 nm with $t=$ 3, 5, 7, 10, 12, and 15. d) Spin canting angle Bi_0.8_Sb_0.2_ (012) $t$nm/Cr_1.6_Te_2_ 5 nm with $t=$ 3, 5, 7, 10, 12, and 15. The error bars represent the uncertainty associated with extracting $\theta_{\mathrm{SH}}$ and $\theta_{\mathrm{SH},z}$ values.

We extracted $\theta_{\mathrm{SH}}$ and $\theta_{\mathrm{SH},z}$ for CFB 5 nm/Bi_1−_*_x_*Sb*_x_* (012) 10 nm/Cr_1.6_Te_2_ 5 nm using the $V_{S}$-only and $V_{A,z}$-only methods, as shown in Figure 2d (main manuscript). Subsequently, the corresponding$\sigma_{SH,y}$ and $\sigma_{SH,z}$ values were calculated using $\sigma_{SH,y}={\frac{\hbar}{2e}\theta}_{\mathrm{SH}}\cdot\sigma_{C}$ and $\sigma_{SH,z}={\frac{\hbar}{2e}\theta}_{SH,z}\cdot\sigma_{C}$, as shown in Figure S16. The $\sigma_{C}$ values were taken from Figure S7. The percentage scale on the lower horizontal axis is defined as $({\sigma_{SH,z}/\sigma}_{SH,y})\times100\%$. For the single-domain CFB/Bi_1−_*_x_*Sb*_x_* (012)/Cr_1.6_Te_2_ structures (0.1 $\text{≤}$ *x* $\text{≤}$ 0.3), the highest $\sigma_{SH,y}$ and $\sigma_{SH,z}$ values are 4.55$\times\text{10}^{\text{5}}\frac{\hbar}{2e}\Omega^{-1}m^{-1}$ and 1.12$\times\text{10}^{\text{5}}\frac{\hbar}{2e}\Omega^{-1}m^{-1}$, respectively, obtained at *x* = 0.2. For the anisotropic three-fold twin-domain CFB/Bi_1−_*_x_*Sb*_x_* (012)/Cr_1.6_Te_2_ structures (0.4 $\text{≤}$ *x* $\text{≤}$ 0.8), the highest $\sigma_{SH,y}$ and $\sigma_{SH,z}$ values are 5.40$\times\text{10}^{\text{5}}\frac{\hbar}{2e}\Omega^{-1}m^{-1}$ and 0.511$\times\text{10}^{\text{5}}\frac{\hbar}{2e}\Omega^{-1}m^{-1}$, respectively, obtained at *x* = 0.6. The extracted $\theta_{\mathrm{SH}}$, $\theta_{\mathrm{SH},z}$, $\sigma_{SH,y}$, and $\sigma_{SH,z}$ values of CFB/Bi_1−_*_x_*Sb*_x_* (012)/Cr_1.6_Te_2_ as a function of Sb concentration are summarized in Table S2.

A recent study reported a large $\sigma_{SH,y}$ in Bi_1−_*_x_*Sb*_x_* (012) measured by ST-FMR, originating from the combined contributions of bulk states and topological surface states (TSS) to $\sigma_{SH,y}$. To elucidate the origins of $\boldsymbol{\sigma}_{\boldsymbol{y}}$ and $\boldsymbol{\sigma}_{\boldsymbol{z}}$ generation in single-domain Bi_0.8_Sb_0.2_/Cr_1.6_Te_2_, we investigated the thickness dependence of $\theta_{\mathrm{SH}}$ and $\theta_{\mathrm{SH},z}$. Figure S16b and S16c show the variations of $\theta_{\mathrm{SH}}$ and $\theta_{\mathrm{SH},z}$ of single-domain Bi_0.8_Sb_0.2_/Cr_1.6_Te_2_, respectively, as a function of the Bi_0.8_Sb_0.2_ thickness. The data were fitted using the conventional spin-diffusion model described by Equation (S11),^[14,18,24]^ where $A$ is the amplitude of tanh function, *x* is the thickness of Bi_0.8_Sb_0.2_, $x_{d}$ is the thickness of the dead layer, and $L_{s}$ is the spin-diffusion length.

| $A\tanh([x-x_{d}]/2L_{s})$ | **(S11)** |
| --- | --- |

If the SOT were dominated purely by bulk states, the in-plane spin Hall angle would increase with thickness at small $t$ and saturate when $t$ significantly exceeds $L_{s}$. As expected, the $\theta_{\mathrm{SH}}$—which contains contributions from both the bulk states and TSS—initially increases up to a thickness of approximately 7 nm and then decreases. This decrease arises because the relative contribution of TSS-induced SOT diminishes as the film becomes thicker. Consequently, as shown in Figure S16b, the conventional spin-diffusion model could not accurately fit the data, and the parameters obtained from the fitting are physically meaningless.

To further confirm that $\boldsymbol{\sigma}_{\boldsymbol{z}}$ originates from bulk carriers in single-domain Bi_0.8_Sb_0.2_/Cr_1.6_Te_2_, $\theta_{\mathrm{SH},z}$ was extracted for different thickness $t$ of Bi_0.8_Sb_0.2_ (012), as shown in Figure S16c. With increasing thickness, $\theta_{\mathrm{SH},z}$ increases sharply from $t=$ 3 nm to $t=$ 10 nm and then saturated. The $\theta_{\mathrm{SH},z}$ data are well fitted by the conventional spin-diffusion model, indicating that $\boldsymbol{\sigma}_{\boldsymbol{z}}$ is generated by bulk carriers.^[14,18,24]^ From the fitting, the fitting parameters of $L_{s}=$ 2.1 nm and $x_{d}=$ 3 nm were obtained, suggesting that Bi_0.8_Sb_0.2_ (012) begins to generate $\boldsymbol{\sigma}_{\boldsymbol{z}}$ when its thickness exceeds 3 nm—consistent with the strain-relaxation observed in Figure S1b. Finally, the spin-canting angle ($\beta$) was calculated using the equation^[18]^: $\beta=arctan(\frac{\theta_{\mathrm{SH},z}}{\theta_{\mathrm{SH}}})$, as shown in Figure S16d. $\beta$ increases with increasing Bi_0.8_Sb_0.2_ (012) thickness in the Bi_0.8_Sb_0.2_ (012)/Cr_1.6_Te_2_ 5 nm heterostructure, reaching approximately 25° for the 10 nm sample.

**Table S2. Summary of** $\boldsymbol{\theta}_{\mathbf{SH}}$**,** $\boldsymbol{\theta}_{\mathbf{SH}\boldsymbol{,z}}$**,** $\boldsymbol{\sigma}_{\mathbf{SH,}\boldsymbol{y}}$**, and** $\boldsymbol{\sigma}_{\mathbf{SH,}\boldsymbol{z}}$ **for CFB/Bi_1−_*_x_*Sb*_x_* (012)/Cr_1.6_Te_2_ at 300 K.**

| Samples | $\theta_{\mathrm{SH}}$ | $\theta_{\mathrm{SH},z}$ | $\sigma_{SH,y}$ (300 K) [$\frac{\hbar}{2e}$10^5^$\Omega^{-1}m^{-1}$] | $\sigma_{SH,z}$ (300 K) [$\frac{\hbar}{2e}$10^5^$\Omega^{-1}m^{-1}$] |
| --- | --- | --- | --- | --- |
| $x$ = 0.1 | 0.922 | 0.161 | 2.19 | 0.383 |
| $x$ = 0.2 | 1.68 | 0.417 | 4.55 | 1.12 |
| $x$= 0.3 | 1.46 | 0.157 | 4.38 | 0.469 |
| $x$ = 0.4 | 1.20 | 0.0376 | 4.45 | 0.139 |
| $x$ = 0.6 | 1.11 | 0.105 | 5.40 | 0.511 |
| $x$ = 0.8 | 0.956 | 0.0552 | 4.60 | 0.318 |

**Figure S17.** a) $R_{\omega,xy}$ of Bi_0.8_Sb_0.2_ (012) 10 nm/Cr_1.6_Te_2_ 5 nm measured as a function of the in-plane magnetic-field angle $\varphi$ at 150 K under $B_{\mathrm{ext}}=$ 3 T. Angular dependence of the $R_{2\omega,xy}$ measured along b) the *a*-axis and c) the *b*-axis of Bi_0.8_Sb_0.2_ under in-plane magnetic fields of 2, 2.5, and 3 T. $R_{\mathrm{residual}}$ of the angular fitting for $R_{2\omega,xy}$ measured under in-plane magnetic fields of d) 2 T, e) 2.5 T, and f) 3 T for the *a*- and *b*-axes. The *b*-axis residuals are vertically offset for clarity. g) Linear fitting of the $R_{\left[ \cos(\varphi) \right]}/R_{\mathrm{AHE}}$ as a function of 1/$B_{\mathrm{eff}}$ for the *a*-axis. h) Linear fitting of $R_{\left[ 2cos^{3} (\varphi)-cos(\varphi) \right]}/{2R}_{\mathrm{PHE}}$ as a function of 1/$B_{\mathrm{ext}}$ for the *a*-axis. i) Linear fitting of $R_{\left[ \cos(2\varphi) \right]}/2R_{\mathrm{PHE}}$ as a function of 1/$B_{\mathrm{ext}}$ for the *a*-axis. Error bars in (g)–(i) represent the standard errors of the fitted angular coefficients. The current density $J_{\mathrm{BiSb}}=\text{6.13×}\text{10}^{\text{5}}\text{ A/}\text{cm}^{\text{2}}$ was used for all measurements.

To further confirm the existence of $\boldsymbol{\sigma}_{\boldsymbol{z}}$ under the same temperature conditions as the magnetization switching measurements, we performed SH-HV measurements on the Bi_0.8_Sb_0.2_ (012)/Cr_1.6_Te_2_ device at 150 K. Although the presence of $\boldsymbol{\sigma}_{\boldsymbol{z}}$**-**induced SOT was confirmed by ST-FMR measurements at room temperature, the ST-FMR measurements were performed using a CFB/Bi_1−_*_x_*Sb*_x_* interface, whereas the current-induced magnetization switching was demonstrated at the Bi_1−_*_x_*Sb*_x_*/Cr_1.6_Te_2_ interface at 150 K. Therefore, SH-HV measurements were carried out to directly examine the magnitude of $\boldsymbol{\sigma}_{\boldsymbol{z}}$**-**induced SOT in the Bi_1−_*_x_*Sb*_x_*/Cr_1.6_Te_2_ switching structure at 150 K.

To conduct SH-HV measurements, an AC current with a peak amplitude of 1.3 mA was applied along a 10 μm wide Hall bar device. Because the lock-in voltage is measured in root-mean-square (RMS) units, the RMS current density flowing through Bi_0.8_Sb_0.2_ (012) corresponds to $6.13\times{10}^{5} A/\mathrm{cm}^{2}$. First, $R_{\omega,xy}$ was measured under an in-plane rotating magnetic field of 3 T at 150 K to determine $R_{\mathrm{PHE}}$, as shown in Figure S17a. From the $sin2\varphi$ angular dependence, $R_{\mathrm{PHE}}=0.038 \Omega$ was obtained and used for the analysis of $R_{2\omega,xy}$. Figure S17b and S17c show the angular dependence of $R_{2\omega,xy}$ measured along the *a*- and *b*-axes, respectively, under in-plane magnetic fields of 2, 2.5, and 3 T. For $R_{2\omega,xy}$ measured along the *b*-axis, there was a negligible $cos2\varphi$ angular component in $R_{2\omega,xy}$, indicating that $\boldsymbol{\sigma}_{\boldsymbol{z}}$**-**induced SOT is not generated, consistent with our ST-FMR and magnetization switching measurements. However, $R_{2\omega,xy}$ measured along the *a*-axis required $R_{\left[ \cos(2\varphi) \right]}$ to fit the $R_{2\omega,xy}$ data. To further evaluate the reliability of the $\boldsymbol{\sigma}_{\boldsymbol{z}}$**-**induced SOT obtained from the angular fitting, we calculated the residual signal after subtracting the non-$\boldsymbol{\sigma}_{\boldsymbol{z}}$-related angular components from each $R_{2\omega,xy}$ curve as $R_{\mathrm{residual}}=R_{2\omega,xy}-{[R}_{\left[ \cos(\varphi) \right]}+R_{\left[ 2cos^{3} (\varphi)-cos(\varphi) \right]}]$. The $R_{\mathrm{residual}}$ signals obtained under $B_{\mathrm{ext}}=$ 2, 2.5, and 3 T are shown in Figure S17d–f for both the *a*- and *b*-axes. After subtracting the fitted angular components ($R_{\left[ \cos(\varphi) \right]}+R_{\left[ 2cos^{3} (\varphi)-cos(\varphi) \right]}$), the residuals remain small compared with the total $R_{2\omega,xy}$ signals but exhibit a systematic $\cos2\varphi$-like dependence only for the *a*-axis data, indicating the existence of $\boldsymbol{\sigma}_{\boldsymbol{z}}$**-**induced SOT only when the current is applied along the *a*-axis.

The extracted $R_{\left[ \cos(\varphi) \right]}$, $R_{\left[ 2cos^{3} (\varphi)-cos(\varphi) \right]}$, and $R_{\left[ \cos(2\varphi) \right]}$ coefficients for the *a*-axis were then plotted as a function of 1/$B_{\mathrm{eff}}$ or 1/$B_{\mathrm{ext}}$, as shown in Figure S17g–i. The linear fitting of $R_{\left[ \cos(\varphi) \right]}/R_{\mathrm{AHE}}$ as a function of 1/$B_{\mathrm{eff}}$ yielded a slope corresponding to 3.93 ± 0.229 mT, indicating that $B_{DL,y}=\text{3.93 ± 0.229 }\text{mT}$. Using $M_{S}=\text{176 emu/}\text{cm}^{\text{-3}}$, obtained from Figure S18, $t_{\mathrm{FM}}=\text{5 nm}$, and $J_{\mathrm{BiSb}}=\text{6.13×}\text{10}^{\text{5}}\text{ A/}\text{cm}^{\text{2}}$, $\theta_{\mathrm{SH}}=\text{1.72 }\text{± 0.100}$ can be estimated using Equation (S10). The value of $\theta_{\mathrm{SH}}=\text{1.72 ± 0.100}$ at 150 K is slightly larger than the RT value ($\theta_{\mathrm{SH}}=\text{1.68}$) measured by ST-FMR. The linear fitting of $R_{\left[ 2cos^{3} (\varphi)-cos(\varphi) \right]}/{2R}_{\mathrm{PHE}}$ as a function of 1/$B_{\mathrm{ext}}$ gives $B_{FL,y}+B_{\mathrm{Oe}}=-\text{0.828 }\text{± 0.0753} \text{mT}$. Finally, $B_{DL,z}$ was extracted from the linear fitting of $R_{\left[ \cos(2\varphi) \right]}/2R_{\mathrm{PHE}}$ as a function of 1/$B_{\mathrm{ext}}$. Since $\frac{R_{\left[ \cos(2\varphi) \right]}}{2R_{\mathrm{PHE}}}=-B_{DL,z}(\frac{1}{B_{\mathrm{ext}}})$, the negative slope gives $B_{DL,z}$: $B_{DL,z}=\text{0.626 }\text{± 0.0318}\text{ }\text{mT}$. The corresponding $\theta_{SH,z}$ at 150 K was calculated as $\theta_{SH,z}=\text{0.2}\text{73}\text{ }\text{± 0.}\text{0}\text{139}$, which is smaller than the RT value ($\theta_{SH,z}=\text{0.417}$) measured by ST-FMR. This difference may originate from several factors: 1) the spin transmission efficiency can differ because the ferromagnetic interface is changed from CFB/Bi_1−_*_x_*Sb*_x_* to Bi_1−_*_x_*Sb*_x_*/Cr_1.6_Te_2_; and 2) the amount of $\boldsymbol{\sigma}_{\boldsymbol{z}}$**-**induced SOT generated in Bi_1−_*_x_*Sb*_x_* (012) may itself decrease as the temperature is reduced from RT to 150 K. Nevertheless, the observation of finite $\boldsymbol{\sigma}_{\boldsymbol{z}}$**-**induced SOT under both measurement conditions indicates that $\boldsymbol{\sigma}_{\boldsymbol{z}}$ is robustly generated in Bi_1−_*_x_*Sb*_x_* (012), even when the temperature and ferromagnetic interface are changed. Thus, SH-HV measurements at 150 K confirm that a finite $\boldsymbol{\sigma}_{\boldsymbol{z}}$**-**induced SOT is also generated in the Bi_0.8_Sb_0.2_ (012)/Cr_1.6_Te_2_ switching structure.

However, at this stage, we cannot conclusively determine whether a topological surface state (TSS)-originated $\boldsymbol{\sigma}_{\boldsymbol{z}}$**-**induced SOT exists in Bi_1−_*_x_*Sb*_x_*. Unlike $\boldsymbol{\sigma}_{\boldsymbol{y}}$**-**induced SOT, TSSs generally possess only in-plane spin components in the absence of hexagonal warping; even with hexagonal warping, accumulation of $\boldsymbol{\sigma}_{\boldsymbol{z}}$ is not expected in the ideal case. Although our ST-FMR and magnetization switching results indicate a strong bulk-origin $\boldsymbol{\sigma}_{\boldsymbol{z}}$ contribution, further investigation of possible TSS-induced out-of-plane SOT would be an important direction for future study.

**Supplementary Note S4. Current-induced magnetization switching in single-domain Bi_1−_*_x_*Sb*_x_* (012)/Cr_1.6_Te_2_**

**Figure S18.** a) SQUID measurement data of Bi_0.8_Sb_0.2_ (012) 10 nm/Cr_1.6_Te_2_ 5 nm. Field-cooling (FC) measurement was performed under an applied magnetic field of 100 Oe parallel to the *c*-axis. b) *M–H* curves of Bi_1−_*_x_*Sb*_x_* (012) 10 nm/Cr_1.6_Te_2_ 5 nm as a function of Sb concentration measured at 150 K for out-of-plane (OOP, *x* = 0.2) and in-plane (IP, *x* = 0.1–0.8) field directions.

Superconducting quantum interference device (SQUID) measurements were performed to investigate the magnetic properties of Cr_1.6_Te_2_ in the Bi_1−_*_x_*Sb*_x_* (012) 10 nm/Cr_1.6_Te_2_ 5 nm heterostructure. As shown in Figure S18a, the out-of-plane magnetization was measured under a 100 Oe magnetic field applied parallel to the *c*-axis during the field-cooling (FC) process. The FC curve shows a gradual decrease in magnetization with increasing temperature, confirming that the Curie temperature of Bi_0.8_Sb_0.2_ (012)/Cr_1.6_Te_2_ is approximately 220 K. The magnetization of Bi_0.8_Sb_0.2_ (012)/Cr_1.6_Te_2_ decreases sharply when the temperature rises above 150 K. As shown in Figure S18b, the Bi_1−_*_x_*Sb*_x_* (012) 10 nm/Cr_1.6_Te_2_ 5 nm heterostructure exhibits perpendicular magnetic anisotropy (PMA), consistent with the magnetization versus magnetic field (*M*–*H*) curve shown in the inset of Figure 3d (main manuscript). The effective anisotropy field ($H_{k,\mathrm{eff}}$) and $M_{S}$ of the Bi_1−_*_x_*Sb*_x_* (012) 10 nm/Cr_1.6_Te_2_ 5 nm heterostructure remain nearly identical across different Sb concentrations, although the coercivity (*H*_C_) and Curie temperature (*T*_C_) can be enhanced by the proximity effect due to the strong spin–orbit coupling (SOC) of Bi_1−_*_x_*Sb*_x_*.

In the Cr_1.6_Te_2_ system, decreasing the thickness also significantly reduces the anomalous Hall readout, making the electrical detection less reliable. For example, in the Bi_0.8_Sb_0.2_ (012)/Cr_1.6_Te_2_ heterostructure, reducing the Cr_1.6_Te_2_ thickness from 5 nm to 3 nm leads to a pronounced decrease in anomalous Hall resistance. This issue becomes even more severe in Sb-rich samples, such as Bi_0.2_Sb_0.8_, because the higher conductivity of the Bi_1−_*_x_*Sb*_x_* layer further suppresses the measurable anomalous Hall signal from the Cr_1.6_Te_2_ layer. For instance, when field-assisted magnetization switching is measured in Bi_0.2_Sb_0.8_ (012) 10 nm/Cr_1.6_Te_2_ 3 nm, the reliability of the anomalous Hall signal becomes significantly reduced. However, when Cr_1.6_Te_2_ 5 nm is used, both the field-free and field-assisted switching ratios are reduced compared with those obtained with Cr_1.6_Te_2_ 3 nm, owing to the reduced effective SOT acting per unit magnetic moment. Therefore, the 5 nm thickness was selected as a practical compromise between (1) switching efficiency and switching ratio and (2) measurement reliability.

**4.1	Determination of *J*_C_** **using the parallel-resistor model**

To determine the critical switching current density (*J*_C_) for current-induced magnetization switching measurements of Bi_1−_*_x_*Sb*_x_* (012) 10 nm/Cr_1.6_Te_2_ 5 nm at 150 K, we employed the parallel-resistor model.^[17]^ Using the measured critical switching current (*I*_C_) and the resistivity values of Cr_1.6_Te_2_ 5 nm ($\rho_{\mathrm{CrTe}}$) and Bi_1−_*_x_*Sb*_x_* (012) 10 nm ($\rho_{\mathrm{BiSb}}$) at 150 K, obtained from Figure S5 and S7, respectively, *J*_C_ was calculated using Equation (S12). The $\rho_{\mathrm{CrTe}}$ at 150 K is 348$\times{10}^{-6} \Omega\cdot cm$.

$$\text{J}_{\text{C}}=\text{I}_{\text{C}}\frac{\frac{\rho_{\mathrm{CrTe}}}{t_{\mathrm{CrTe}}}}{\frac{\rho_{\mathrm{CrTe}}}{t_{\mathrm{CrTe}}}+\frac{\rho_{\mathrm{BiSb}}}{t_{\mathrm{BiSb}}}}\times\frac{1}{w\times t_{\mathrm{BiSb}}}$$

**(S12)**

Here, $t_{\mathrm{CrTe}}$ and $t_{B\mathrm{iSb}}$ represent the thicknesses of each layer, and $w$ is the width of the Hall bar device channel ($w=$ 20 μm in this study).

**4.2	Current-induced magnetization switching in** **Bi_1−_*_x_*Sb*_x_* (012) with 0.1** $\text{≤}$ ***x*** $\text{≤}$ **0.3**

**Figure S19.** a) AHE loop of Bi_0.9_Sb_0.1_ (012) 10 nm/Cr_1.6_Te_2_ 5 nm at 150 K. Current-induced magnetization switching of Bi_0.9_Sb_0.1_ (012) 10 nm/Cr_1.6_Te_2_ 5 nm at 150 K under various in-plane magnetic fields and in the absence of a magnetic field, measured along the b) *a*-axis, and **c** *b*-axis, respectively.

Figure S19a shows a clear anomalous Hall effect (AHE) loop, confirming the presence of PMA in Bi_0.9_Sb_0.1_ (012) 10 nm/Cr_1.6_Te_2_ 5 nm at 150 K, with an AHE resistance of 1.36 $\Omega$. As shown in Figure S19b,c, Bi_0.9_Sb_0.1_ (012) 10 nm/Cr_1.6_Te_2_ 5 nm exhibits field-free magnetization switching only when the current is applied along the *a*-axis of Bi_0.9_Sb_0.1_ (012). For the *J*_C_, the Bi_0.9_Sb_0.1_ (012) 10 nm/Cr_1.6_Te_2_ 5 nm (*a*-axis) shows *J*_C_ of approximately 1.00×10^6^ A/cm^2^ without in-plane external magnetic field ($\boldsymbol{B}_{\boldsymbol{x}}$) at 150 K. As expected, a reversal of the switching polarity under opposite $\boldsymbol{B}_{\boldsymbol{x}}$ polarity is observed only when the current is applied along the *b*-axis (Figure S19c). The $\boldsymbol{B}_{\boldsymbol{x}}$-assisted magnetization switching data of Bi_0.9_Sb_0.1_ (012) 10 nm/Cr_1.6_Te_2_ 5 nm (*a*-axis) indicate a compensation field (*H*_comp_) of −22 mT at 150 K. Compared with the single-domain Bi_0.8_Sb_0.2_ (012) 10 nm/Cr_1.6_Te_2_ 5 nm heterostructure, this value is slightly smaller, which can be attributed to the weaker out-of-plane SOT generated in Bi_0.9_Sb_0.1_ (012) 10 nm/Cr_1.6_Te_2_ 5 nm, as confirmed by the ST-FMR results in Figure 2d (main manuscript). The switching ratio for field free magnetization switching of Bi_0.9_Sb_0.1_ (012) 10 nm/Cr_1.6_Te_2_ 5 nm (*a*-axis) at 150 K is approximately 44 %, whereas the switching ratios for $\boldsymbol{B}_{\boldsymbol{x}}$-assisted magnetization switching at 150 K are approximately 81.8% (*a*-axis, 50 mT), and 79.8 % for (*b*-axis, 50 mT), respectively.

**Figure S20.** a) Current-induced magnetization switching of Bi_0.8_Sb_0.2_ (012) 10 nm/Cr_1.6_Te_2_ 5 nm under in-plane magnetic fields of $\pm$50 mT and in the absence of a magnetic field, measured along the *b*-axis. b) Current-induced magnetization switching of Bi_0.8_Sb_0.2_ (012) 10 nm/Cr_1.6_Te_2_ 5 nm under an in-plane magnetic field of 50 mT with current pulse widths of 100 μs, 50 μs, and 12 μs. c) Current-induced field-free magnetization switching of Bi_0.8_Sb_0.2_ (012) 10 nm/Cr_1.6_Te_2_ 5 nm with current pulse widths of 100 μs, 50 μs, and 12 μs.

Figure S20a provides the current-induced magnetization switching of Bi_0.8_Sb_0.2_ (012) 10 nm/Cr_1.6_Te_2_ 5 nm under $\boldsymbol{B}_{\boldsymbol{x}}$ of $\pm$50 mT and in the absence of a magnetic field, measured along the *b*-axis at 150 K. As expected, field-free magnetization switching was not observed. For the *J*_C_, Bi_0.8_Sb_0.2_ (012) 10 nm/Cr_1.6_Te_2_ 5 nm (*b*-axis) shows *J*_C_ of approximately 1.03×10^6^ A/cm^2^ under $\boldsymbol{B}_{\boldsymbol{x}}$ of 50 mT at 150 K. This value is slightly larger than that of Bi_0.8_Sb_0.2_ (012) 10 nm/Cr_1.6_Te_2_ 5 nm (*a*-axis), 0.815×10^6^ A/cm^2^ (150 K), which is attributed to the absence of $\boldsymbol{\sigma}_{\boldsymbol{z}}$. This clearly demonstrates that $\boldsymbol{\sigma}_{\boldsymbol{z}}$-induced SOTs effectively reduce *J*_C_. The switching ratio for field-free magnetization switching of Bi_0.8_Sb_0.2_ (012) 10 nm/Cr_1.6_Te_2_ 5 nm (*a*-axis) at 150 K is approximately 52.8 %, while the switching ratios for $\boldsymbol{B}_{\boldsymbol{x}}$-assisted magnetization switching at 150 K are approximately 86.7% (*a*-axis, 50 mT), and 81.1 % (*b*-axis, 50 mT), respectively. We also examined the dependence of magnetization switching on pulse width at 150 K, as shown in Figure S20b ($\boldsymbol{B}_{\boldsymbol{x}}$-assisted) and S20c (field-free). In both cases, robust SOT switching was maintained down to the pulse-width limit of 12 μs, set by the Keithley 6221.

However, because a relatively thick Cr_1.6_Te_2_ layer was used in this study for detection reliability, reducing the pulse width can make the SOT and the effective symmetry-breaking field generated by out-of-plane spins insufficient for fully deterministic magnetization switching (under field-free conditions). In general, reducing the ferromagnetic thickness can improve the effective SOT acting on the magnetization, because the same spin current is distributed over a smaller magnetic volume, resulting in a reduction in *J*_C_ and an increase in the switching ratio. Under short-pulse conditions, reverse domains may nucleate, but their subsequent propagation across the Hall channel may remain incomplete before the pulse ends. As a result, a portion of the multi-domain state can remain unreversed, which directly lowers the switching ratio, as shown in Figure S20b and S20c. Importantly, if thermal effects induced by the pulsed current played a dominant role in the magnetization switching, the field-free switching data shown in Figure S19, S20, and S22, where the current was applied along the *b*-axis, should also exhibit switching loops. However, such behavior was not observed. Therefore, we interpret the markedly reduced switching ratio at 12 μs not as evidence of dominant Joule heating, but rather as a consequence of incomplete domain-mediated switching in the thicker Cr_1.6_Te_2_ devices.

Moreover, the relatively low field-free switching ratio is not mainly caused by geometric effects or experimental artifacts, such as those associated with the transverse voltage probes. Otherwise, a similarly reduced switching ratio would be expected for both field-free and field-assisted switching measured in the same device geometry. However, in our devices, field-assisted current-induced switching exhibits a much larger switching ratio than field-free switching in the same Hall-bar geometry, indicating that the Hall-bar geometry itself is not the origin of the reduced field-free switching ratio. Thus, while possible device-geometry-related effects, such as current nonuniformity and domain pinning near the transverse voltage probes, may contribute to the incomplete switching ratio, they cannot be the dominant origin of the reduced field-free switching ratio.

In addition, the broadened Hall response observed in our devices can be understood in terms of incomplete and less deterministic multi-domain switching under zero-field, short-pulse conditions. This interpretation is further supported by the clear difference between field-assisted and field-free magnetization switching data (Figure S20b and S20c). For the same pulse width, the Hall signal broadening is substantially reduced when an assisting in-plane magnetic field is applied. If Joule heating were the dominant origin of the magnetization switching and of the reduced broadening observed at longer pulse widths, such a pronounced difference would not be expected solely from the presence or absence of the in-plane field under otherwise identical pulse conditions. Instead, this result indicates that the in-plane field promotes more deterministic switching in the thick Cr_1.6_Te_2_ system by helping the system overcome the effective switching barrier and by facilitating reverse-domain nucleation, domain-wall depinning, and propagation against the effective interfacial Dzyaloshinskii–Moriya interaction field. In this situation, the Hall response under shorter current pulses can appear broadened or irregular because the measured anomalous Hall resistance reflects an average over mixed up- and down-magnetized domains, rather than two fully stabilized magnetic states. Therefore, the reduction in Hall signal broadening at longer pulse width is not evidence of a dominant contribution from increased thermal effects. A quantitative evaluation of thermal effects in magnetization switching is addressed in Supplementary Note S6.1. Nevertheless, the AHE loop does not disappear completely, suggesting that, by employing a PMA ferromagnet with larger magnetization, current-induced magnetization switching could be observed even at shorter pulse widths.

**Figure S21.** a) Resistivity of Bi_0.8_Sb_0.2_ (012) $t$ nm extracted from Bi_0.8_Sb_0.2_ (012) $t$ nm/Cr_1.6_Te_2_ 5 nm with $t=$ 3, 5, 7, and 12 using the parallel-resistor model. b) Current-induced magnetization switching of Bi_0.8_Sb_0.2_ (012) 3 nm/Cr_1.6_Te_2_ 5 nm under in-plane magnetic fields of 50 mT and in the absence of a magnetic field, measured along the *a*-axis. Current-induced magnetization switching of Bi_0.8_Sb_0.2_ (012) $t$ nm/Cr_1.6_Te_2_ 5 nm under an in-plane magnetic fields of *H*_comp_ and in the absence of a magnetic field, measured along the *a*-axis for c) $t=$ 5, d) $t=$ 7, and e) $t=$ 12, respectively. f) *J*_C_ of Bi_0.8_Sb_0.2_ (012) $t$ nm/Cr_1.6_Te_2_ 5 nm with $t=$ 3, 5, 7, 10, and 12.

Figure S21 presents the current-induced magnetization switching data of Bi_0.8_Sb_0.2_ (012) $t$ nm/Cr_1.6_Te_2_ 5 nm (*a*-axis) with $t=$ 3, 5, 7, and 12, corresponding to Figure 3f (main manuscript). First, we measured the resistivity of Bi_0.8_Sb_0.2_ (012) $t$ nm/Cr_1.6_Te_2_ 5 nm with $t=$ 3, 5, 7, 10, and 12, and extracted the resistivity of Bi_0.8_Sb_0.2_ (012) $t$ nm using the parallel-resistor model, as shown in Figure S21a. The resistivity of Bi_0.8_Sb_0.2_ (012) increases sharply when $t<$ 7, reaching approximately 710 $\mu\Omega\mathrm{cm}$ at $t=$ 3. For Bi_0.8_Sb_0.2_ (012) 3 nm/Cr_1.6_Te_2_ 5 nm, field-free magnetization switching was not observed, consistent with the absence of out-of-plane SOT in the ST-FMR data (Figure S16c). In contrast, Bi_0.8_Sb_0.2_ (012) $t$ nm/Cr_1.6_Te_2_ 5 nm with $t=$ 3, 5, 7, 10, and 12 exhibit both field-free magnetization switching and *H*_comp_, as shown in Figure S21b–d. For the *J*_C_, Bi_0.8_Sb_0.2_ (012) $t$ nm/Cr_1.6_Te_2_ 5 nm (*a*-axis) with $t=$ 5, 7, and 12 shows *J*_C_ of approximately 2.91×10^6^ A/cm^2^, 1.06×10^6^ A/cm^2^, and 0.89×10^6^ A/cm^2^, respectively, without $\boldsymbol{B}_{\boldsymbol{x}}$ at 150 K. For Bi_0.8_Sb_0.2_ (012) 3 nm/Cr_1.6_Te_2_ 5 nm (*a*-axis), *J*_C_ of 5.07×10^6^ A/cm^2^ was obtained under $\boldsymbol{B}_{\boldsymbol{x}}$ of 50 mT at 150. The corresponding *H*_comp_ values for Bi_0.8_Sb_0.2_ (012) $t$ nm/Cr_1.6_Te_2_ 5 nm (*a*-axis) with $t=$ 5, 7, and 12 are of approximately −8 mT, −32 mT, and −45 mT, respectively. The *J*_C_ values for Bi_0.8_Sb_0.2_ (012) $t$ nm/Cr_1.6_Te_2_ 5 nm with $t=$ 3, 5, 7, 10 and 12 are summarized in Figure S21f.

**Figure S22.** a) AHE loop of Bi_0.7_Sb_0.3_ (012) 10 nm/Cr_1.6_Te_2_ 5 nm at 150 K. Current-induced magnetization switching of Bi_0.7_Sb_0.3_ (012) 10 nm/Cr_1.6_Te_2_ 5 nm at 150 K under various in-plane magnetic fields and in the absence of a magnetic field, measured along the b) *a*-axis, and c) *b*-axis, respectively.

Figure S22a shows a clear AHE loop, confirming the presence of PMA in Bi_0.7_Sb_0.3_ (012) 10 nm/Cr_1.6_Te_2_ 5 nm at 150 K, with an AHE resistance of 0.61 $\Omega$. As shown in Figure S22b,c, Bi_0.7_Sb_0.3_ (012) 10 nm/Cr_1.6_Te_2_ 5 nm exhibits field-free magnetization switching only when the current is applied along the *a*-axis of Bi_0.7_Sb_0.3_ (012). For the *J*_C_, Bi_0.7_Sb_0.3_ (012) 10 nm/Cr_1.6_Te_2_ 5 nm (*a*-axis) shows *J*_C_ of approximately 0.96×10^6^ A/cm^2^ without $\boldsymbol{B}_{\boldsymbol{x}}$ at 150 K. As expected, a reversal of the switching polarity under opposite $\boldsymbol{B}_{\boldsymbol{x}}$ polarity is observed only when the current is applied along the *b*-axis (Figure S22c). The $\boldsymbol{B}_{\boldsymbol{x}}$-assisted magnetization switching data of Bi_0.7_Sb_0.3_ (012) 10 nm/Cr_1.6_Te_2_ 5 nm (*a*-axis) indicates a *H*_comp_ of −27 mT at 150 K. Compared with the single-domain Bi_0.8_Sb_0.2_ (012) 10 nm/Cr_1.6_Te_2_ 5 nm heterostructure, this value is slightly smaller, which can be attributed to the weaker out-of-plane SOT generated in Bi_0.7_Sb_0.3_ (012) 10 nm/Cr_1.6_Te_2_ 5 nm, as confirmed by the ST-FMR results in Figure 2d (main manuscript). The switching ratio for field free magnetization switching of Bi_0.7_Sb_0.3_ (012) 10 nm/Cr_1.6_Te_2_ 5 nm (*a*-axis) at 150 K is approximately 42 %, while the switching ratios for $\boldsymbol{B}_{\boldsymbol{x}}$-assisted magnetization switching at 150 K are approximately 81.0% (*a*-axis, 50 mT), and 80.4 % for (*b*-axis, 50 mT), respectively.

**4.3	Current-induced magnetization switching in Bi_1−_*_x_*Sb*_x_* (012)/[Co/Pt]****Figure S23.** a) $\varphi$-scan spectra of Bi_1−_*_x_*Sb*_x_* {202} for Bi_0.8_Sb_0.2_ (012) 10 nm/[Co (0.6 nm)/Pt (0.4 nm)]_4_. b) VSM data of Bi_1−_*_x_*Sb*_x_* (012) 10 nm/[Co (0.6 nm)/Pt (0.4 nm)]_4_ measured under an out-of-plane magnetic field. c) Current-induced magnetization switching data of Bi_1−_*_x_*Sb*_x_* (012) 10 nm/[Co (0.6 nm)/Pt (0.4 nm)]_4_ under an in-plane magnetic field of $\pm$50 mT at 300 K.

To verify the in-plane ordering of Bi_0.8_Sb_0.2_ (012) grown on the [Co (0.6 nm)/Pt (0.4 nm)]_4_ layer, $\varphi$-scan measurements were performed using the Bi_0.8_Sb_0.2_ {202} reflections, as shown in Figure S23a. The Bi_0.8_Sb_0.2_ (012)/[Co (0.6 nm)/Pt (0.4 nm)]_4_ exhibits no distinct $\varphi$ peaks, indicating the absence of in-plane ordering in this sample. Therefore, field-free magnetization switching is not expected for current applied along any in-plane directions. Figure S23b shows a clear square-shaped magnetization–magnetic field (M–H) loop, confirming the presence of PMA in Bi_0.8_Sb_0.2_ (012) 10 nm/[Co (0.6 nm)/Pt (0.4 nm)]_4_ at 300 K. Accordingly, we measured the current-induced magnetization switching of Bi_0.8_Sb_0.2_ (012) 10 nm/[Co (0.6 nm)/Pt (0.4 nm)]_4_ at 300 K. The Hall bar channel was aligned at $\varphi=$ 90°, following the device geometry shown in Figure 3c (main manuscript). For Bi_0.8_Sb_0.2_ (012) 10 nm/[Co (0.6 nm)/Pt (0.4 nm)]_4_, field-free magnetization switching was not observed due to the absence of in-plane ordering in Bi_0.8_Sb_0.2_ (012), and therefore no $\boldsymbol{\sigma}_{\boldsymbol{z}}$ was generated. The *J*_C_ of Bi_0.8_Sb_0.2_ (012) 10 nm/[Co (0.6 nm)/Pt (0.4 nm)]_4_ was approximately 2.47×10^6^ A/cm^2^ under $\boldsymbol{B}_{\boldsymbol{x}}\boldsymbol{=}$ 50 mT at 300 K. This *J*_C_ value is significantly larger than that of Bi_0.8_Sb_0.2_ (012) 10 nm/Cr_1.6_Te_2_ 5 nm (*a*-axis), 0.815×10^6^ A/cm^2^ (150 K), which is attributed to the absence of $\boldsymbol{\sigma}_{\boldsymbol{z}}$. As expected, a reversal of the switching polarity was observed when the polarity of $\boldsymbol{B}_{\boldsymbol{x}}$ was reversed, consistent with conventional SOT systems. These results indicate that further study is highly required to integrate RT PMA FMs directly on Bi_1−_*_x_*Sb*_x_* (012) with in-plane ordering, which is essential for realizing low-power field-free SOT sources.

**Supplementary Note S5. Current-induced magnetization switching in anisotropic three-fold twin domain Bi_1−_*_x_*Sb*_x_* (012)/Cr_1.6_Te_2_**

**5.1	Validation of the non-vanishing** $\boldsymbol{\sigma}_{\boldsymbol{z}}$ **in anisotropic three-fold twin domain**

**Figure S24.** Schematic illustration of anisotropic three-fold twin domain and single-domain of Bi_1−_*_x_*Sb*_x_* (012) 10 nm/Cr_1.6_Te_2_ 5 nm. $a$, $b$, $(1-a-b)$ indicate fractional populations of each domain, respectively.

Here, we demonstrate the presence of a non-vanishing $\boldsymbol{\sigma}_{\boldsymbol{z}}$ in anisotropic three-fold twin domain Bi_1−_*_x_*Sb*_x_* (012)/Cr_1.6_Te_2_ when a current is applied at an angle $\theta$ with respect to the *a*-axis of the #1 α-domain (for the β-domain, −*a*-axis), as illustrated in Figure S24. $\boldsymbol{\sigma}_{\boldsymbol{z}}$ is generated only when the current is applied along the bulk-mirror-symmetry-broken *a*-axis of Bi_1−_*_x_*Sb*_x_* (012); when the current is applied along the *b*-axis, $\boldsymbol{\sigma}_{\boldsymbol{z}}$ is not generated. Therefore, the magnitude of the generated $\boldsymbol{\sigma}_{\boldsymbol{z}}$ scales with the projection of the applied current onto the *a*-axis. The total projection of the applied current onto the *a*-axes of all domains ($I_{a,multi}$) can be expressed as Equation (S13), identical to that in the main manuscript. Here, $\alpha_{i}$ and $\beta_{i}$ denote the fractional populations of the α/β domains within domains #1–#3, respectively, and $\alpha_{i}-\beta_{i}\neq0$, as confirmed by the anisotropic $\varphi$ peaks observed in the XRD $\varphi$-scan data.

$$I_{a,multi}=a\left( \alpha_{1}-\beta_{1} \right)\cos\theta-b\left( \alpha_{2}-\beta_{2} \right)\cos(60^{\circ}-\theta)-(1-a-b)\left( \alpha_{3}-\beta_{3} \right)sin(\theta-30^{\circ})$$

**(S13)**

Our $\varphi$-scan data (Figure 1d and Figure S4) consistently show that either the α- or the β-domain is more prevalent within the multiple rotational variants. Accordingly, at the micro-device scale, the imbalance between the α and β domain fractions can become even more pronounced than in the large-area average probed by XRD. The simplest form to reflect this phenomenon in the $I_{a,multi}$ is to set the imbalance parameter to unity, $\alpha_{i}-\beta_{i}=1$ (or $\beta_{i}-\alpha_{i}=1$, which changes only the polarity of the spin, not its magnitude). This is not a claim that the actual device has a perfectly preferential domain distribution. Assuming $\alpha_{i}-\beta_{i}=1$ for simplicity, Equation (S13) becomes $\mathrm{acos}\theta-\mathrm{bcos}\left( 60^{\circ}-\theta\right)-\left( 1-a-b \right)\sin\left( \theta-30^{\circ} \right)=\left( \frac{3a-1}{2} \right)\cos\theta+$ $\left( \frac{\sqrt{3}}{2} \right)\left( 1-a-2b \right)\sin\theta$, which can be rewritten as Equation (S14), where $C_{A}=$ $\frac{1}{2}\sqrt{\left( 3a-1 \right)^{2}+3\left( 1-a-2b \right)^{2}}$, and $\theta_{\max}$ is the angle $\theta$ that maximizes $I_{a,multi}$. $I_{a,multi}$ is zero only when $a=b=\text{1/3}$, where exact cancellation among the three domains yields $C_{A}= 0\to I_{a,multi}=0$. For comparison, the *a*-axis projection of the current for a single-domain case is $I_{a,single}=cos\theta$ with $\theta_{max}=0^{\circ}$.

$$I_{a,multi}=C_{A}cos(\theta-\theta_{max})$$

**(S14)**

We numerically calculate the angle $\theta_{max}$ that maximizes $I_{a,multi}$ as a function of the domain fractions, as shown in Figure 4b (main manuscript), by differentiating $I_{a,multi}$ with respect to $\theta$. We found that $I_{a,multi}$ exhibits a maximum at $\theta=\theta_{max}$, a minimum at $\theta=\theta_{max}+\text{180°}$, and becomes zero at $\theta=\theta_{max}\pm\text{90°}$, exhibiting the same angular dependence as $I_{a,single}$. This demonstrates that even in the presence of multi-fold in-plane ordering, Bi_1−_*_x_*Sb*_x_* (012) can generate a non-vanishing $\boldsymbol{\sigma}_{\boldsymbol{z}}$ as long as the domain fractions are not perfectly equal, and the angular dependence of $\boldsymbol{\sigma}_{\boldsymbol{z}}$ generation follows that of the single-domain case.

To further evaluate the efficiency of $\boldsymbol{\sigma}_{\boldsymbol{z}}$ generation in anisotropic three-fold twin domains, we calculated the ratio $\frac{I_{a,multi}}{I_{a,single}}$ at $\theta_{max}$ as a function of domain fractions (Figure 4c, main manuscript). Even a 10% deviation of one domain fraction from 1/3 yields $\frac{I_{a,multi}}{I_{a,single}}\approx\text{0.1}$, while a 20% deviation of two domain fractions yields $\frac{I_{a,multi}}{I_{a,single}}\approx\text{0.2}$. Therefore, anisotropic three-fold twin domain Bi_1−_*_x_*Sb*_x_* (012)/Cr_1.6_Te_2_ can effectively serve as a source of $\boldsymbol{\sigma}_{\boldsymbol{z}}$ generation. However, if the $\boldsymbol{\sigma}_{\boldsymbol{z}}$ generation in Bi_1−_*_x_*Sb*_x_* (012) did not originate from bulk mirror-symmetry breaking, the resulting $\frac{I_{a,multi}}{I_{a,single}}$ would be far small to produce a sufficient $\boldsymbol{\sigma}_{\boldsymbol{z}}$ for field-free switching, even when domain anisotropy is non-zero. Thus, bulk-mirror-symmetry-broken materials can act as field-free SOT sources even in the presence of in-plane multi-fold domain ordering. Further exploration of materials exhibiting bulk mirror symmetry breaking is therefore essential for realizing efficient field-free SOT devices. Moreover, we would like to acknowledge that direct real-space mapping of the crystallographic domains would be an important subject for future work, which would significantly strengthen the conclusion. The present $I_{a,multi}$ model is intended to describe the leading symmetry-based behavior extracted from the angle-dependent switching results, rather than to reproduce every microscopic detail of the actual domain arrangement.

**5.2	Current-induced magnetization switching in Bi_1−_*_x_*Sb*_x_* (012) with 0.4** $\text{≤}$ ***x*** $\text{≤}$ **0.8**

**Figure S25.** a) AHE loop of Bi_0.6_Sb_0.4_ (012) 10 nm/Cr_1.6_Te_2_ 5 nm at 150 K. Current-induced magnetization switching of Bi_0.6_Sb_0.4_ (012) 10 nm/Cr_1.6_Te_2_ 5 nm at 150 K under various in-plane magnetic fields and in the absence of a magnetic field, measured along the b) $\varphi=$ 45°, and c) $\varphi=$ 315°, respectively. The blue box indicates the magnetization switching data at *H*_comp_.

Figure S25a shows a clear AHE loop, confirming the presence of PMA in Bi_0.6_Sb_0.4_ (012) 10 nm/Cr_1.6_Te_2_ 5 nm at 150 K, with an AHE resistance of 0.49 $\Omega$. As shown in Figure S25b,c, Bi_0.6_Sb_0.4_ (012) 10 nm/Cr_1.6_Te_2_ 5 nm exhibits field-free magnetization switching when the current is applied along $\varphi=$ 45° and 315° with respect to the sapphire [11$\bar{\text{2}}$0] direction. This behavior is consistent with the non-zero $I_{a,multi}$ calculated in Supplementary Note S5.1, and the fact that $\varphi=$ 45° or 315° does not correspond to $\theta_{max}$ explains why field-free switching occurs for both directions. Moreover, the opposite field-free switching polarities observed for the two current directions indicate that $\theta_{max}\pm90^{\circ}$ lies between 45° and 315°. For the *J*_C_, Bi_0.6_Sb_0.4_ (012) 10 nm/Cr_1.6_Te_2_ 5 nm ($\varphi=$ 45°) shows *J*_C_ of approximately 1.56×10^6^ A/cm^2^ without $\boldsymbol{B}_{\boldsymbol{x}}$ at 150 K. As expected, no reversal of switching polarity under opposite $\boldsymbol{B}_{\boldsymbol{x}}$ polarities is observed for either current direction, confirming the presence of $\boldsymbol{\sigma}_{\boldsymbol{z}}$. The $\boldsymbol{B}_{\boldsymbol{x}}$-assisted magnetization switching data of Bi_0.6_Sb_0.4_ (012) 10 nm/Cr_1.6_Te_2_ 5 nm indicate *H*_comp_ of −8 mT ($\varphi=$ 45°) and −7 mT ($\varphi=$ 315°) at 150 K. Compared with the single-domain Bi_0.8_Sb_0.2_ (012) 10 nm/Cr_1.6_Te_2_ 5 nm heterostructure, these values are significantly smaller. Therefore, to accurately determine *H*_comp_, we performed current-induced magnetization switching measurements under various $\boldsymbol{B}_{\boldsymbol{x}}$, as shown in Figure S25. This notably small *H*_comp_ originates from the very weak out-of-plane SOT generated in Bi_0.6_Sb_0.4_ (012) 10 nm/Cr_1.6_Te_2_ 5 nm, as confirmed by the ST-FMR results in Figure 2d (main manuscript). The field-free magnetization switching ratios of Bi_0.6_Sb_0.4_ (012) 10 nm/Cr_1.6_Te_2_ 5 nm at 150 K are approximately 28.3 % ($\varphi=$ 45°) and 19.0% ($\varphi=$ 315°), while the $\boldsymbol{B}_{\boldsymbol{x}}$-assisted magnetization switching ratios at 150 K are approximately 74.0 % ($\varphi=$ 45°) and 62.6% ($\varphi=$ 315°), respectively.

**Figure S26.** a) AHE loop of Bi_0.4_Sb_0.6_ (012) 10 nm/Cr_1.6_Te_2_ 5 nm at 150 K. Current-induced magnetization switching of Bi_0.4_Sb_0.6_ (012) 10 nm/Cr_1.6_Te_2_ 5 nm (Device #4) at 150 K under various in-plane magnetic fields and in the absence of a magnetic field, measured along the b) $\varphi=$ 180°, and c) $\varphi=$ 270°, respectively.

Figure S26a shows a clear AHE loop, confirming the presence of PMA in Bi_0.4_Sb_0.6_ (012) 10 nm/Cr_1.6_Te_2_ 5 nm (device #4) at 150 K, with an AHE resistance of 0.24 $\Omega$. As shown in Figure S26b,c, Bi_0.4_Sb_0.6_ (012) 10 nm/Cr_1.6_Te_2_ 5 nm exhibits field-free magnetization switching when the current is applied along $\varphi=$ 180° and 270° with respect to the sapphire [11$\bar{\text{2}}$0] direction. This behavior is consistent with the non-zero $I_{a,multi}$ calculated in Supplementary Note S5.1, and the fact that $\varphi=$ 180° or 270° does not correspond to $\theta_{max}$ explains why field-free switching occurs for both directions. Moreover, the opposite field-free switching polarities observed for the two current directions indicate that $\theta_{max}\pm\text{90°}$ lies between 180° and 270°. For the *J*_C_, Bi_0.4_Sb_0.6_ (012) 10 nm/Cr_1.6_Te_2_ 5 nm ($\varphi=$ 270°, device #4) shows *J*_C_ of approximately 1.18×10^6^ A/cm^2^ without $\boldsymbol{B}_{\boldsymbol{x}}$ at 150 K. As expected, no reversal of switching polarity under opposite $\boldsymbol{B}_{\boldsymbol{x}}$ polarities is observed for either current direction, confirming the presence of $\boldsymbol{\sigma}_{\boldsymbol{z}}$. The $\boldsymbol{B}_{\boldsymbol{x}}$-assisted magnetization switching data of Bi_0.4_Sb_0.6_ (012) 10 nm/Cr_1.6_Te_2_ 5 nm indicate *H*_comp_ of −10 mT ($\varphi=$ 180°) and −12 mT ($\varphi=$ 270°) at 150 K. Compared with the single-domain Bi_0.8_Sb_0.2_ (012) 10 nm/Cr_1.6_Te_2_ 5 nm heterostructure, these values are slightly smaller. This small *H*_comp_ originates from weaker out-of-plane SOT generated in Bi_0.4_Sb_0.6_ (012) 10 nm/Cr_1.6_Te_2_ 5 nm, as confirmed by the ST-FMR results in Figure 2d (main manuscript). The field-free magnetization switching ratio of Bi_0.4_Sb_0.6_ (012) 10 nm/Cr_1.6_Te_2_ 5 nm at 150 K is approximately 48.4 % ($\varphi=$ 270°, device #4), while the $\boldsymbol{B}_{\boldsymbol{x}}$-assisted magnetization switching ratio at 150 K is approximately 83.3 % ($\varphi=$ 270°, device #4).

**Figure S27.** a) Absolute values of *H*_comp_ for multiple Bi_0.4_Sb_0.6_ (012) 10 nm/Cr_1.6_Te_2_ 5 nm devices with current applied along various angles $\theta$ at 150 K. Orange bars denote the negative value of *H*_comp_. The numbers on top of each bar indicate the current direction with respect to the sapphire [11$\bar{\text{2}}$0] direction. b) Field-free *J*_C_ of multiple Bi_0.4_Sb_0.6_ (012) 10 nm/Cr_1.6_Te_2_ 5 nm devices with current applied along various angles at 150 K.

The *H*_comp_ and field-free *J*_C_ values of multiple Bi_0.4_Sb_0.6_ (012) 10 nm/Cr_1.6_Te_2_ 5 nm devices (device #1–#4) are summarized in Figure S27a and S27b, respectively. Because two perpendicular current directions were tested in each device, the direction that lies closer to $\theta_{max}$ yields a larger the difference in *H*_comp_ between the two current orientations. When the current is applied exactly along $\theta_{max}$ (as in device #2), one of the two orthogonal directions exhibits neither field-free switching nor a measurable *H*_comp_. In summary, multiple Bi_0.4_Sb_0.6_ (012) 10 nm/Cr_1.6_Te_2_ 5 nm devices (device #1–#4) exhibit *H*_comp_ values in the range of 9.5–16.0 mT and field-free *J*_C_ values of 1.18–1.25×10^6^ A/cm^2^ without $\boldsymbol{B}_{\boldsymbol{x}}$ at 150 K.

**Figure S28.** a) AHE loop of Bi_0.2_Sb_0.8_ (012) 10 nm/Cr_1.6_Te_2_ 5 nm at 150 K. Current-induced magnetization switching of Bi_0.2_Sb_0.8_ (012) 10 nm/Cr_1.6_Te_2_ 5 nm at 150 K under various in-plane magnetic fields and in the absence of a magnetic field, measured along the b) $\varphi=$ 90°, and c) $\varphi=$ 0°, respectively.

Figure S28a shows a clear AHE loop, confirming the presence of PMA in Bi_0.2_Sb_0.8_ (012) 10 nm/Cr_1.6_Te_2_ 5 nm at 150 K, with an AHE resistance of 0.15 $\Omega$. As shown in Figure S28b,c, Bi_0.2_Sb_0.8_ (012) 10 nm/Cr_1.6_Te_2_ 5 nm exhibits field-free magnetization switching when the current is applied along $\varphi=$ 90° and 0° with respect to the sapphire [11$\bar{\text{2}}$0] direction. This behavior is consistent with the non-zero $I_{a,multi}$ calculated in Supplementary Note S5.1, and the fact that $\varphi=$ 90° or 0° does not correspond to $\theta_{max}$ explains why field-free switching occurs for both directions. Moreover, the opposite field-free switching polarities observed for the two current directions indicate that $\theta_{max}\pm\text{90°}$ lies between 90° and 0°. For the *J*_C_, Bi_0.2_Sb_0.8_ (012) 10 nm/Cr_1.6_Te_2_ 5 nm ($\varphi=$ 0°) shows *J*_C_ of approximately 1.21×10^6^ A/cm^2^ without $\boldsymbol{B}_{\boldsymbol{x}}$ at 150 K. As expected, no reversal of switching polarity under opposite $\boldsymbol{B}_{\boldsymbol{x}}$ polarities is observed for either current direction, confirming the presence of $\boldsymbol{\sigma}_{\boldsymbol{z}}$. The $\boldsymbol{B}_{\boldsymbol{x}}$-assisted magnetization switching data of Bi_0.2_Sb_0.8_ (012) 10 nm/Cr_1.6_Te_2_ 5 nm indicate *H*_comp_ values of 13 mT ($\varphi=$ 90°) and −14 mT ($\varphi=$ 0°) at 150 K. Compared with the single-domain Bi_0.8_Sb_0.2_ (012) 10 nm/Cr_1.6_Te_2_ 5 nm heterostructure, these values are slightly smaller. This reduced *H*_comp_ originates from the weaker out-of-plane SOT generated in Bi_0.2_Sb_0.8_ (012) 10 nm/Cr_1.6_Te_2_ 5 nm, as confirmed by the ST-FMR results in Figure 2d (main manuscript). The field-free magnetization switching ratio of Bi_0.2_Sb_0.8_ (012) 10 nm/Cr_1.6_Te_2_ 5 nm at 150 K is approximately 34.3 % ($\varphi=$ 0°), while the $\boldsymbol{B}_{\boldsymbol{x}}$-assisted magnetization switching ratio at 150 K is approximately 79.2 % ($\varphi=$ 0°).

**Supplementary Note S6. Estimation of thermal effects induced by pulsed current in magnetization switching**

Heating induced by the pulsed current can temporarily reduce the energy barrier for switching, lower the coercivity, and facilitate domain depinning and propagation. Such thermal effects would be expected to become much more pronounced if a constant direct current were used for switching or transport measurements, as in methods such as loop-shift measurements. We would like to emphasize that, in our case, short current pulses of up to 100 μs were used, and the current density was kept below 2 $\times$ 10^6^ A/cm^2^ in all measurements. Moreover, even compared with previous studies (Figure 4g), the switching in our work was achieved at one of the lowest current densities reported. Furthermore, a thermocouple is installed in our PPMS holder near the sample puck, and this sensor showed no measurable temperature change during the switching measurements. Considering thermal diffusion, if significant heat accumulation had occurred even under pulsed-current operation, at least some detectable temperature response at the nearby thermocouple would be expected. However, this was not observed in our case. Nevertheless, to quantitatively evaluate the thermal contribution, we estimated the transient temperature rise using a semi-infinite heat-diffusion model and independently extracted the temperature increase from the resistance change based on the resistance–temperature (*R*–*T*) curve.

**Theoretical calculation of the transient temperature rise during the switching pulse.**

To quantitatively evaluate the thermal assistance from the pulsed current, we first estimated the transient temperature rise ($\Delta T$) during the switching pulse using a semi-infinite heat-diffusion model for the sapphire substrate.^[25]^ For sapphire near 150 K, we used the thermal conductivity ($k\approx\text{115 }\text{W∙}\text{m}^{\text{-1}}\text{K}^{\text{-1}}$), mass density ($\rho_{m}\approx\text{3.987×}\text{10}^{\text{3}}\text{ kg/}\text{m}^{\text{3}}$), and specific heat ($C_{p}\approx\text{31.94 J} \text{mol}^{\text{-1}}\text{K}^{\text{-1}}\text{ \textasciitilde313 J} \text{kg}^{\text{-1}}\text{K}^{\text{-1}}$) values.^[26,27]^ Under a constant surface heat flux, the surface temperature of a semi-infinite solid increases with the square root of time according to the classical Carslaw–Jaeger solution. Using our device parameters for a representative Bi_0.8_Sb_0.2_ (012)/Cr_1.6_Te_2_ device (current$I$ = 3 mA, which is slightly larger than the critical switching current; resistivity $\rho_{xx}$ = 3.70$\times{10}^{-6} \Omega\cdot m$ at 150 K; channel length $L$ = 120 μm; width $w$ = 20 μm; and total conducting thickness $t$ *=* 15 nm), the channel resistance is estimated as $R=\frac{\rho_{xx}L}{w\cdot t}\approx\text{1.48 kΩ}$. The experimentally measured channel resistance, including contact resistance, ranges from 1.9 k$\Omega$ to 2.7 k$\Omega$ depending on the device and the concentration of Bi_1−_*_x_*Sb*_x_*. The Joule power during the pulse is therefore $P=I^{2}R\approx\text{13.3 mW}$, which corresponds to an areal heat flux of $q^{''}=P/(w\cdot L)\approx\text{5.55×}\text{10}^{\text{6}}\text{ W/}\text{m}^{\text{2}}$ over the channel area. Using the sapphire thermal diffusivity $\alpha=\frac{k}{\rho_{m}\cdot C_{p}}\approx9.2\times{10}^{-5} m^{2}/s$, the transient surface temperature rise for a 100 μs pulse is estimated from $\Delta T\left( 0, 100 \mu s \right)\approx\frac{2q^{''}}{k}\sqrt{\frac{\alpha\cdot\left( 100 \mu s \right)}{\pi}}$ to be only ~ 5.2 K. Thus, even at 150 K, the pulse-induced heating is expected to raise the local temperature only to about 155 K. This indicates that Joule heating can provide only limited thermal assistance rather than being the primary origin of the observed switching. In particular, if the effective anisotropy energy $K_{eff}$ and the effective reversal volume do not change appreciably between 150 and 155 K, the thermal stability factor $\Delta=\frac{K_{eff}V}{k_{B}T}$, where $k_{B}$ is the Boltzmann constant, would decrease only by a factor of 150/155 $\approx$ 0.968, corresponding to a reduction of about 3.2%. Therefore, the estimated transient heating is expected to make only a modest contribution to lowering the switching barrier.

In addition, the duty cycle in our measurements is extremely small (a 100 μs pulse every 2 seconds, i.e. 5$\times$10^-5^), giving an average dissipated power of only ~ 0.67 μW. From the same thermal diffusivity, the characteristic thermal diffusion time across the 750 μm sapphire substrate is on the order of (750 μm)^2^/$\alpha$ ~ 6.1 ms, which is far shorter than the 2 s interval between pulses. Therefore, cumulative heating from pulse to pulse is negligible, which is consistent with our thermocouple measurements. Based on this estimate, we conclude that the thermal effect of the pulsed current is secondary and is insufficient by itself to account for the observed field-free magnetization switching.

**Estimation of transient temperature rises using the *R*–*T* curve.**

 **Figure S29.** Experimental estimation of the transient temperature rise in Bi_1−_*_x_*Sb*_x_* (012) 10 nm/Cr_1.6_Te_2_ 5 nm devices using resistance thermometry. a) *R*–*T* curves of Bi_1−_*_x_*Sb*_x_* (012) 10 nm/Cr_1.6_Te_2_ 5 nm as a function of Sb concentration near 150 K, used to extract the local slope $\left( \frac{dR}{dT} \right)_{150 K}$. b) $\Delta T$ as a function of current density, obtained by converting the pulse-induced resistance change using the measured *R*–*T* curves. The dotted vertical lines indicate the representative current densities used for the temperature evaluation.

**Table S3. Resistance at 150 K, local slope (*dR/dT*)_150 K_, and estimated** $\boldsymbol{\Delta T}$ **for Bi_1−_*_x_*Sb*_x_* (012) 10 nm/Cr_1.6_Te_2_ 5 nm devices.**

| Concentration | *R*(150 K) [Ω] | (*dR/dT*)_150 K_ [Ω/K] | $\boldsymbol{\Delta T}$  [K] |
| --- | --- | --- | --- |
| *x* = 0.1 | 2740.1 | -2.42 | 7.901 (10^6^ A/cm^2^) |
| *x* = 0.2 | 2450.1 | -2.01 | 6.729 (10^6^ A/cm^2^) |
| *x* = 0.3 | 2446.6 | -0.58 | 6.039 (10^6^ A/cm^2^) |
| *x* = 0.4 | 2319.8 | 0.40 | 10.86 (1.6 $\times$ 10^6^ A/cm^2^) |
| *x* = 0.6 | 2053.9 | 2.91 | 10.82 (1.6 $\times$ 10^6^ A/cm^2^) |
| *x* = 0.8 | 1906.9 | 3.85 | 9.08 (1.6 $\times$ 10^6^ A/cm^2^) |

We further estimated the transient temperature rise using the measured *R*–*T* curve, including contact resistance, for each device.

1. First, the device resistance was measured as a function of temperature under a small sensing current (100 μA), and the resulting $R(T)$curve was used as a calibration curve.
2. Second, for each pulse event, we defined the base resistance at 150 K as $R_{0}=R(\text{150 K})$, and the resistance during the pulse event as $R_{i}$.
3. Third, we measured $R_{i}$ 75 μs after the onset of the pulsed current. During the pulse measurements, the resistance change ($\Delta R=R_{i}-R_{0})$ associated with each pulse event was converted into an effective temperature rise ($\Delta T$) using the local slope of the *R*–*T* curve near 150 K ($\left( \frac{dR}{dT} \right)_{\text{150 K}}$), according to $\Delta T\approx\Delta R/\left( \frac{dR}{dT} \right)_{\text{150 K}}$.

Because the switching measurement and the *R*–*T* curve calibration were performed on the same patterned Hall-bar device, the total measured resistance, including contact resistance, was used consistently for both the calibration and the pulse-induced resistance analysis. Therefore, although the absolute resistance contains a contact contribution, the temperature estimation remains self-consistent for each individual device.

As shown in Figure S29 and Table S3, the estimated transient temperature rise ranges from approximately 6.0 to 10.9 K depending on the Sb concentration and current density. For the single-domain-dominant Bi_1−_*_x_*Sb*_x_* (012) with *x* = 0.1–0.3, where *J*_C_ was less than 10^6^ A/cm^2^, $\Delta T$ was estimated to be 6.0–7.9 K at a current density of 10^6^ A/cm^2^. For the multi-domain Bi_1−_*_x_*Sb*_x_* (012) with *x* = 0.4–0.8, where *J*_C_ was in the range of 1–1.6 $\times$ 10^6^ A/cm^2^, $\Delta T$ was estimated to be 9.1–10.9 K at a current density of 1.6 $\times$ 10^6^ A/cm^2^. These experimentally estimated values ($\Delta T\approx$ 6.7 K at *J*_C_ = 10^6^ A/cm^2^ for *x* = 0.2) are of the same order as the transient heating obtained from the semi-infinite heat-diffusion model ($\Delta T\approx$ 5.2 K at *I* = 3 mA (*J*_C_ = 0.968 $\times$ 10^6^ A/cm^2^) for *x* = 0.2) and indicate that the pulse-induced temperature increase remains within a relatively narrow range around 150 K. In addition, the *M*–*T* curves of Cr_1.6_Te_2_ shown in Figure S18 indicate that $M_{\mathbf{S}}$ does not vary significantly within 150 K $\pm$10 K. Thus, even if pulsed-current-induced heating causes a temperature increase of approximately 10 K near 150 K, such heating is not expected to substantially reduce the switching energy barrier. Furthermore, if thermal effects induced by the pulsed current played a dominant role in the field-free magnetization switching, the field-free switching data shown in Figure S19, S20, and S22, where the current was applied along the *b*-axis, should also exhibit switching loops. However, such behavior was not observed.

In conclusion, although Joule heating may provide finite thermal assistance to the switching process, both the thermal model and the resistance-based temperature estimation suggest that thermal effects induced by the pulsed current do not play a dominant role in the field-free magnetization switching. Rather, they may provide only a minor auxiliary contribution by slightly lowering the switching energy barrier over a very short time scale.

**Supplementary Note S7. Summary of the current-induced magnetization switching in Bi_1−_*_x_*Sb*_x_* (012)/Cr_1.6_Te_2_**

**Table S4. Summary of resistivity, *J*_C_ and *P*_SOT_ for spintronic materials**

| **Sample** | Resistivity[$\mu\Omega\cdot cm$] | *J*_C_[10^6^ A/cm^2^] | *P*_SOT_ [10^14^ W/m^3^] |
| --- | --- | --- | --- |
| **Heavy metal** |  |  |  |
| Pt/Co^[28]^ Pt/FGT (180 K)^[29]^ Pt/CrTe_2_ (150 K)^[30]^ | 33.0 34.0 16.0 | 23.0 15.0 24.0 | 175 76.5 92.2 |
| *β*-W/CFB^[31]^ | 170 | 18.0 | 551 |
| Ta/CoTb^[32]^ | 193 | 8.0 | 124 |
|  |  |  |  |
| **Topological materials** |  |  |  |
| Bi*_x_*Se_(1−_*_x_*_)_^[33]^ | 78000 | 0.43 | 144 |
| Bi_0.9_Sb_0.1_/MnGa^[6]^ | 400 | 1.50 | 13.5 |
| Bi_2_Se_3_/CoTb^[32]^ | 1060 | 3.00 | 104 |
|  |  |  |  |
| **Low-symmetry materials** |  |  |  |
| WTe_2_/CFB (*a*-axis)^[34]^ WTe_2_/CrTe_2_ (*a-*axis)^[30]^ | 410 1500 | 1.95 1.20 | 15.6 21.6 |
| TaIrTe_4_/CFB (*a*-axis)^[16]^ TaIrTe_4_/CFB (*a*-axis)^[34]^ | 209 204 | 7.65 2.35 | 122 11.2 |
|  |  |  |  |
| **Bi_1−_*_x_*Sb*_x_* (012)/Cr_1.6_Te_2_ (this work, 150 K)** |  |  |  |
| $x=$ 0.1 (*a*-axis) $x=$ 0.2 (*a*-axis) $x=$ 0.3 (*a*-axis) $x=$ 0.4 $x=$ 0.6 $x=$ 0.8 | 469 382 326 243 171 139 | 1.00 0.815 0.926 1.56 1.18  1.21 | 11.0  5.32 5.84 9.98 3.53 2.85 |
|  |  |  |  |

**Table S5. Summary of** $\boldsymbol{M}_{\mathbf{S}}$**,** $\boldsymbol{H}_{\boldsymbol{k,}\mathbf{eff}}$ **and *J*_C_**$\boldsymbol{/}\boldsymbol{\mu}_{\boldsymbol{0}}{\boldsymbol{M}_{\mathbf{S}}\boldsymbol{H}}_{\boldsymbol{k}\boldsymbol{,}\mathbf{eff}}$ **of Bi_1−_*_x_*Sb*_x_* (012)/Cr_1.6_Te_2_ (150 K)**

| **Bi_1−_*_x_*Sb*_x_* (012)/Cr_1.6_Te_2_ (150 K)** | $M_{S}$[10^3^ A/m] | ${\mu_{0}H}_{k,\mathrm{eff}}$[T] | *J*_C_$/\mu_{0}{M_{S}H}_{k,\mathrm{eff}}$[10^4^ A$\cdot$m/J] |
| --- | --- | --- | --- |
| $x=$ 0.1  $x=$ 0.2  $x=$ 0.3  $x=$ 0.4 $x=$ 0.6 $x=$ 0.8 | 176.9 178.1 174.6 176.2 171.4 173.7 | 2.18 2.17 2.13 2.12 2.14  2.24 | 2.59 2.11 2.49 4.18 3.22  3.11 |

To compare the switching efficiency across different compositions while accounting for differences in perpendicular magnetic anisotropy and magnetic stability, we evaluated a figure of merit defined as *J*_C_$/\mu_{0}{M_{S}H}_{k,\mathrm{eff}}$, where *J*_C_ is the critical switching current density, $M_{S}$ is the saturation magnetization, and $H_{k,\mathrm{eff}}$ is the effective anisotropy field, obtained from Figure S18b. Given that $\frac{1}{2}\mu_{0}{M_{S}H}_{k,\mathrm{eff}}$ corresponds to the effective anisotropy energy density scale, and that the thickness of Cr_1.6_Te_2_ was fixed for all samples, this normalization partially reduces the effect of magnetic hardness on the variation in *J*_C_. Therefore, a smaller value of *J*_C_$/\mu_{0}{M_{S}H}_{k,\mathrm{eff}}$ indicates more efficient current-induced switching relative to the anisotropy energy scale of the magnetic layer. As shown in Table S5, the comparison using *J*_C_$/\mu_{0}{M_{S}H}_{k,\mathrm{eff}}$ allows a more direct evaluation of the switching efficiency by partially separating the influence of magnetic stability from the current-driven switching process.

**References**

[1]	Fukumoto, N. *et al*. Observation of large spin conversion anisotropy in bismuth. *Proc. Natl Acad. Sci.* **120**, e2215030120 (2023)

[2]	Liang, H.-L. *et al*. Anisotropic spin-to-charge conversion in bismuth. *Phys. Rev. B* **106**, L201304 (2022).

[3]	Kim, S., Lee, H.-W. & Choi, G.-M. Giant Spin‐Orbit Torque in Sputter‐Deposited Bi Films. *Adv. Sci.* **10**, 2303831 (2023).

[4]	Rho, S. *et al.* Colossal Spin Hall Conductivity in Charge-Massive Topologically Nontrivial Semimetal Phase of Bi_1–_*_x_*Sb*_x_* (012). *ACS Nano* **19**, 38749-38759 (2025).

[5]	Walsh, L. A. & Hinkle, C. L. van der Waals epitaxy: 2D materials and topological insulators. *Appl. Mater. Today* **9**, 504-515 (2017).

[6]	Hofmann, P. The surfaces of bismuth: Structural and electronic properties. *Prog. Surf. Sci.* **81**, 191-245 (2006).

[7]	Khang, N. H. D., Ueda, Y. & Hai, P. N. A conductive topological insulator with large spin Hall effect for ultralow power spin–orbit torque switching. *Nat. Mater.* **17**, 808-813 (2018).

[8]	Khang, N. H. D., Nakano, S., Shirokura, T., Miyamoto, Y. & Hai, P. N. Ultralow power spin–orbit torque magnetization switching induced by a non-epitaxial topological insulator on Si substrates. *Sci. Rep.* **10**, 12185 (2020).

[9]	Zhu, X.-G. *et al*. Three Dirac points on the (110) surface of the topological insulator Bi_1−x_Sb_x_. *New J. Phys.* **15**, 103011 (2013).

[10]	Yao, K., Khang, N. H. D. & Hai, P. N. Influence of crystal orientation and surface termination on the growth of BiSb thin films on GaAs substrates. *J. Cryst. Growth* **511**, 99-105 (2019).

[11]	Jeffrey, C. A., Zheng, S. H., Bohannan, E., Harrington, D. A. & Morin, S. X-ray characterization of as-deposited, epitaxial films of Bi (012) on Au (111). *Surf. Sci.* **600**, 95-105 (2006).

[12]	Chaluvadi, S. K. *et al.* Uncovering the Lowest Thickness Limit for Room-Temperature Ferromagnetism of Cr_1.6_Te_2_. *Nano Lett.* **24**, 7601-7608 (2024).

[13]	Rho, S. *et al.* Designing Nontrivial Real‐Space Berry Curvature through Non‐Monotonic Bulk Inversion Symmetry Breaking in Self‐Intercalated Cr_1+δ_Te_2_. *Small Sci.* **5**, 2500028 (2025).

[14]	Wang, Y., Ramaswamy, R. & Yang, H. FMR-related phenomena in spintronic devices. *J. Phys. D: Appl. Phys.* **51**, 273002 (2018).

[15]	MacNeill, D. *et al.* Control of spin–orbit torques through crystal symmetry in WTe_2_/ferromagnet bilayers. *Nat. Phys.* **13**, 300-305 (2017).

[16]	Shi, S. *et al.* Observation of the out‐of‐plane polarized spin current from CVD grown WTe_2_. *Adv. Quantum Technol.* **4**, 2100038 (2021).

[17]	Liu, Y. *et al.* Field-free switching of perpendicular magnetization at room temperature using out-of-plane spins from TaIrTe_4_. *Nat. Electron.* **6**, 732-738 (2023).

[18]	Wang, F. *et al.* Field-free switching of perpendicular magnetization by two-dimensional PtTe_2_/WTe_2_ van der Waals heterostructures with high spin Hall conductivity. *Nat. Mater.* **23**, 768-774 (2024).

[19]	Manchon, A. *et al*. Current-induced spin-orbit torques in ferromagnetic and antiferromagnetic systems. *Rev. Mod. Phys.* **91**, 035004 (2019).

[20]	Sinova, J., Valenzuela, S. O., Wunderlich, J., Back, C. H. & Jungwirth, T. Spin Hall effects. *Rev. Mod. Phys.* **87**, 1213-1260 (2015).

[21]	Wang, Y., Deorani, P., Qiu, X., Kwon, J. H. & Yang, H. Determination of intrinsic spin Hall angle in Pt. *Appl. Phys. Lett.* **105**, 152412 (2014).

[22]	Liu, L., Moriyama, T., Ralph, D. C. & Buhrman, R. Spin-Torque Ferromagnetic Resonance Induced by the Spin Hall Effect. *Phys. Rev. Lett.* **106**, 036601 (2011).

[23]	McGuire, T. & Potter, R. Anisotropic magnetoresistance in ferromagnetic 3d alloys. *IEEE Trans. Magn.* **11**, 1018-1038 (1975).

[24]	Park, H. *et al.* Topological Surface‐Dominated Spintronic THz Emission in Topologically Nontrivial Bi_1−_*_x_*Sb*_x_* Films. *Adv. Sci.* **9**, 2200948 (2022).

[25]	Carslaw, H. S. & Jaeger, J. C. Conduction of Heat in Solids. *Clarendon, Oxford* (1959).

[26]	Archer, D. G. Thermodynamic properties of synthetic sapphire (α‐Al_2_O_3_), standard reference material 720 and the effect of temperature‐scale differences on thermodynamic properties. *J. Phys. Chem. Ref. Data* **22**, 1441-1453 (1993).

[27]	Dobrovinskaya, E. R., Lytvynov, L. A. & Pishchik, V. Properties of Sapphire. In: Sapphire. Micro- and Opto-Electronic Materials, Structures, and Systems. *Springer* (2009).

[28]	Liu, L., Lee, O. J., Gudmundsen, T. J., Ralph, D. C. & Buhrman, R. A. Current-Induced Switching of Perpendicularly Magnetized Magnetic Layers Using Spin Torque from the Spin Hall Effect. *Phys. Rev. Lett.* **109**, 096602 (2012).

[29]	Alghamdi, M. *et al.* Highly efficient spin–orbit torque and switching of layered ferromagnet Fe_3_GeTe_2_. *Nano Lett.* **19**, 4400-4405 (2019).

[30]	Shi, G. *et al.* Field-free manipulation of two-dimensional ferromagnet CrTe_2_ by spin–orbit torques. *Nano Lett.* **24**, 7302-7310 (2024).

[31]	Pai, C.-F. *et al.* Spin transfer torque devices utilizing the giant spin Hall effect of tungsten. *Appl. Phys. Lett.* **101**, 122404 (2012).

[32]	Han, J. *et al.* Room-temperature spin-orbit torque switching induced by a topological insulator. *Phys. Rev. Lett.* **119**, 077702 (2017).

[33]	Mahendra, DC. *et al.* Room-temperature high spin–orbit torque due to quantum confinement in sputtered Bi*_x_*Se_(1–_*_x_*_)_ films. *Nat. Mater.* **17**, 800-807 (2018).

[34]	Kajale, S. N., Nguyen, T., Hung, N. T., Li, M. & Sarkar, D. Field-free deterministic	switching of all–van der Waals spin-orbit torque system above room temperature. *Sci.	Adv.* **10**, eadk8669 (2024).
